# Supplementary material for: Enhanced recovery following hip and knee arthroplasty: a systematic review of cost-effectiveness evidence
Source: BMJ Open. 2020 Jan 15;10(1):e032204. doi: 10.1136/bmjopen-2019-032204 (PMC7044879; doi:10.1136/bmjopen-2019-032204)
Supplement: Supplementary data [file bmjopen-2019-032204supp001.pdf]

Supplemental Material: Enhanced recovery following hip and knee arthroplasty a systematic review of cost-effectiveness evidence. BMJ Open

Table A1. Full search terms for databases included in this review

Query	Search term
Ovid MEDLINE	
1	arthroplasty, replacement, hip/ or arthroplasty, replacement, knee/
2	((knee? or hip) adj (replace$ or arthroplast*)).ti,ab.
3	1 or 2
4	simulation model$.ti,ab.
5	markov.ti,ab.
6	monte carlo.ti,ab.
7	decision tree$.ti,ab.
8	decision analy$.ti,ab.
9	qaly$.ti,ab.
10	(valu$ adj2 quality).ti,ab.
11	utility value$.ti,ab.
12	((disability or quality) adj adjusted).ti,ab.
13	((life adj2 year$) or health year equivalent$).ti,ab.
14	(health adj utilit$).ti,ab.
15	hui$1.ti,ab.
16	(quality adj3 well$).ti,ab.
17	qwb.ti,ab.
18	(qald$ or qale$ or qtime$).ti,ab.
19	(well being or wellbeing).tw.
20	(health adj2 stat$).tw.
21	((adjusted adj2 life) or qaly$).ti,ab.
22	(daly or qol or hql or hqol or hrqol or hr ql or hrql).tw.
23	cost-utility.ti,ab.
24	cost-effectiveness.ti,ab.
25	cost-benefit.ti,ab.
26	cost-minimisation.ti,ab.
27	cost-minimization.ti,ab.
28	modelling.ti,ab.
29	modeling.ti,ab.
30	decision model.ti,ab.
31	QALY.ti,ab.
32	quality adjusted life year$.ti,ab.
33	cost.ti,ab.
34	life year$.ti,ab.
35	incremental cost-effectiveness ratio.ti,ab.
36	(quality adj2 life).ti,ab.
37	Technology Assessment, Biomedical/
38	"Costs and Cost Analysis"/
39	technology assessment$.ti,ab.
40	economic evaluation$.ti,ab.
41	economic model$.ti,ab.
42	discrete event simulat$.ti,ab.
43	cost utility.ti,ab.
44	cost effectiv$.ti,ab.
45	cost benefit.ti,ab.
46	cost minimisation.ti,ab.
47	cost minimization.ti,ab.
48	ICER$.ti,ab.
49	EQ-5D$.ti,ab.
50	(SF-12 or SF12 or Short Form 12).ti,ab.
51	(SF-36 or SF36 or Short Form 36).ti,ab.
52	(SF-6D or SF6D or Short Form 6D).ti,ab.

Pritchard MG, *et al. BMJ Open* 2020; 10:e032204. doi: 10.1136/bmjopen-2019-032204

Supplemental Material: Enhanced recovery following hip and knee arthroplasty a systematic review of cost-effectiveness evidence. BMJ Open

Query	Search term
53	rosser index.ti,ab.
54	person trade off.ti,ab.
55	standard gamble.ti,ab,kw.
56	time trade off.ti,ab,kw.
57	Hye.ti,ab,kw.
58	Hyes.ti,ab,kw.
59	Euroquol.ti,ab,kw.
60	4 or 5 or 6 or 7 or 8 or 9 or 10 or 11 or 12 or 13 or 14 or 15 or 16 or 17 or 18 or 19 or 20 or 21 or 22 or 23 or 24 or 25 or 26 or 27 or 28 or 29 or 30 or 31 or 32 or 33 or 34 or 35 or 36 or 37 or 38 or 39 or 40 or 41 or 42 or 43 or 44 or 45 or 46 or 47 or 48 or 49 or 50 or 51 or 52 or 53 or 54 or 55 or 56 or 57 or 58 or 59
61	3 and 60
EMBASE	
1	hip replacement/ or hip arthroplasty/
2	total knee replacement/ or knee replacement/ or knee arthroplasty/
3	((knee? or hip) adj (replace$ or arthroplast$)).ti,ab.
4	1 or 2 or 3
5	simulation model$.ti,ab.
6	markov.ti,ab.
7	monte carlo.ti,ab.
8	decision tree$.ti,ab.
9	decision analy$.ti,ab.
10	qaly$.ti,ab.
11	(valu$ adj2 quality).ti,ab.
12	utility value$.ti,ab.
13	((disability or quality) adj adjusted).ti,ab.
14	((life adj2 year$) or health year equivalent$).ti,ab.
15	hui$1.ti,ab.
16	(quality adj3 well$).ti,ab.
17	qwb.ti,ab.
18	(qald$ or qale$ or qtime$).ti,ab.
19	(well being or wellbeing).tw.
20	(health adj2 stat$).tw.
21	((adjusted adj2 life) or qaly$).ti,ab.
22	(daly or qol or hql or hqol or hrqol or hr ql or hrql).tw.
23	cost-utility.ti,ab.
24	cost-benefit.ti,ab.
25	cost-minimisation.ti,ab.
26	cost-minimization.ti,ab.
27	modelling.ti,ab.
28	modeling.ti,ab.
29	QALY.ti,ab.
30	quality adjusted life year$.ti,ab.
31	cost.ti,ab.
32	life year$.ti,ab.
33	incremental cost-effectiveness ratio.ti,ab.
34	(quality adj2 life).ti,ab.
35	decision model$.ti,ab.
36	cost-effectiv$.ti,ab.
37	"cost benefit analysis"/
38	biomedical technology assessment/
39	technology assessment$.ti,ab.
40	economic evaluation$.ti,ab.
41	economic model$.ti,ab.
42	discrete event simulat$.ti,ab.

Pritchard MG, *et al. BMJ Open* 2020; 10:e032204. doi: 10.1136/bmjopen-2019-032204

Supplemental Material: Enhanced recovery following hip and knee arthroplasty a systematic review of cost-effectiveness evidence. BMJ Open

Query	Search term
43	cost utility.ti,ab.
44	cost effectiv$.ti,ab.
45	cost benefit.ti,ab.
46	cost minimisation.ti,ab.
47	cost minimization.ti,ab.
48	ICER$.ti,ab.
49	(health adj utilit$).ti,ab.
50	EQ-5D$.ti,ab.
51	(SF-12 or SF12 or Short Form 12).ti,ab.
52	(SF-36 or SF36 or Short Form 36).ti,ab.
53	(SF-6D or SF6D or Short Form 6D).ti,ab.
54	rosser index.ti,ab.
55	person trade off.ti,ab.
56	standard gamble.ti,ab,kw.
57	time trade off.ti,ab,kw.
58	Hye.ti,ab,kw.
59	Hyes.ti,ab,kw.
60	Euroquol.ti,ab,kw.
61	5 or 6 or 7 or 8 or 9 or 10 or 11 or 12 or 13 or 14 or 15 or 16 or 17 or 18 or 19 or 20 or 21 or 22 or 23 or 24 or 25 or 26 or 27 or 28 or 29 or 30 or 31 or 32 or 33 or 34 or 35 or 36 or 37 or 38 or 39 or 40 or 41 or 42 or 43 or 44 or 45 or 46 or 47 or 48 or 49 or 50 or 51 or 52 or 53 or 54 or 55 or 56 or 57 or 58 or 59 or 60
62	4 and 61
National Health Service Economic Evaluations Database, via the Cochrane Library	
Hip	Title, Abstract, Keywords: “Hip arthroplasty” OR
	Title, Abstract, Keywords: “Hip arthroplasties” OR
	Title, Abstract, Keywords: “Hip replacement”
Knee	Title, Abstract, Keywords: “Knee arthroplasty” OR
	Title, Abstract, Keywords: “Knee arthroplasties” OR
	Title, Abstract, Keywords: “Knee replacement”
EconLit	
	TI,AB(hip) OR TI,AB(knee) AND
	TI,AB(Replace*) OR TI,AB(arthroplasty*) OR TI,AB(Replacement) OR TI,AB(arthroplasties)

Supp.3

Supplemental Material: Enhanced recovery following hip and knee arthroplasty a systematic review of cost-effectiveness evidence. BMJ Open

Table A2. Hierarchy used to classify data sources used in studies, modified from Cooper and colleagues[34] and Coyle and colleagues[35]

*Source of data for clinical effect sizes, adverse events & complications**	
1	Meta-analysis of RCTs with direct comparison between comparator therapies, measuring final outcomes
2	Single RCT with direct comparison between comparator therapies, measuring final outcomes
3	Meta-analysis of RCTs with direct comparison between comparator therapies, measuring surrogate outcomes; *or*<br>meta-analysis of placebo-controlled RCTs with similar trial populations, measuring final outcomes for each individual therapy
4	Single RCT with direct comparison between comparator therapies, measuring surrogate outcomes; *or* single placebo-controlled RCTs with similar trial populations, measuring final outcomes for each individual therapy
5	Meta-analysis of placebo-controlled RCTs with similar trial populations, measuring surrogate outcomes
6	Single placebo-controlled RCTs with similar trial populations, measuring surrogate outcomes for each individual therapy
7	Case–control or cohort studies
8	Non-analytic studies, for example, case reports, case series
9	Expert opinion
*Source of baseline clinical data**	
1	Case series or analysis of reliable administrative databases specifically conducted for the study covering patients solely from the jurisdiction of interest
2	Recent case series or analysis of reliable administrative databases covering patients solely from the jurisdiction of interest
3	Recent case series or analysis of reliable administrative databases covering patients solely from another jurisdiction
4	Old case series or analysis of reliable administrative databases; *or*<br>estimates from RCTs
5	Estimates from previously published economic analyses: unsourced
6	Expert opinion
*Source of data for duration of primary effect**	
1	Analysis of reliable administrative databases specifically conducted for the study covering patients solely from the jurisdiction of interest
2	Recent analysis of reliable administrative databases covering patients solely from the jurisdiction of interest
3	Recent analysis of reliable administrative databases covering patients solely from another jurisdiction
4	Old analysis of reliable administrative databases
5	Estimates from previously published economic analyses: unsourced
6	Expert opinion
*Source of data for resource use**	
1	Prospective data collection; *or*<br>analysis of reliable administrative data from same jurisdiction for specific study
2	Recently published results of prospective data collection from the same; *or*<br>recent analysis of reliable administrative data from the same jurisdiction
3	Unsourced data from previous economic evaluations from the same jurisdiction
4	Recently published results of prospective data collection from a different jurisdiction; *or*<br>recent analysis of reliable administrative data from a different jurisdiction
5	Unsourced data from previous economic evaluation from a different jurisdiction
6	Expert opinion
Source of data for costs	
1	Cost calculations based on reliable databases or data sources conducted for specific study—same jurisdiction
2	Recently published cost calculations based on reliable databases or data sources—same jurisdiction

Supplemental Material: Enhanced recovery following hip and knee arthroplasty a systematic review of cost-effectiveness evidence. BMJ Open

3	Unsourced data from previous economic evaluation—same jurisdiction
4	Recently published cost calculations based on reliable databases or data sources—different jurisdiction
5	Unsourced data from previous economic evaluation—different jurisdiction
6	Expert opinion
Source of data for utilities	
1	Direct utility assessment for the specific study from a sample either:<br>of the general population, or<br>with knowledge of the disease(s) of interest, or<br>of patients with the disease(s) of interest; *or*<br>indirect utility assessment for the specific study from patient sample with disease(s) of interest, using a tool validated for the patient population
2	Direct utility assessment from a previous study from a sample either:<br>of the general population, or<br>with knowledge of the disease(s) of interest, or<br>of patients with the disease(s) of interest; *or*<br>indirect utility assessment from a previous study from patient sample with disease(s) of interest, using a tool validated for the patient population
3	Indirect utility assessment from a patient sample with disease(s) of interest, using a tool not validated for the patient population; *or*<br>patient preference values obtained from a visual analogue scale
4	Delphi panels; *or*<br>expert opinion

* Hierarchy used for assessment of models only.
RCT, randomized controlled trial

Supplemental Material: Enhanced recovery following hip and knee arthroplasty a systematic review of cost-effectiveness evidence. BMJ Open

Table A3. Overview of studies included in the review.

Authors	Population	Perspective	Horizon	Discounting	Currency, year
**Enhanced recovery pathway**					
Brunenberg 2005[37]	Patients on the waiting list for either a THA (48 patients recruited to Joint Recovery Programme (JRP), 50 to usual care) or TKA (30 patients to JRP, 32 to usual care), mean age 64.4 years, one-third male	Societal	1 year	None	USD, 2002
Larsen 2009[38]	Patients in trial[50] having THA, THA or UKA, mean age having accelerated intervention 64, 26 women, 30 men	Societal	1 year	None	DKK, 2006
**Preoperative:** *Assessment and optimization of comorbidities*					
McLawhorn 2016[39]	50-year-old morbidly obese patient with end-stage unilateral knee osteoarthritis	Societal	40 years	3%	USD, 2012
**Preoperative:** *Exercise*					
Fernandes 2017[40]	Adults having unilateral THA or TKA for symptomatic osteoarthritis	Healthcare sector	61 weeks	None	EUR, 2012
**Preoperative:** *Staphylococcus aureus prophylaxis*					
Courville 2012[41]	65-year-olds with end stage hip or knee osteoarthritis for whom medical management has failed and TJA recommended.	Societal	1 year	None	USD, 2005
**Intraoperative:** *Avoid unnecessary blood transfusion*					
Jackson 2000[42]	Patients undergoing TJA, average age 65 years, range 20 to 80 years	Not stated	Lifetime	3% and 5%	USD, not reported
Ramkumar 2018[43]	65-year-old having TJA for severe osteoarthritits	Medical costs	10 years	3%	
Sonnenberg 2002[44]	65-year-old of composite sex and race undergoing THA	Not stated	Lifetime	3%	USD, 2000
**Intraoperative:** *Local infiltration of analgesia*					
Marques 2015[45]	Patients in trial[51] having THA or TKA, mean ages 66–69.5 years, 52%–65% female	Health and social care payer	1 year	None	GBP, not reported
**Intraoperative:** *Infection prevention*					
Cummins 2009[46]	68-year-olds of average health for their age undergoing THA due to degenerative arthritis	Hospital	Lifetime	3%	USD, 2002
Graves 2016[47]	Simulated cohort of patients who had THA in 2012	UK NHS	Lifetime	3%	GBP, 2012
Merollini 2013[48]	Not described	Healthcare system	30 years	3%	AUD, 2011
Nherera 2017[49]	Adults having routine TJA	UK NHS	6 weeks	None	GBP, 2015
**Postoperative:** *Physical therapy*					
Fusco & Turchetti, 2016[50]	Average age of 60 years, 44% males, 19% experienced complications during or after surgery	Societal & healthcare service	Lifetime	3%	Euro, 2013
Smith 2018[52]	Patients having unilateral TKA for knee osteoarthritis	Healthcare sector	Lifetime	3%	USD, 2016
Kauppila 2011[51]	60–80 year olds having unilateral TKR for knee osteoarthritis	Healthcare system	1 year	None	Euro, 2006

Supplemental Material: Enhanced recovery following hip and knee arthroplasty a systematic review of cost-effectiveness evidence. BMJ Open

**Postoperative:** *Scheduling of follow-up*					
Bolz 2010[53]	THA patients, mean age 69.9 years	Health services	7 years	3%	AUD, not reported
NHS: National Health Service; THA: total hip arthroplasty; TJA: total joint arthroplasty; TKA: total knee arthroplasty; UKA: unicompartmental knee arthroplasty; AUD: Australian dollar; DKK: Danish krone; GBP: British pound; USD: US dollar					

Supplemental Material: Enhanced recovery following hip and knee arthroplasty a systematic review of cost-effectiveness evidence. BMJ Open

Table A4. Costs included in each study

	Direct medical	Direct treatment	In-patient	Out-patient	Day care	Community healthcare	Medication	Side effect costs	Staff	Labs/diagnostic	Capital equipment	Social care	Travel costs	Productivity losses	Income forgone due to illness
Brunenberg 2005[37]	+	+	+	+		+	+		+			+		+	+
Larsen 2009[38]	+	+	+	+		+	+		+					+	+
McLawhorn 2016[39]	+	+	+					+							
Fernandes 2017[40]	+	+							+				+		+
Courville 2012[41]	+	+	+				+			+					
Jackson 2000[42]	+	+	+	+			+		+	+					
Ramkumar 2018[43]	+	+					+								
Sonnenberg 2002[44]	+	+	+	+			+								
Marques 2015[45]	+	+	+	+	+	+	+		+			+			
Cummins 2009[46]	+	+	+												
Graves 2016[47]	+	+	+				+				+				
Merollini 2013[48]	+	+	+				+		+		+				
Nherera 2017[49]	+	+	+	+		+	+								
Fusco & Turchetti, 2016[50]	+	+	+	+					+		+		+	+	+
Kauppila 2011[51]	+	+	+	+	+	+						+			
Smith 2018[52]	+	+					+	+							
Bolz 2010[53]	+		+	+						+					

Supplemental Material: Enhanced recovery following hip and knee arthroplasty a systematic review of cost-effectiveness evidence. BMJ Open

Table A5. Populations and tools used for eliciting utilities.

Authors	EQ-5D	15 D	QWB	SG	VAS / rating scale	TTO	AQoL	Other
Studies eliciting utilities								
Brunenberg 2005[37]	Patients in trial (UK tariff)	—	—	—	—	—	—	—
Larsen 2009[38,54]	Patients in trial (Danish tariff)	—	—	—	—	—	—	—
Fernandes 2017[40]	Patients in trial (Danish tariff)	—	—	—	—	—	—	—
Marques 2015[45]	Patients in trial (UK tariff)	—	—	—	—	—	—	—
Fusco & Turchetti, 2016[50]	TKR patients in trial (UK tariff)	—	—	—	—	—	—	—
Smith 2018[52]	Patients in trial (tariff not reported)	—	—	—	—	—	—	—
Kauppila 2011[51]	—	Patients in trial (Finnish tariff)	—	—	—	—	—	—
Studies using published utilities[*]								
McLawhorn 2016[39]	TKR patients in a trial; patients having bariatric surgery	—	—	—	Patients having bariatric surgery	—	—	—
Courville 2012[41]	—	—	Population reporting arthritis	—	—	—	—	—
Jackson 2000[42]	—	—	People with HIV infection	People with HIV infection	People with HIV infection	People with HIV infection	—	—
Ramkumar 2018[43]	—	—	—	—	—	—	—	Several sources
Sonnenberg 2002[45]	—	—	People with HIV infection	—	—	—	—	—
Cummins 2009[46]	—	—	—	—	—	THA patients	—	—

Supplemental Material: Enhanced recovery following hip and knee arthroplasty a systematic review of cost-effectiveness evidence. BMJ Open

Authors	EQ-5D	15 D	QWB	SG	VAS / rating scale	TTO	AQoL	Other
Graves 2016[47]	THA patients	THA patients	—	—	—	—	THA patients with prosthesis infection	—
Merollini 2013[48]	—	THA patients	—	—	—	—	THA patients with prosthesis infection	—
Nherera 2017[49]	—	—	—	—	—	—	—	SF-36 in patients undergoing orthopaedic surgery
Bolz 2010[53]	—	THA patients	—	—	—	—	—	—

* Details of utilities obtained from expert opinion are not included in this table
15-D, 15-dimension instrument; AQoL, Assessment of Quality of Life; EQ-5D, EuroQoL five-dimension instrument; HIV, human immunodeficiency virus; SF-36, Short Form (36) Health Survey; SG, standard gamble; THA, total hip arthroplasty; TKA, total knee arthroplasty; TTO, time trade-off; QWB, Quality of Well-being scale; VAS, visual analogue scale

Pritchard MG, *et al. BMJ Open* 2020; 10:e032204. doi: 10.1136/bmjopen-2019-032204

Supplemental Material: Enhanced recovery following hip and knee arthroplasty a systematic review of cost-effectiveness evidence. BMJ Open

Table A6. Overview of quality and sources of data used by studies included in this analysis

	Sources of data											
	Clinical effect sizes		Baseline clinical data		Duration of primary effect		Resource use		Costs		Utilities	
Authors	Min	Max	Min	Max	Min	Max	Min	Max	Min	Max	Min	Max
*Range of possible scores**	*1*	*9*	*1*	*6*	*1*	*6*	*1*	*6*	*1*	*6*	*1*	*4*
Brunenberg 2005[37]	—†	—†	—†	—†	—†	—†	—†	—†	1	2	1	1
Larsen 2009[38]	—†	—†	—†	—†	—†	—†	—†	—†	1	4	1	1
McLawhorn 2016[39]	1	8	2	2	NR	NR	NR	NR	2	6	2	4
Fernandes 2017[40]	—†	—†	—†	—†	—†	—†	—†	—†	1	1	1	1
Courville 2012[41]	1	1	1	1	NR	NR	2	2	2	2	2	2
Jackson 2000[42]	8	8	2	2	NR	NR	NR	NR	4	4	2	2
Ramkumar 2018[43]	1	8	4	4	6	6	4	4	4	4	1	4‡
Sonnenberg 2002[44]	8	8	2	2	NR	NR	3	3	1	3	2	4
Marques 2015[45]	—†	—†	—†	—†	—†	—†	—†	—†	1	2	1	1
Cummins 2009[46]	8	8	3	3	NR	NR	2	2	2	4	2	2
Graves 2016[47]	1	7	1	4	NR	NR	2	6	1	1	2	4
Merollini 2013[48]	1	7	1	3	NR	NR	NR	NR	1	6	2	4
Nherera 2017[49]	2	2	4	4	NA‖	NA‖	2	2	2	2	2	2
Fusco & Turchetti, 2016[50]	3	3	2	5	3	3	2	2	2	6	2	2
Kauppila 2011[51]	—†	—†	—†	—†	—†	—†	—†	—†	1	2	1	1
Smith 2018[52]	2	2	4	4	6	6	2	2	2	2	1	1
Bolz 2010[53]	NR	NR	2	2	NR	NR	NR	NR	2	2	2	2

Note: Where a study used several data sources within each category the results are reported as a range of the minimum to maximum data quality score within each category.
* Details of the full hierarchy are available in supplementary table A2 online.
† Not applicable as study is not model-based
‡ Utilities from patients with other conditions
‖ Model time horizon limited to duration of study providing clinical effect sizes

CHEC, Consensus on Health Economic Criteria; ISPOR, International Society for Pharmacoeconomics and Outcomes Research; NA, not applicable; NR, not reported

Pritchard MG, *et al. BMJ Open* 2020; 10:e032204. doi: 10.1136/bmjopen-2019-032204

Supplemental Material: Enhanced recovery following hip and knee arthroplasty a systematic review of cost-effectiveness evidence. BMJ Open

Table A7. Summary of findings from studies included in this analysis

Authors, country	Population	Strategy	Cost	Outcome in QALYs	ICER in cost per QALY*	Probability of cost-effectiveness (threshold in cost per QALY)
**Enhanced care pathway**						
Brunenberg, *et al.*[37] Netherlands	THA	Conventional care	USD 11 312	0.65		
		Joint Recovery Programme (pre- assessment and intensive rehabilitation)	USD 10 051	0.70		
		Increment	−1261	0.07†	Dominates	0.94 (USD 45 000)
	TKA	Conventional care	USD 12 877	0.61		
		Joint Recovery Programme (pre-operative assessment and intensive rehabilitation)	USD 8541	0.65		
		Increment	−3336	0.04†	Dominates	0.99 (USD 45 000)
Larsen, *et al.*,[38] Denmark	THA + TKA	Conventional care	DKK 90 227	0.78		
		Accelerated perioperative care and rehabilitation	DKK 71 344	0.83		
		Increment	−18 880	0.05	Dominates	0.97‡ (DKK 160 000)
	THA	Conventional care	DKK 87 657	0.75		
		Accelerated perioperative care and rehabilitation	DKK 71 768	0.84		
		Increment	−15 889	0.09	Dominates	0.98‡ (DKK 160 000)
	TKA	Accelerated perioperative care and rehabilitation	DKK 70 644	0.81		
		Conventional care	DKK 95 367	0.85		
		Increment	24 723	0.04	DKK 618 075	NR

Supplemental Material: Enhanced recovery following hip and knee arthroplasty a systematic review of cost-effectiveness evidence. BMJ Open

Table A7. Continued.

Authors, country	Population	Strategy	Cost	Outcome in QALYs	ICER in cost per QALY*	Probability of cost-effectiveness (threshold in cost per QALY)
**Preoperative**						
Assessment and optimization of comorbidities						
McLawhorn, *et al.*,[39] US	Morbidly obese with unilateral end-stage knee osteoarthritis					
		Immediate TKA	USD 60 453	10.83		
		Bariatric surgery, followed by TKA 2 years later	USD 84 099	12.53		
		Increment	USD 23 646	1.70	USD 13 910	0.988 (USD 100 000)
Exercise						
Fernandes, *et al.*[40] Denmark	THA + TKA	Educational package	EUR 16 313	0.61		
		Supervised neuromuscular exercise + educational package	EUR 16 181	0.66		
		Increment	EUR −132	0.04†	Dominates	0.84 (EUR 40 000)
Staphylococcus aureus *prophylaxis*						
Courville, *et al.*,[41] US	THA	Standard infection prevention measures without *S. aureus* screening or mupirocin decolonization	USD 24 506	0.7980		
		Preoperative nasal screening for *S. aureus* followed by mupirocin treatment for patients with positive cultures	USD 24 471	0.7983	D	
		Empirical treatment of all preoperative patients with mupirocin	USD 24 258	0.7985		
		Increment (compared to standard measures)	−248	0.0005	Dominates	NR
	TKA	Standard infection prevention measures without *S. aureus* screening or mupirocin decolonization	USD 24 667	0.6783		
		Preoperative nasal screening for *S. aureus* followed by mupirocin treatment for patients with positive cultures	USD 24 611	0.6785	D	
		Empirical treatment of all preoperative patients with mupirocin	USD 24 378	0.6787		
		Increment (compared to standard measures)	−289	0.0004	Dominates	NR

Supplemental Material: Enhanced recovery following hip and knee arthroplasty a systematic review of cost-effectiveness evidence. BMJ Open

Authors, country	Population	Strategy	Cost	Outcome in QALYs	ICER in cost per QALY*	Probability of cost-effectiveness (threshold in cost per QALY)
**Intraoperative**						
Avoid unnecessary blood transfusion						
Jackson, *et al.*,[42] US	THA + TKA	Usual transfusion practice	NR	NR		
		Postoperative erythrocyte recovery and transfusion	NR	NR		
		Increment	USD 53	0.00001	USD 5 700 000	NR (USD 50 000)
Ramkumar, *et al.*,[43] US	THA + TKA	No pharmacologic haemostatic agent	USD 951	8.893		
		Single dose intravenous aminocaproic acid	USD 1175	9.152	D	
		Single dose intravenous tranexamic acid	USD 460	9.339		
		Increment (compared to no pharmacologic haemostatic agent)	USD −491	0.447	Dominates	NR (USD 100 000)
Sonnenberg,[44] US	THA	Usual practice without autologous donation	USD 1395	NR		
		Autologous blood donation and transfusion	USD 1539	NR		
		Increment	144	0.0523	USD 2750	NR (USD 50 000)
Local infiltration of analgesia						
Marques, *et al.*,[45] UK	THA	Standard anaesthesia	NR	NR		
		Intraoperative local anaesthetic wound infiltration administered before wound closure in addition to standard anaesthesia	NR	NR		
		Increment	GBP −86	0.052	Dominates	0.98 (GBP 20 000)
	TKA	Standard anaesthesia	NR	NR		
		Intraoperative local anaesthetic wound infiltration administered before wound closure in addition to standard anaesthesia	NR	NR		
		Increment	GBP −77	0.009	Dominates	0.60 (GBP 20 000)
Infection prevention						
Cummins, *et al.*,[46] US	THA	Conventional cement	USD 24 100	9.439		
		Antibiotic-impregnated bone cement	USD 23 900	9.454		
		Increment	−200	0.015	Dominates	NR

Pritchard MG, *et al. BMJ Open* 2020; 10:e032204. doi: 10.1136/bmjopen-2019-032204

Supplemental Material: Enhanced recovery following hip and knee arthroplasty a systematic review of cost-effectiveness evidence. BMJ Open

Authors, country	Population	Strategy	Cost	Outcome in QALYs	ICER in cost per QALY*	Probability of cost-effectiveness (threshold in cost per QALY)
Graves, *et al.*,[47] UK[§]	THA	No systemic antibiotics, plain cement and conventional ventilation	GBP 0[§]	0[§]		
		Systemic antibiotics, antibiotic-impregnated cement, laminar ventilation and body exhaust suit	GBP 781 075	62	D	0.01[‖] (GBP 18 000)
		No systemic antibiotics, antibiotic-impregnated cement and conventional ventilation	GBP −4 634 647	89	D	0.07[‖] (GBP 18 000)
		Systemic antibiotics, plain cement and conventional ventilation	GBP −7 226 732	101	D	0.15[‖] (GBP 18 000)
		Systemic antibiotics, antibiotic-impregnated cement, conventional ventilation and body exhaust suit	GBP −3 960 897	106	D	0.11[‖] (GBP 18 000)
		Systemic antibiotics, plain cement and laminar airflow	GBP −5 271 040	118	D	0.10[‖] (GBP 18 000)
		Systemic antibiotics, antibiotic-impregnated cement and laminar airflow	GBP −6 152 877	124	D	0.06[‖] (GBP 18 000)
		No systemic antibiotics, plain cement and laminar airflow	GBP −3 271 749	124	D	0.18[‖] (GBP 18 000)
		Systemic antibiotics, antibiotic-impregnated cement and conventional ventilation	GBP −8 325 277	147		
		Increment (compared to systemic antibiotics, plain cement and conventional ventilation)	−1 098 545	46	Dominates	0.32[e] (GBP 18 000)
Merollini, *et al.*,[48] Australia	THA	No antibiotic prophylaxis	AUD 1 517 954	−163.1		
		Antibiotic prophylaxis and laminar airflow	AUD 4 592 200	−126.9	D	
		Antibiotic prophylaxis	AUD 0[¶]	0[¶]	D	
		Antibiotic prophylaxis and antibiotic-impregnated cement	AUD −126 375	32.3		
		Increment	−126 375	32.3	Dominates	0.986 (AUD 40 000)[**]
Nherera, *et al.*,[49] UK	THA + TKA	Usual care	GBP 6713	0.115		
		Single-use negative pressure wound therapy dressings	GBP 5602	0.116		
		Increment	GBP −1049	0.0014	Dominates	0.91 (GBP 20 000)

Supplemental Material: Enhanced recovery following hip and knee arthroplasty a systematic review of cost-effectiveness evidence. BMJ Open

Table A7. Continued.

Authors, country	Population	Strategy	Cost	Outcome in QALYs	ICER in cost per QALY*	Probability of cost-effectiveness (threshold in cost per QALY)
**Postoperative**						
Physical therapy						
Fusco & Turchetti,[50] Italy	TKA, societal perspective	20 face-to-face rehabilitation sessions	EUR 1315	13.02		
		10 face-to-face rehabilitation sessions plus 10 telesessions	EUR 977	13.02		
		Increment	−338	0	Dominates	NR (EUR 30 000)
	TKA, Italian NHS perspective	20 face-to-face rehabilitation sessions	EUR 1124	13.02		
		10 face-to-face rehabilitation sessions plus 10 telesessions	EUR 862	13.02		
		Increment	−262	0	Dominates	NR (EUR 30 000)
Kauppila, *et al.*,[51] Finland	TKA	Multidisciplinary biopsychosocial outpatient rehabilitation programme	EUR 12 950	NR		
		Conventional orthopaedic care	EUR 11 120	NR		
		Increment	−1830	0.0192	Dominates	NR
Smith, *et al.*,[52] US		Telephone calls conveying general health messages	USD 140 700	9.783		
		Telephonic health coaching and financial incentives	USD 141 000	9.788		
		Increment	USD 300	0.005	USD 57 200	0.70 (USD 100 000)
Scheduling of follow-up						
Bolz, *et al.*,[53] Australia[††]	THA, assuming 5% revisions delayed under no follow-up[‡‡]					
		2-yearly routine follow-up	AUD 26 426 908	147 940		
		Follow-up at 3 months and 1 or 2 years	AUD 21 331 518	147 940	D	
		No follow-up	AUD 14 867 616	147 949		
		Increment	−6 463 902	9	Dominates	NR

* Strategies listed in order of increasing effectiveness; ICER presented compared to next most effective strategy that is not extended-dominated; no ICER presented for dominated or extended-dominated strategies; ICERs have been calculated where not explicitly presented in the reference, where ICERs have been presented they may differ slightly from what would be calculated from the presented figures due to rounding.
† Adjusted for baseline quality of life using regression analysis
‡ Probability of being the dominant strategy

Supplemental Material: Enhanced recovery following hip and knee arthroplasty a systematic review of cost-effectiveness evidence. BMJ Open

§ Total costs and outcomes for a cohort of 77 321 patients, relative to the cost of the strategy of no systemic antibiotics, plain cement and conventional ventilation
‖ Probability of being most cost-effective strategy in the model
¶ Total costs and outcomes per 30 000 primary THRs, relative to the cost of the strategy of antibiotic prophylaxis
** Cost-effectiveness ≥98.6% at typical willingness-to-pay values of AUD 40 000 to AUD 64 000
†† Total costs and outcomes for a cohort of 30 440 patients
‡‡ Four different probabilities were modelled (1%, 5%, 10%, 50%), but the direction of the results did not change between them

AUD, Australian dollar; D, dominated; DKK, Danish krone; EUR, euro; GBP, British pound; ICER, incremental cost-effectiveness ratio; NHS, National Health Service; NR, not reported; QALY, quality-adjusted life year; THA, total hip arthroplasty; TKA, total knee arthroplasty; UK, United Kingdom; US, United States; USD, United States dollar

Supplemental Material: Enhanced recovery following hip and knee arthroplasty a systematic review of cost-effectiveness evidence. BMJ Open

Table A8. Components of enhanced recovery pathway in the trials of an entire pathway

	**Larsen, *et al.*[38]**		**Brunenberg, *et al.*[37]**	
**Intervention type**	**Standard protocol**	**Accelerated protocol**	**Usual care**	**Joint recovery programme**
Pre-operative assessment	NA	NA	No standardized screening	History, examination & blood testing 6 weeks prior to surgery
Pre-operative education	Information given to patients on admission	Information provided in groups at outpatient clinic prior to hospitalization	No information session	Information session 1–2 weeks prior to surgery
Day of admission	Day before surgery	Day of surgery	NA	NA
Bed allocation in hospital	Amongst other patients	Patients having joint replacement placed together in separate part of ward	NA	NA
Staff involved	Various	One nurse in charge of multidisciplinary team of nurses, OT & PT	NA	Supervised by PT & nurses throughout admission
Nutrition	Screening	Screening plus daily intake 1.5 L fluid & 2 protein drinks	NA	NA
Start of mobilization	1 day after surgery	Day of surgery	NA	NA
Description of mobilization	Individual & gradual mobilization according to patient tolerance	Intensive mobilization in teams with pre-set goals	Conventional physiotherapy	Rehabilitation in groups in a room resembling the home situation
Duration of mobilization	4 hours daily	8 hours daily	1 hour daily	Duration not specified
Discharge planning	NA	NA	During admission	6 weeks prior to surgery
Other details	NA	NA		Patients could involve a family member or friend to give emotional support

NA, not applicable; OT, occupational therapists; PT, physiotherapists

Supplemental Material: Enhanced recovery following hip and knee arthroplasty a systematic review of cost-effectiveness evidence. BMJ Open

Table A9. Willingness to pay thresholds and incremental cost-effectiveness ratios for all studies converted into international dollars

Authors, country	Currency, cost year	Values as reported*		Adjusted values in international dollars†	
		**WTP threshold**	**ICER (unit cost/QALY)**	**WTP threshold**	**ICER (unit cost/QALY)**
Brunenberg, *et al.*[37] Netherlands	USD, 2002	45 000	—‡	45 000	—‡
Larsen, *et al.*,[38] Denmark	DKK, 2006	160 000	618 075	22 122	99 624
McLawhorn, *et al.*,[39] US	USD, 2012	100 000	13 910	100 000	14 995
Fernandes, *et al.*[40] Denmark	EUR, 2012	40 000	—‡	41 720	—‡
Courville, *et al.*,[41] US	USD, 2005	NR	—‡	—	—‡
Jackson, *et al.*,[42] US	USD, 2000§	50 000	5 700 000	50 000	7 787 912
Ramkumar, *et al.*,[43] US	USD, 2016	100 000	—‡	100 000	—‡
Sonnenberg,[44] US	USD, 2000	50 000	2 750	50 000	3 757
Marques, *et al.*,[45] UK	GBP, 2015§	20 000	—‡	28 838	—‡
Cummins, *et al.*,[46] US	USD, 2002	NR	—‡	—	—‡
Graves, *et al.*,[47] UK	GBP, 2012	18 000	—‡	25 954	—‡
Merollini, *et al.*[48], Australia	AUD, 2011	40 000	—‡	27 287	—‡
Nherera, *et al.*,[49] UK	GBP, 2015	20 000	—‡	28 838	—‡
Fusco & Turchetti,[50] Italy	EUR, 2013	30 000	—‡	41 482	—‡
Kauppila, *et al.*,[51] Finland	EUR, 2006	NR	—‡	—	—‡
Smith, *et al.*,[52] US	USD, 2016	100 000	57 200	100 000	57 200
Bolz, *et al.*,[53] Australia	AUS, 2010§	NR	—‡	—	—‡

* Only non-dominating results reported
† Willingness to pay thresholds not adjusted for cost year as they have been constant in most countries since they were introduced, in spite of cost inflation; ICERs additionally adjusted to 2016 values
‡ Dominance reported for all strategies
§ Cost year not reported; assumed as publication year

AUD, Australian dollar; DKK, Danish krone; EUR, euro; GBP, British pound; ICER, incremental cost-effectiveness ratio; NR, not reported; QALY, quality-adjusted life year; UK, United Kingdom; US, United States; USD, United States dollar; WTP, willingness to pay

Pritchard MG, *et al. BMJ Open* 2020; 10:e032204. doi: 10.1136/bmjopen-2019-032204
