# Supplementary material for: Enhanced recovery following hip and knee arthroplasty: a systematic review of cost-effectiveness evidence
Source: BMJ Open. 2020 Jan 15;10(1):e032204. doi: 10.1136/bmjopen-2019-032204 (PMC7044879; doi:10.1136/bmjopen-2019-032204)
Supplement: Author's manuscript [file bmjopen-2019-032204.draft_revisions.pdf]

BMJ Open

BMJ Open is committed to open peer review. As part of this commitment we make the peer review history of every article we publish publicly available.

When an article is published we post the peer reviewers' comments and the authors' responses online. We also post the versions of the paper that were used during peer review. These are the versions that the peer review comments apply to.

The versions of the paper that follow are the versions that were submitted during the peer review process. They are not the versions of record or the final published versions. They should not be cited or distributed as the published version of this manuscript.

BMJ Open is an open access journal and the full, final, typeset and author-corrected version of record of the manuscript is available on our site with no access controls, subscription charges or pay-per-view fees (http://bmjopen.bmj.com).

If you have any questions on BMJ Open's open peer review process please email
info.bmjopen@bmj.com

BMJ Open

Enhanced recovery following hip and knee arthroplasty: A systematic review of cost-effectiveness evidence

Journal:	*BMJ Open*
Manuscript ID	bmjopen-2019-032204
Article Type:	Research
Date Submitted by the Author:	07-Jun-2019
Complete List of Authors:	Pritchard, Mark; University of Oxford, Nuffield Department of Population Health<br>Murphy, Jacqueline; Queen Mary University of London,<br>Cheng, Lok; University of Oxford, Nuffield Department of Population Health<br>Janarthanan, Roshni; University of Oxford, Nuffield Department of Population Health<br>Judge, Andrew; University of Bristol, Musculoskeletal Research Unit, Translational Health Sciences, Bristol Medical School, University of Bristol, Learning and Research Building, Level 1, Southmead Hospital, Southmead, BS10 5NB<br>Leal, Jose; University of Oxford, Nuffield Department of Population Health
Keywords:	Systematic review, hip replacement, knee replacement, osteoarthritis, economic evaluation, cost-effectiveness

SCHOLARONE™
Manuscripts

Enhanced recovery following hip and knee arthroplasty: A systematic review of cost-effectiveness evidence

Mark G Pritchard, MB ChB, MSc,[1,2,*] mark.pritchard@ndm.ox.ac.uk, ORCID iD 0000-0003-1726-8989

Jacqueline Murphy, MMath,[1,*] jacqueline.murphy@qmul.ac.uk, ORCID iD 0000-0002-3927-7002

Lok Yin Cheng, MBChB, BSc,[1,4] lok.cheng@nhs.net

Roshni Janarthanan MBBS,[1,2] roshni.janarthanan@medsci.ox.ac.uk

Andrew Judge, PhD,[5,6] andrew.judge@bristol.ac.uk

José Leal, DPhil,[1] jose.leal@dph.ox.ac.uk, ORCID iD 0000-0001-7870-6730

1 Health Economics Research Centre, Nuffield Department of Population Health, University of Oxford, Oxford, United Kingdom

2 Oxford University Hospitals NHS Foundation Trust, John Radcliffe Hospital, Oxford, United Kingdom

3 Wolfson Institute of Preventive Medicine, Queen Mary University London

4 Stoke Mandeville Hospital, Buckinghamshire Healthcare NHS Trust, Aylesbury, Buckinghamshire, United Kingdom

5 Nuffield Department of Orthopaedics, Rheumatology and Musculoskeletal Sciences (NDORMS), Centre for Statistics in Medicine, University of Oxford, Oxford, United Kingdom

6 Musculoskeletal Research Unit, Translational Health Sciences, Bristol Medical School, University of Bristol, Bristol, United Kingdom

* JM and MGP contributed equally to this study.

Correspondence to: Dr José Leal, Health Economics Research Centre, Nuffield Department of Population Health, University of Oxford, Old Road Campus, Oxford OX3 7LF, United Kingdom.

Telephone: 01865 289 263. jose.leal@dph.ox.ac.uk

Word count: 3495

For peer review only

Abstract

Objectives: To assess evidence from cost–utility analyses of enhanced recovery pathways for patients having total hip arthroplasty (THA) or total knee arthroplasty for osteoarthritis, report on study quality, and identify research gaps for future work.

Design: Systematic review.

Methods: Systematic searches of Medline, Embase, National Health Service Economic Evaluations Database and Econlit were carried out to identify peer-reviewed papers between 2000 and 2017, describing cost–utility analyses of enhanced recovery pathways, or components of one, compared to usual care, in patients having THA or TKA. Reference lists were also reviewed.

Results: We identified two cost–utility analyses of entire enhanced recovery pathways and 11 analyses of pathway components. Four were trial-based and the remainder were model-based. We had concerns about the risk of bias for all included studies, particularly regarding the short time horizon of the trials and lack of reporting of model validation.

Conclusions: Consistent results were found supporting enhanced recovery pathways as a whole, prophylactic systemic antibiotics, antibiotic-impregnated cement and conventional ventilation for infection prevention. There is scope for future cost-effectiveness studies, in particular analyses of entire recovery pathways and comparison of incremental changes within pathways rather than considering aspects of care in isolation.

Registration: *International Prospective Register of Systematic Reviews* number CRD42017059473

Keywords: Systematic review, hip replacement, hip arthroplasty, knee replacement, knee arthroplasty, osteoarthritis, economic evaluation, cost-effectiveness

For peer review only - http://bmjopen.bmj.com/site/about/guidelines.xhtml

ARTICLE SUMMARY

Strengths and limitations of this study

- This systematic review of enhanced recovery pathways for hip and knee arthroplasties had a detailed search strategy, including entire pathways and their components.
- Appropriate tools were used to assess quality and validity of models, trials and economic evaluations.
- Conclusions were reliant on the availability, quality and validity of published studies into cost-effectiveness of hip and knee arthroplasty.

For peer review only

For peer review only - http://bmjopen.bmj.com/site/about/guidelines.xhtml

Funding statement

This project was funded by the National Institute for Health Research Health Services and Delivery Research Programme (project number 14/46/02). Support was received from the Oxford NIHR Biomedical Research Centre, Nuffield Orthopaedic Centre, University of Oxford. AJ was supported by the NIHR Biomedical Research Centre at the University Hospitals Bristol NHS Foundation Trust and the University of Bristol. The views and opinions expressed herein are those of the authors and do not necessarily reflect those of the HS&DR Programme, NIHR, NHS or the Department of Health and Social Care.

Competing interests statement

The authors declare they have no competing interests.

For peer review only

INTRODUCTION

Hip and knee arthroplasties are common procedures: around a million of each were performed in 2011 within Organisation for Economic Co-operation and Development (OECD) countries,[1 2] and rates continue to increase.[3-6] Enhanced recovery is a multimodal approach to reduce surgical morbidity and mortality. Recognizing that factors other than surgical technique affect patient outcomes, Kehlet[7] considered how to optimize the preoperative, intraoperative and postoperative phases of patient care. These principles have been further developed specifically within the context of hip and knee arthroplasty.[8-14] A recent systematic review[15] found that enhanced recovery after hip and knee arthroplasties reduced length of stay in hospital. The authors cited a study from New Zealand[16] that found an enhanced recovery pathway to be cost saving, but the study did not include any data on cost-effectiveness.

Cost–utility analyses have become the preferred approach to inform decisions on healthcare resource allocation.[17 18] In these, the effects of treatments are measured in quality-adjusted life years (QALY): the product of health-related quality of life (anchored at 0 for death and 1 for perfect health), and the time (in years) spent experiencing that level of health. Incremental cost-effectiveness ratios (ICER) are used to compare a treatment to a less effective alternative and a threshold value used to determine whether it is cost-effective. Different countries have different thresholds for how much they are willing to pay per QALY gained.[19]

Systematic reviews of cost-effectiveness analyses have considered arthroplasty *vs* conservative management,[20 21] and specific components of enhanced recovery such as thromboprophylaxis.[22-24] However, we are not aware of any systematic reviews investigating the cost-effectiveness of a complete enhanced recovery pathway, or of most of the components. Our aim was to assess the cost-effectiveness evidence of enhanced recovery for patients having hip and knee arthroplasty. Specifically, we were interested in studies of adults having total hip arthroplasty (THA) or total knee arthroplasty (TKA) for osteoarthritis, comparing an enhanced recovery pathway or components of one against usual care. Secondarily, we wanted to report study quality and

For peer review only - http://bmjopen.bmj.com/site/about/guidelines.xhtml

identify research gaps for future work.

METHODS

Common components of an enhanced recovery pathway after hip and knee arthroplasty are listed in Box 1. The complete methods of the review are available in the published protocol,[25] registered with the International Prospective Register of Systematic Reviews, number CRD42017059473.[26] The selection of electronic databases and the search strategy were developed with an information specialist and in line with the preferred reporting items for systematic reviews and meta-analyses (PRISMA).[27] Briefly, we searched Ovid MEDLINE, Embase, the National Health Service Economic Evaluations Database (via the Cochrane Library), and EconLit (via ProQuest), for English-language peer-reviewed papers published between 1 January 2000 and 1 March 2017 that included a cost–utility analysis of an enhanced recovery pathway or components of one, compared to usual care in patients having hip or knee arthroplasties (the complete search strategy for each database is presented in the Supplementary Table A1). Additional publications meeting our inclusion criteria were identified from the reference lists of the included studies. Our target population was adults having surgery for osteoarthritis. Studies exclusively concerning populations with other indications for surgery were excluded. We included studies with patients having different indications for surgery if the majority had osteoarthritis, or if the presented results allowed independent extraction of data for the subpopulation with osteoarthritis. Given that osteoarthritis is an indication for 92% of hip and 96% of knee arthroplasties,[28] we assumed that studies not providing details of the indication for surgery were representative of a population with osteoarthritis, and therefore included these studies. Evaluations of surgical technique or choice of implant were excluded.

Studies were independently screened based on their titles and abstracts by three reviewers (JM, LYC and JL). Full texts were obtained for studies chosen for inclusion by any reviewer. As an amendment to the protocol, evaluations of thromboprophylaxis were excluded at the full-text stage due to a recent comprehensive systematic review in that area.[22] Data extraction was performed for

For peer review only - http://bmjopen.bmj.com/site/about/guidelines.xhtml

remaining studies by three reviewers (JM, MGP and RJ), with disagreements resolved by a fourth reviewer (JL). The data extraction proforma is included in the published protocol.[25]

To assess the quality of studies, we completed the Consensus on Health Economic Criteria (CHEC) list[29] for each publication. For model-based studies, we also used the questionnaire produced by the International Society for Pharmacoeconomics and Outcomes Research (ISPOR),[30] and the Assessment of the Validation Status of Health-Economic (AdViSHE) decision models tool.[31] We added the Cochrane Collaboration's tool[32] to the original protocol to assess risk of bias in trial-based studies, referring to the original reports of trial outcomes where necessary. We assessed the quality of the data sources used in the studies according to a pre-specified hierarchy of evidence tool (see Supplementary Table A2).[33 34] For each component of a study, a score of 1 represented the use of the most appropriate data source, with increasing numbers representing progressively less appropriate sources.

The principal outcomes were a point estimate of cost-effectiveness in terms of incremental cost per QALY gained, and the probability of an intervention being cost effective according to the willingness to pay threshold used by the authors of each study. We also examined whether an intervention could be cost-effective in a different country or setting than the original study. Hence, we assumed the studies to be generalizable and the ICERs to be comparable after conversion into 2016 purchasing power parity international dollars (US PPP).[35] As the studies had different methodologies and looked at different combinations of components of the enhanced recovery pathway, no quantitative synthesis of the study results was attempted.

For peer review only - http://bmjopen.bmj.com/site/about/guidelines.xhtml

Box 1. Suggested components of an enhanced recovery pathway for hip and knee arthroplasty patients, data from references[8-14].

Preoperative
- Education
- Discharge planning
- Multidisciplinary assessment
- Optimization of comorbidities
- Premedication (possibly including standardized analgesia and steroids)
- Nutrition screening

Intraoperative
- Standardized intravenous fluids
- Avoid unnecessary blood transfusion
- Minimally invasive surgery
- Reduce heat loss
- Specified anaesthetic requirements, such as spinal anaesthesia
- Local infiltration of analgesia
- Tranexamic acid
- Prophylactic antibiotics
- Intravenous dexamethasone
- Avoid unnecessary drains

Postoperative
- Analgesia
  - Continuous neural block
  - Standardized multimodal analgesia, scheduled and as required
  - Reduce opioid use / avoid patient-controlled intravenous opioid analgesia
- Scheduled anti-emetic
- Physical therapy starting on day of surgery
- Avoid unnecessary blood transfusion
- Oxygen administration
- Avoid sleep disturbances
- Early oral nutrition
- Thromboprophylaxis
- Neuromuscular electrical stimulation
- Aim for early discharge

For peer review only

RESULTS

Study selection

Our search identified 11 060 publications, and one additional publication was found from other sources. After excluding duplicates, we screened 6221 titles and abstracts. We excluded 6072 papers based on their abstracts and 136 following review of their full texts (reasons for exclusion given in Figure 1; details of the studies excluded due to not reporting QALYs are given in Supplementary Table A3). We therefore included thirteen papers in this review.[36-48]

Overview of included studies

Table 1 summarises included studies. Five studies included both THA and TKA,[36 37 39 40 42] five only THA,[41 43-45 48] and three only TKA.[38 46 47] Two papers looked at an entire enhanced recovery pathway.[36 37] We identified cost–utility evaluations of optimization of comorbidities (specifically morbid obesity),[38] measures to reduce allogenic blood transfusion,[40 41] local infiltration of anaesthetic,[42] prophylactic antibiotics and other infection prevention measures,[39 43-45] and physical therapy.[46 47] The final study[48] concerned the optimal timing of follow-up which, although not included in our prior list of enhanced recovery interventions, we have included here as being allied to the pathway. Four studies were trial-based economic evaluations,[36 37 42 47] the remaining nine being model-based. Six studies were from Europe,[36 37 42 44 46 47] five from the United States,[38-41 43] and two from Australia.[45 48] Perspective, time horizon, discount rates and price year used in each study are reported in Supplementary Table A4. The study perspective varied from only hospital costs[40 41 43 45 48] to a broader (societal) cost perspective.[36-39 46] Types of costs captured in each study are reported in Supplementary Table A5. QALYs were informed by utility values based on the EQ-5D-3L instrument in three trial-based studies[36 42 49] and on the 15D instrument in one trial-based study (Supplementary Table A6).[47 50]

For peer review only - http://bmjopen.bmj.com/site/about/guidelines.xhtml

Table 1. Summary of studies included in this analysis

Authors, publication year	Comparison	Joint	Study type	Country
**Enhanced recovery pathway**				
Brunenberg, *et al.* 2005[36]	Joint Recovery Programme (pre-assessment and intensive rehabilitation), *vs* conventional care	Hip and knee	Trial-based	Netherlands
Larsen, *et al.*, 2009[37]	Accelerated perioperative care and rehabilitation, *vs* conventional care	Hip and knee	Trial-based	Denmark
**Preoperative**				
McLawhorn, *et al.*, 2016[38]	Bariatric surgery followed by TKA 2 years later, *vs* immediate TKA	Knee	Markov model	US
Courville, *et al.*, 2012[39]	Preoperative nasal screening for *S. aureus* colonization followed by mupirocin treatment for patients with positive cultures, *vs* empirical treatment of all preoperative patients with mupirocin, *vs* standard infection prevention measures without *S. aureus* screening or mupirocin decolonization.	Hip and knee	Decision tree model	US
**Intraoperative**				
Jackson, *et al.*, 2000[40]	Postoperative erythrocyte recovery and transfusion, *vs* usual transfusion practice	Hip and knee	Markov model	US
Sonnenberg, 2002[41]	Autologous blood donation and transfusion, *vs* usual practice without autologous donation	Hip	Markov model	US
Marques, *et al.*, 2015[42]	Intraoperative local anaesthetic wound infiltration administered before wound closure in addition to standard anaesthesia, *vs* standard anaesthesia	Hip and knee	Trial-based	UK
Cummins, *et al.*, 2009[43]	Antibiotic-impregnated bone cement, *vs* conventional cement	Hip	Markov model	US
Graves, *et al.*, 2016[44]	Nine arms, comparing combinations of prophylactic systemic antibiotics, antibiotic-impregnated cement, laminar airflow and body exhaust suits	Hip	Markov model	UK
Merollini, *et al.*, 2013[45]	No antibiotic prophylaxis, antibiotic prophylaxis and antibiotic-impregnated cement, and antibiotic prophylaxis and laminar airflow, each compared to a baseline strategy of routine antibiotic prophylaxis	Hip	Markov model	Australia
**Postoperative**				
Fusco & Turchetti, 2016[46]	10 face-to-face rehabilitation sessions plus 10 telesessions, *vs* 20 face-to-face rehabilitation sessions	Knee	Markov model	Italy
Kauppila, *et al.*, 2011[47]	Multidisciplinary biopsychosocial outpatient rehabilitation programme, *vs* conventional orthopaedic care	Knee	Trial-based	Finland
Bolz, *et al.*, 2010[48]	2-yearly routine follow-up, *vs* follow-up at 3 months and 1 or 2 years, *vs* no follow-up	Hip	Markov model	Australia

NHS: National Health Service; THA: total hip arthroplasty; TJA: total joint arthroplasty; TKA: total knee arthroplasty; UKA: unicompartmental knee arthroplasty

For peer review only - http://bmjopen.bmj.com/site/about/guidelines.xhtml

Assessment of study and reporting quality

Using the CHEC list, the quality of the studies was generally good (Figure 2a). Items raising more concerns were the short time horizons of the four trial-based studies (one year)[36 37 42 47] and two model-based studies (one[39] and seven[48] years) (see Supplementary Table A4). Using the Cochrane Collaboration's tool (Figure 2b), the risk of bias of the trial-based studies was low for items such as incomplete outcome data and selective reporting but high or uncertain for the remainder. Three trial-based studies stated that participants were allocated at random,[49 51 52] the fourth was a before and after trial whereby patients were recruited consecutively from a waiting list.[36] Larsen and colleagues[49] discussed stratification, and Kauppila and colleagues[52] reported a computer-generated sequence. The staff administering the interventions were not blinded to allocation in any trial, and in three trials[36 51 52] outcomes were assessed by researchers aware of the treatment allocation. Using the ISPOR questionnaire, the quality of the model-based studies was generally good (Figure 2c). However, none of the model-based studies reported a detailed process for internal and external validation. Three studies[40 41 46] were based on previously published models.[53-55] Of these, only Briggs and colleagues[53] (the basis for Fusco and Turchetti's model[46]) provided details of model validation. Some model-based studies excluded potential important outcomes, for example, Cummins and colleagues[43] did not include the possibility of requiring more than one revision surgery, and in Bolz and colleagues' model[48] there was no reduction in utility associated with delayed revisions. Further limitations in model validation were highlighted with the AdViSHE tool (Figure 2d).[31]

The hierarchy of evidence used in the studies is reported in Supplementary Table A7. Three model-based studies[40 41 43] scored poorly as they did not use randomised trial data to inform the clinical effective size of the interventions being analysed. Most studies did not cite a source for the assumption of the duration of treatment effect beyond that observed in the primary source for clinical effect size. Four trial-based studies and one model-based study included assessments of utilities from patients using validated tools and scored highly in terms of quality of evidence.

For peer review only - http://bmjopen.bmj.com/site/about/guidelines.xhtml

Results of economic evaluation

The results of the included studies are summarized in Table 2. Costs, QALYs and incremental cost-effectiveness ratios for each comparison performed in each study are listed in Supplementary Table A8.

For peer review only

Table 2. Summary of findings from studies included in this analysis

Authors, country	Population	Strategy	Cost-effective?
**Enhanced recovery pathway**			
Brunenberg, *et al.*,[36] Netherlands	THA and TKA	Conventional care	-
		Joint Recovery Programme (pre- assessment and intensive rehabilitation)	Yes, more effective and less costly
Larsen, *et al.*,[37] Denmark	THA	Conventional care	-
		Accelerated perioperative care and rehabilitation	Yes, more effective and less costly
	TKA	Conventional care	-
		Accelerated perioperative care and rehabilitation	Yes, less effective but less costly
**Preoperative**			
McLawhorn, *et al.*,[38] US	Morbid obese TKA	Immediate TKA	-
		Bariatric surgery, followed by TKA 2 years later	Yes
Courville, *et al.*,[39] US	THA and TKA	Standard infection prevention measures without *S. aureus* screening or mupirocin decolonization OR preoperative nasal screening for *S. aureus* followed by mupirocin treatment for patients with positive cultures	-
		Empirical treatment of all preoperative patients with mupirocin	Yes, more effective and less costly
**Intraoperative**			
Jackson, *et al.*,[40] US	THA and TKA	Usual transfusion practice	-
		Postoperative erythrocyte recovery and transfusion	No
Sonnenberg,[41] US	THA	Usual practice without autologous donation	-
		Autologous blood donation and transfusion	Yes
Marques, *et al.*,[42] UK	THA and TKA	Standard anaesthesia	-
		Intraoperative local anaesthetic wound infiltration administered before wound closure in addition to standard anaesthesia	Yes, more effective and less costly
Cummins, *et al.*,[43] US	THA	Conventional cement	-
		Antibiotic-impregnated bone cement	Yes, more effective and less costly
Graves, *et al.*,[44] UK§	THA	No systemic antibiotics, plain cement and conventional ventilation	
		Systemic antibiotics, antibiotic-impregnated cement and conventional ventilation	Yes, more effective and less costly
Merollini, *et al.*,[45] Australia	THA	No antibiotic prophylaxis OR antibiotic prophylaxis OR antibiotic prophylaxis and laminar airflow	
		Antibiotic prophylaxis and antibiotic-impregnated cement	Yes, more effective and less costly
**Postoperative**			
Fusco & Turchetti,[46] Italy	TKA	20 face-to-face rehabilitation sessions	-
		10 face-to-face rehabilitation sessions plus 10 telesessions	Yes, same effectiveness but less costly
Kauppila, *et al.*,[47] Finland	TKA	Conventional orthopaedic care	-
		Multidisciplinary biopsychosocial outpatient rehabilitation programme	No
Bolz, *et al.*,[48] Australia††	THA	2-yearly routine follow-up OR follow-up at 3 months and 1 or 2 years	
		No follow-up	Yes

For peer review only

Whole recovery pathway

Two studies considered multiple components of the enhanced recovery pathway. The first study was an economic analysis of a randomized trial with 56 THA and 31 TKA participants in Denmark.[37] The pathway used in the treatment arm ("accelerated care") had no difference from the control group ("conventional rehabilitation") in terms of intraoperative management, analgesia, nausea control or bowel regulation. Differences in the treatment protocols between the two arms involved patient education, nutrition, admission times, staffing, and mobilization (described in Supplementary Table A9). The accelerated care pathway was the dominant strategy both overall and in the subgroup of THA patients (i.e. less costly and more effective than the control group). For TKA patients, the authors found the accelerated care pathway to be cost-saving but less effective compared to the control group although not statistically significant. There was a cost saving of 618 075 Danish krone per QALY lost with accelerated care compared to conventional rehabilitation, which made it cost-effective (threshold of 160 000 krone per QALY in Denmark).

The second study was an economic evaluation of a before and after trial with 98 THA and 62 TKA participants in The Netherlands.[36] The intervention ('Joint Recovery Programme') consisted of a 20-minute pre-assessment screening 6 weeks before the operation for physical assessment and analysis of the home situation to aid discharge planning, patient education sessions one to two weeks before surgery; group rehabilitation sessions, and supervision by physical therapists and nurses (Supplementary Table A9). Patients in the 'usual care' group underwent conventional physiotherapy for one hour daily, did not receive pre-assessment screening or information sessions, and discharge arrangements were addressed during admission to hospital. The joint recovery programme intervention was dominant for both hip and knee replacement, resulting in a cost saving of 1261 United States dollars (USD) per patient for THA and 3336 USD per patient for TKA, with no statistically significant difference in QALYs. The probability that the joint recovery programme was the most cost-effective option was above 80% for THA and TKA for willingness to pay thresholds up to 45 000 USD.

For peer review only - http://bmjopen.bmj.com/site/about/guidelines.xhtml

Preoperative components

McLawhorn and colleagues[38] used a Markov model to assess the cost-effectiveness of bariatric surgery two years before TKA for morbidly obese (body mass index ≥35 kg/m^2) patients who were candidates for both operations due to end-stage knee osteoarthritis and failed non-operative weight-loss interventions. The strategy including bariatric surgery was cost-effective at the stated willingness to pay threshold of 100 000 USD per QALY in 98.8% of probabilistic simulations.

Courville and colleagues[39] compared three strategies of screening for and treating *Staphylococcus aureus* colonization to prevent deep surgical site infections for TKA and THA. They found that decolonization of all preoperative patients with mupirocin, without testing for *S. aureus*, was the dominant strategy (less costly and more effective) when compared to treating only patients testing positive for *S. aureus*, or no screening or decolonization for *S. aureus*.

Intraoperative components

We found two studies that investigated strategies to reduce allogenic blood transfusions, either by collecting autologous blood prior to surgery,[41] or by aseptic collection of wound drainage.[40] The former was found to be cost-effective at 2750 USD per QALY gained, whereas the latter was not, costing 5.7 million USD per QALY gained. However, in the latter study, the only benefit of avoiding allogenic transfusion considered was a reduced risk of blood-borne virus infection.[40] In contrast, Sonnenberg's model for THA[41] found a minimal effect from the risk of blood-borne virus infection in either costs or outcomes: 99.6% of the increase in QALYs was due to a reduced risk of bacterial infection. When the risk of bacterial infection was removed from this model, the ICER increased to 2.5 million USD per QALY gained.

Marques and colleagues conducted economic evaluations[42] alongside two randomized controlled trials of adding local wound infiltration with bupivacaine to usual anaesthetic care for THA and TKA.[51] The infiltration of local anaesthetic was found to be dominant compared to standard anaesthesia in both THA and TKA patients.

Three studies used Markov models to investigate measures to reduce surgical site infection

For peer review only - http://bmjopen.bmj.com/site/about/guidelines.xhtml

in the United States,[43] United Kingdom,[44] and Australia.[45] The dominant strategy in all three included use of antibiotic-impregnated cement. The two studies that looked at other factors[44 45] each found use of prophylactic systemic antibiotics to be dominant over non-use, and use of conventional ventilation in operating theatres to be dominant over laminar airflow ventilation. Graves and colleagues[44] considered use of body exhaust suits, and found them to be dominated (more costly and less effective) by strategies that did not include use of these suits.

Postoperative components

Fusco and Turchetti[46] used a Markov model to evaluate a strategy of ten face-to-face rehabilitation sessions followed by ten telerehabilitation sessions after TKA, compared to twenty face-to-face sessions. They found the strategy including telerehabilitation to be cost saving, and improved range of movement (knee flexion). However, they found no utility data for patients following a telerehabilitation programme so for their base case assumed it to be non-inferior to face-to-face rehabilitation. In a sensitivity analysis, if telerehabilitation conferred an improvement in quality of life of at least 2.5%, the strategy's probability of being cost-effective was 1; if it led to a reduction in quality of life of at least 2.5%, the probability of being cost-effective was 0 (each at a willingness to pay threshold of 30 000 euros per QALY).

Kauppila and colleagues[47] performed an economic evaluation of a ten-day outpatient rehabilitation course between two and four months after TKA that included clinical assessments, physical activity, sessions with a psychologist, and lectures from an orthopaedic surgeon, nutritionist and social worker.[52] They found that patients who completed this course had higher costs and slightly worse quality of life outcomes over the 1 year of follow-up (though the difference was non-significant at the 5% level) compared to those receiving conventional orthopaedic care.

Bolz and colleagues[48] compared three follow-up strategies: two-yearly routine follow-up; follow-up twice (at three months, and between one and two years after surgery); or no follow-up. The model assumed no revisions would be delayed in either strategy that included follow-up, and the outcomes for these two strategies were identical in each analysis. The no follow-up strategy was

For peer review only - http://bmjopen.bmj.com/site/about/guidelines.xhtml

dominant for any assumed rate of delayed revision between 1% and 50%.

Effects of standardizing currencies and price years

Most studies showed one strategy to be dominant over the others[36 37 39 42-48] and were therefore not affected by changes in currency or price year. When converted into 2016 US PPP, willingness to pay thresholds ranged between 22 112 (Denmark) and 100 000 dollars (US; Supplementary Table A10). Autologous blood transfusion[41] and bariatric surgery[38] would be cost-effective across all countries identified in the review. The capture and replacement of red cells[40] cost 7.8 million US PPP dollars per QALY gained and would not be cost-effective by any study's thresholds. Accelerated care pathway in TKA patients in Denmark[37] would not be cost-effective in a US setting using a willingness to pay of 100 000 dollars per QALY.

DISCUSSION

Summary of evidence

Our objective was to assess the cost-effectiveness evidence of enhanced recovery following THA or TKA through a systematic collection of published cost–utility data. Previous systematic reviews considered effectiveness[15] and patient satisfaction,[56] but we believe this is the first systematic review assessing the cost-effectiveness of enhanced recovery for THA or TKA patients. We identified two cost–utility analyses of an entire pathway. This is consistent with reviews of cost-effectiveness of enhanced recovery programmes for other surgical sites,[57-59] which have found few studies reported the effect on quality of life and none presented cost-effectiveness results using QALYs. Both studies that considered cost-effectiveness of an entire recovery pathway were trials in both TKA and THA patients.[36 37] The enhanced recovery pathway was found to be associated with reduced costs for all patients and the incremental cost-effectiveness estimate favoured the enhanced recovery protocol, with a high probability of being the most cost-effective option.

For peer review only - http://bmjopen.bmj.com/site/about/guidelines.xhtml

We identified 11 studies presenting cost–utility data for components of an enhanced recovery pathway. However, these cover only a few of the potential enhanced recovery pathway components and the available evidence was mostly based on single studies. These studies covered a variety of interventions, across different healthcare systems, and used different cost perspectives. Three studies investigated overlapping strategies for reducing surgical infections supporting the use of prophylactic systemic antibiotics, antibiotic-impregnated cement, and conventional ventilation.[43-45] No other interventions were examined by more than one study. Scope to combine or generalize results is therefore limited.

The studies identified in this review were generally of good quality according to the CHEC list[29] with a short time horizon identified as a key limitation in six studies. When the models were assessed against the ISPOR questionnaire,[30] there were concerns about the lack of model validation work, potentially questioning the reliability of nine of the thirteen studies identified. The trials were generally of good quality. One trial was a non-randomized before and after trial,[36] and participants were not blinded to the intervention for postoperative interventions in any of the studies. However, we concluded that the overall risk of bias among the studies appeared low.

Limitations of this review

We may also have missed relevant evidence by limiting our search to reports published in the English language and excluding studies that did not report QALYs. However, we feel this was justified as cost–utility analyses enable comparison between different interventions and are the preferred intelligence for healthcare allocation decisions.[17 18] Of the studies excluded due to not reporting QALYs, one investigated a complete enhanced recovery pathway.[60] This was a retrospective review of TKA patients in Turkey who followed either a rapid-recovery protocol or a standard care protocol. The rapid-recovery protocol was found to be cost-saving and associated with statistically significant differences in knee flexion and extension at six months, although the clinical significance of these differences was not discussed and no health-related quality of life data

For peer review only - http://bmjopen.bmj.com/site/about/guidelines.xhtml

were collected.[60]

A second limitation is that standard practices have changed during the period covered by the included studies. For example, many enhanced recovery techniques are now standard practice,[12] and in more recent studies may be included as part of the 'usual practice' control arm.[8-10 14 51] Also, practices such as blood transfusion rates have changed greatly: the two studies included in this review assumed transfusion rates of 89%[41] to 100%,[40] whereas current rates are 22% for THA and 18% for TKA patients,[61] and current rates of infection from blood transfusion are lower than those used in the model.[62]

CONCLUSIONS

There is limited cost–utility evidence, either for an entire enhanced recovery pathway or for individual components of a pathway, for patients having THA or TKA. There are also concerns regarding the ability of short time horizons in trials in this area to capture relevant outcomes, and regarding a general lack of reporting of model validation. Our findings support the use of enhanced recovery pathways as a whole, prophylactic systemic antibiotics, antibiotic-impregnated cement and conventional ventilation. However, we were unable to conclude how transferable these findings would be into other healthcare systems. There is ample scope for future cost-effectiveness studies into enhanced recovery for THA and TKA patients. In particular, we recommend analysis of entire enhanced recovery pathways and comparison of incremental changes of components within pathways rather than considering aspects of care in isolation.

For peer review only - http://bmjopen.bmj.com/site/about/guidelines.xhtml

ACKNOWLEDGEMENTS

We are grateful to Nia Roberts, information specialist, and Rafael Pinedo-Villanueva, senior research associate in health economics, both at the University of Oxford, for their assistance with the search strategy.

AUTHOR STATEMENT:

JM, AJ and JL designed the study and all authors defined the inclusion criteria. JM, LYC and JL developed the search strategies. JM and LYC piloted the search strategy and inclusion criteria. JM and JL developed the data extraction pro forma. JM, LYC and JL screened the studies. JM, MGP and RJ extracted the data. MGP wrote the draft of the manuscript. JM and JL provided critical revisions to the article and all authors approved the final version of the article to be published. JL is the guarantor of the review.

DATA STATEMENT:

No data sharing available.

PATIENT CONSENT:

Not required.

PROVENANCE AND PEER REVIEW

Not commissioned; externally peer reviewed.

For peer review only - http://bmjopen.bmj.com/site/about/guidelines.xhtml

For peer review only

FIGURE LEGENDS:

Figure 1. Flow diagram of studies included in this review and reasons for exclusion, modified from Moher, *et al.*[27] (NHS EED, National Health Service Economic Evaluations Database; QALY, quality-adjusted life year)

Figure 2. Assessments of study quality based on tools from (*a*) Consensus on Health Economic Criteria,[29] (*b*) International Society for Pharmacoeconomics and Outcomes Research,[30] (*c*) Assessment of the Validation Status of Health-Economic decision models tool, (*d*) Cochrane Collaboration.[32] (Data from included studies[36-48] and reports of included trials.[49 51 52] Note, the study by Brunenberg and colleagues[36] was a non-randomised before and after trial: we have included it in this figure for completeness.)

For peer review only - http://bmjopen.bmj.com/site/about/guidelines.xhtml

REFERENCES

1. Pabinger C, Geissler A. Utilization rates of hip arthroplasty in OECD countries. *Osteoarthritis Cartilage* 2014;22(6):734-41. doi: 10.1016/j.joca.2014.04.009
2. Pabinger C, Lothaller H, Geissler A. Utilization rates of knee-arthroplasty in OECD countries. *Osteoarthritis Cartilage* 2015;23(10):1664-73. doi: 10.1016/j.joca.2015.05.008
3. Ackerman IN, Bohensky MA, de Steiger R, et al. Lifetime risk of primary total hip replacement surgery for osteoarthritis from 2003-2013: A multi-national analysis using national registry data. *Arthritis Care Res* 2017;Accepted manuscript online doi: 10.1002/acr.23197
4. Ackerman IN, Bohensky MA, de Steiger R, et al. Substantial rise in the lifetime risk of primary total knee replacement surgery for osteoarthritis from 2003 to 2013: an international, population-level analysis. *Osteoarthritis Cartilage* 2017;25(4):455-61. doi: 10.1016/j.joca.2016.11.005 [published Online First: 2016/11/20]
5. Culliford DJ, Maskell J, Kiran A, et al. The lifetime risk of total hip and knee arthroplasty: results from the UK general practice research database. *Osteoarthritis Cartilage* 2012;20(6):519-24. doi: 10.1016/j.joca.2012.02.636 [published Online First: 2012/03/08]
6. Culliford D, Maskell J, Judge A, et al. Future projections of total hip and knee arthroplasty in the UK: results from the UK Clinical Practice Research Datalink. *Osteoarthritis Cartilage* 2015;23(4):594-600. doi: 10.1016/j.joca.2014.12.022 [published Online First: 2015/01/13]
7. Kehlet H. Multimodal approach to control postoperative pathophysiology and rehabilitation. *Br J Anaesth* 1997;78(5):606-17. doi: 10.1093/bja/78.5.606
8. McDonald D, Siegmeth R, Deakin A, et al. An enhanced recovery programme for primary total knee arthroplasty in the United Kingdom—follow up at one year. *Knee* 2012;19(5):525-29. doi: 10.1016/j.knee.2011.07.012
9. Auyong DB, Allen CJ, Pahang JA, et al. Reduced length of hospitalization in primary total knee arthroplasty patients using an updated enhanced recovery after orthopedic surgery (ERAS) pathway. *J Arthroplast* 2015;30(10):1705-09. doi: 10.1016/j.arth.2015.05.007
10. Malviya A, Martin K, Harper I, et al. Enhanced recovery program for hip and knee replacement reduces death rate: A study of 4,500 consecutive primary hip and knee replacements. *Acta Orthop* 2011;82(5):577-81. doi: 10.3109/17453674.2011.618911
11. Dwyer AJ, Thomas W, Humphry S, et al. Enhanced recovery programme for total knee replacement to reduce the length of hospital stay. *J Orthop Surg* 2014;22(2):150-54. doi: 10.1177/230949901402200206
12. Nagra NS, Hamilton TW, Strickland L, et al. Enhanced recovery programmes for lower limb arthroplasty in the UK. *Ann R Coll Surg Engl* 2017:1-6. doi: 10.1308/rcsann.2017.0124 [published Online First: 2017/08/05]
13. Stowers MD, Lemanu DP, Coleman B, et al. Review article: Perioperative care in enhanced recovery for total hip and knee arthroplasty. *J Orthop Surg* 2014;22(3):383-92. doi: 10.1177/230949901402200324
14. Maempel J, Walmsley P. Enhanced recovery programmes can reduce length of stay after total knee replacement without sacrificing functional outcome at one year. *Ann R Coll Surg Engl* 2015;97(8):563-67. doi: 10.1308/rcsann.2015.0016
15. Zhu S, Qian W, Jiang C, et al. Enhanced recovery after surgery for hip and knee arthroplasty: a systematic review and meta-analysis. *Postgrad Med J* 2017:Epub ahead of print. doi: 10.1136/postgradmedj-2017-134991
16. Stowers MDJ, Manuopangai L, Hill AG, et al. Enhanced Recovery After Surgery in elective hip and knee arthroplasty reduces length of hospital stay. *ANZ J Surg* 2016;86(6):475-79. doi: 10.1111/ans.13538
17. National Institute for Health and Care Excellence. Guide to the methods of technology appraisal 2013 2013 [Available from: https://www.nice.org.uk/process/pmg9/chapter/foreword accessed 6 September 2017.

For peer review only - http://bmjopen.bmj.com/site/about/guidelines.xhtml

18. Weinstein MC, Siegel JE, Gold MR, et al. Recommendations of the panel on cost-effectiveness in health and medicine. *JAMA* 1996;276(15):1253-58. doi: 10.1001/jama.1996.03540150055031
19. Schwarzer R, Rochau U, Saverno K, et al. Systematic overview of cost-effectiveness thresholds in ten countries across four continents. *J Comp Eff Res* 2015;4(5):485-504. doi: 10.2217/cer.15.38 [published Online First: 2015/10/23]
20. Nwachukwu BU, Bozic KJ, Schairer WW, et al. Current status of cost utility analyses in total joint arthroplasty: a systematic review. *Clin Orthop Relat Res* 2015;473(5):1815-27. doi: 10.1007/s11999-014-3964-4 [published Online First: 2014/10/01]
21. Daigle ME, Weinstein AM, Katz JN, et al. The cost-effectiveness of total joint arthroplasty: a systematic review of published literature. *Best Pract Res Clin Rheumatol* 2012;26(5):649-58. doi: 10.1016/j.berh.2012.07.013 [published Online First: 2012/12/12]
22. Brockbank J, Wolowacz S. Economic evaluations of new oral anticoagulants for the prevention of venous thromboembolism after total hip or knee replacement: A systematic review. *Pharmacoeconomics* 2017;35:517-35. doi: 10.1007/s40273-017-0486-4
23. Kapoor A, Chuang W, Radhakrishnan N, et al. Cost effectiveness of venous thromboembolism pharmacological prophylaxis in total hip and knee replacement: A systematic review. *Pharmacoeconomics* 2010;28(7):521-38. doi: 10.2165/11535210-000000000-00000
24. Wolowacz S, Hess N, Brennan V, et al. Cost-effectiveness of venous thromboembolism prophylaxis in total hip and knee replacement surgery: the evolving application of health economic modelling over 20 years. *Curr Med Res Opin* 2008;24(10):2993-3006. doi: 10.1185/03007990802443255
25. Murphy J, Pritchard MG, Cheng LY, et al. Cost-effectiveness of enhanced recovery in hip and knee replacement: a systematic review protocol. *BMJ Open* 2018;8(3):e019740. doi: 10.1136/bmjopen-2017-019740 [published Online First: 2018/03/16]
26. Murphy J, Cheng, LY., Leal, J. Cost-effectiveness and quality of life of enhanced recovery in hip and knee replacement: a systematic review protocol. PROSPERO 2017:CRD42017059473 Available from http://www.crd.york.ac.uk/PROSPERO/display_record.asp?ID=CRD42017059473. PROSPERO International prospective register of systematic reviews, 2017.
27. Moher D, Liberati A, Tetzlaff J, et al. Preferred reporting items for systematic reviews and meta-analyses: the PRISMA statement. *PLOS Med* 2009;6(7):e1000097. doi: 10.1371/journal.pmed.1000097
28. National Joint Registry. 13th annual report 2016: National Joint Registry for England, Wales, Northern Ireland and the Isle of Man surgical data to 31 December 2015. Hemel Hempstead, UK: National Joint Registry, 2016.
29. Evers S, Goossens M, de Vet H, et al. Criteria list for assessment of methodological quality of economic evaluations: Consensus on Health Economic Criteria. *Int J Technol Assess Health Care* 2005;21(2):240-5. [published Online First: 2005/06/01]
30. Caro JJ, Eddy DM, Kan H, et al. Questionnaire to assess relevance and credibility of modeling studies for informing health care decision making: an ISPOR-AMCP-NPC Good Practice Task Force report. *Value Health* 2014;17(2):174-82. doi: 10.1016/j.jval.2014.01.003.
31. Vemer P, Corro Ramos I, van Voorn GA, et al. AdViSHE: A validation-assessment tool of health-economic models for decision makers and model users. *Pharmacoeconomics* 2016;34(4):349-61. doi: 10.1007/s40273-015-0327-2 [published Online First: 2015/12/15]
32. Higgins JP, Altman DG, Gøtzsche PC, et al. The Cochrane Collaboration's tool for assessing risk of bias in randomised trials. *BMJ* 2011;343:d5928. doi: 10.1136/bmj.d5928 [published Online First: 2011/10/20]
33. Cooper N, Coyle D, Abrams K, et al. Use of evidence in decision models: an appraisal of health technology assessments in the UK since 1997. *J Health Serv Res Policy* 2005;10(4):245-50. doi: 10.1258/135581905774414187

For peer review only - http://bmjopen.bmj.com/site/about/guidelines.xhtml

34. Coyle D, Lee KM. Evidence-based economic evaluation: how the use of different data sources can impact results. In: Donaldson C, Mugford M, Vale L, eds. Evidence-based health economics: from effectiveness to efficiency in systematic review. London, UK: BMJ Publishing Group 2002:55-66.
35. Organisation for Economic Co-operation and Development. Prices and purchasing power parities 2017 [Available from: http://stats.oecd.org/Index.aspx?DataSetCode=CPL accessed 19 September 2017.
36. Brunenberg DE, van Steyn MJ, Sluimer JC, et al. Joint recovery programme versus usual care: an economic evaluation of a clinical pathway for joint replacement surgery. *Medical care* 2005;43(10):1018-26. [published Online First: 2005/09/17]
37. Larsen K, Hansen TB, Thomsen PB, et al. Cost-effectiveness of accelerated perioperative care and rehabilitation after total hip and knee arthroplasty. *J Bone Joint Surg Am* 2009;91(4):761-72. doi: 10.2106/JBJS.G.01472
38. McLawhorn AS, Southren D, Wang YC, et al. Cost-effectiveness of bariatric surgery prior to total knee arthroplasty in the morbidly obese: A computer model-based evaluation. *J Bone Joint Surg Am* 2016;98(2):e6. doi: 10.2106/JBJS.N.00416
39. Courville XF, Tomek IM, Kirkland KB, et al. Cost-effectiveness of preoperative nasal mupirocin treatment in preventing surgical site infection in patients undergoing total hip and knee arthroplasty: a cost-effectiveness analysis. *Infect Control Hosp Epidemiol* 2012;33(2):152-59. doi: 10.1086/663704
40. Jackson BR, Umlas J, AuBuchon JP. The cost-effectiveness of postoperative recovery of RBCs in preventing transfusion-associated virus transmission after joint arthroplasty. *Transfusion* 2000;40(9):1063-6.
41. Sonnenberg FA. A health economic analysis of autologous transfusion. *Infus Ther Transfus Med* 2002;29(3):175-83.
42. Marques EMR, Blom AW, Lenguerrand E, et al. Local anaesthetic wound infiltration in addition to standard anaesthetic regimen in total hip and knee replacement: Long-term cost-effectiveness analyses alongside the APEX randomised controlled trials. *BMC Med* 2015;13:151. doi: 10.1186/s12916-015-0389-1
43. Cummins JS, Tomek IM, Kantor SR, et al. Cost-effectiveness of antibiotic-impregnated bone cement used in primary total hip arthroplasty. *J Bone Joint Surg Am* 2009;91(3):634-41. doi: 10.2106/JBJS.G.01029
44. Graves N, Wloch C, Wilson J, et al. A cost-effectiveness modelling study of strategies to reduce risk of infection following primary hip replacement based on a systematic review. *Health Technol Assess* 2016;20(54):1-144. doi: 10.3310/hta20540
45. Merollini KM, Crawford RW, Whitehouse SL, et al. Surgical site infection prevention following total hip arthroplasty in Australia: a cost-effectiveness analysis. *Am J Infect Control* 2013;41(9):803-9. doi: 10.1016/j.ajic.2012.11.015
46. Fusco F, Turchetti G. Telerehabilitation after total knee replacement in Italy: cost-effectiveness and cost-utility analysis of a mixed telerehabilitation-standard rehabilitation programme compared with usual care. *BMJ Open* 2016;6(5):e009964. doi: 10.1136/bmjopen-2015-009964
47. Kauppila AM, Sintonen H, Aronen P, et al. Economic evaluation of multidisciplinary rehabilitation after primary total knee arthroplasty based on a randomized controlled trial. *Arthritis Care Res* 2011;63(3):335-41. doi: 10.1002/acr.20398
48. Bolz KMD, Crawford RW, Donnelly B, et al. The cost-effectiveness of routine follow-up after primary total hip arthroplasty. *J Arthroplast* 2010;25(2):191-96. doi: 10.1016/j.arth.2008.12.009
49. Larsen K, Sørensen OG, Hansen TB, et al. Accelerated perioperative care and rehabilitation intervention for hip and knee replacement is effective: a randomized clinical trial involving 87 patients with 3 months of follow-up. *Acta Orthop* 2008;79(2):149-59. doi:

For peer review only - http://bmjopen.bmj.com/site/about/guidelines.xhtml

10.1080/17453670710014923 [published Online First: 2008/05/15]
50. Sintonen H. The 15D instrument of health-related quality of life: properties and applications. *Annals of medicine* 2001;33(5):328-36. [published Online First: 2001/08/09]
51. Wylde V, Lenguerrand E, Gooberman-Hill R, et al. Effect of local anaesthetic infiltration on chronic postsurgical pain after total hip and knee replacement: the APEX randomised controlled trials. *Pain* 2015;156(6):1161-70. doi: 10.1097/j.pain.0000000000000114
52. Kauppila A-M, Kyllönen E, Ohtonen P, et al. Multidisciplinary rehabilitation after primary total knee arthroplasty: a randomized controlled study of its effects on functional capacity and quality of life. *Clin Rehabil* 2010;24(5):398-411. doi: 10.1177/0269215509346089
53. Briggs A, Sculpher M, Britton A, et al. The costs and benefits of primary total hip replacement. How likely are new prostheses to be cost-effective? *Int J Technol Assess Health Care* 1998;14(4):743-61. [published Online First: 1999/01/14]
54. Birkmeyer JD, Goodnough LT, AuBuchon JP, et al. The cost-effectiveness of preoperative autologous blood donation for total hip and knee replacement. *Transfusion* 1993;33(7):544-51.
55. Sonnenberg FA, Gregory P, Yomtovian R, et al. The cost-effectiveness of autologous transfusion revisited: implications of an increased risk of bacterial infection with allogeneic transfusion. *Transfusion* 1999;39(8):808-17.
56. Jones E, Wainwright T, Foster J, et al. A systematic review of patient reported outcomes and patient experience in enhanced recovery after orthopaedic surgery. *Ann R Coll Surg Engl* 2014;96(2):89-94. doi: 10.1308/003588414x13824511649571
57. Stowers MD, Lemanu DP, Hill AG. Health economics in Enhanced Recovery After Surgery programs. *Can J Anaesth* 2015;62(2):219-30. doi: 10.1007/s12630-014-0272-0 [published Online First: 2014/11/14]
58. Lemanu DP, Singh PP, Stowers MD, et al. A systematic review to assess cost effectiveness of enhanced recovery after surgery programmes in colorectal surgery. *Colorectal Dis* 2014;16(5):338-46. doi: 10.1111/codi.12505 [published Online First: 2013/11/29]
59. Lee L, Li C, Landry T, et al. A systematic review of economic evaluations of enhanced recovery pathways for colorectal surgery. *Ann Surg* 2014;259(4):670-6. doi: 10.1097/SLA.0b013e318295fef8 [published Online First: 2013/05/16]
60. Köksal I, Tahta M, Şimşek ME, et al. Efficacy of rapid recovery protocol for total knee arthroplasty: a retrospective study. *Acta Orthop Traumatol Turc* 2015;49(4):382-6. doi: 10.3944/AOTT.2015.14.0353
61. Hart A, Khalil JA, Carli A, et al. Blood transfusion in primary total hip and knee arthroplasty. Incidence, risk factors, and thirty-day complication rates. *J Bone Joint Surg Am* 2014;96(23):1945-51. doi: 10.2106/JBJS.N.00077
62. Norfolk D, editor. *Handbook of transfusion medicine*. Norwich, UK: TSO, 2013.

For peer review only

For peer review only - http://bmjopen.bmj.com/site/about/guidelines.xhtml

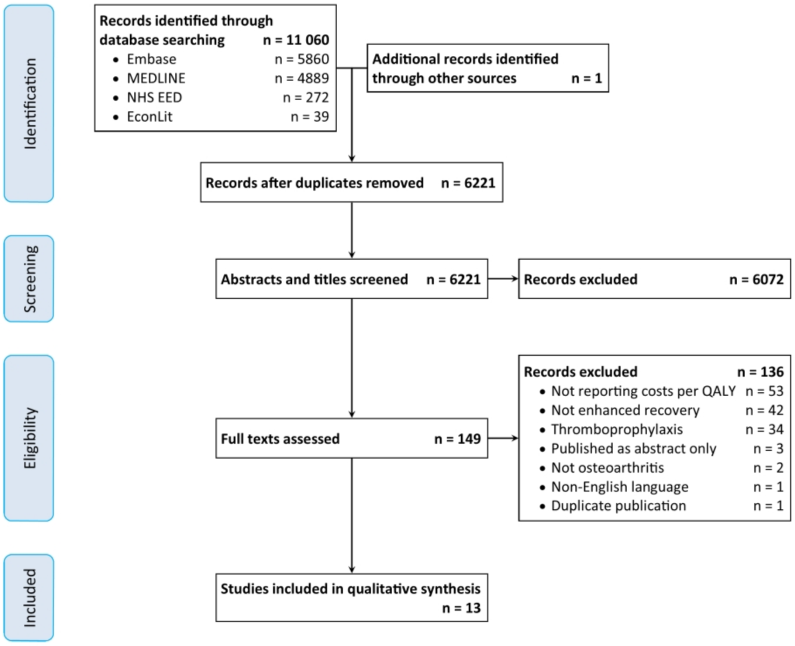

Figure 1. Flow diagram of studies included in this review and reasons for exclusion, modified from Moher, et al.27 (NHS EED, National Health Service Economic Evaluations Database; QALY, quality-adjusted life year)

209x297mm (300 x 300 DPI)

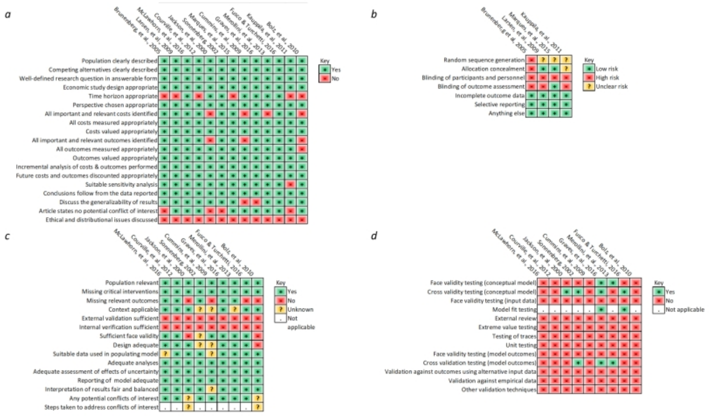

Figure 2. Assessments of study quality based on tools from (a) Consensus on Health Economic Criteria,29 (b) International Society for Pharmacoeconomics and Outcomes Research,30 (c) Assessment of the Validation Status of Health-Economic decision models tool, (d) Cochrane Collaboration.32 (Data from included studies36-48 and reports of included trials.49 51 52 Note, the study by Brunenberg and colleagues36 was a non-randomised before and after trial: we have included it in this figure for completeness.)

297x209mm (300 x 300 DPI)

Supplemental Material: Enhanced recovery following hip and knee arthroplasty

Table A1. Full search terms for databases included in this review

Query	Search term
Ovid MEDLINE	
1	arthroplasty, replacement, hip/ or arthroplasty, replacement, knee/
2	((knee? or hip) adj (replace$ or arthroplast*)).ti,ab.
3	1 or 2
4	simulation model$.ti,ab.
5	markov.ti,ab.
6	monte carlo.ti,ab.
7	decision tree$.ti,ab.
8	decision analy$.ti,ab.
9	qaly$.ti,ab.
10	(valu$ adj2 quality).ti,ab.
11	utility value$.ti,ab.
12	((disability or quality) adj adjusted).ti,ab.
13	((life adj2 year$) or health year equivalent$).ti,ab.
14	(health adj utilit$).ti,ab.
15	hui$1.ti,ab.
16	(quality adj3 well$).ti,ab.
17	qwb.ti,ab.
18	(qald$ or qale$ or qtime$).ti,ab.
19	(well being or wellbeing).tw.
20	(health adj2 stat$).tw.
21	((adjusted adj2 life) or qaly$).ti,ab.
22	(daly or qol or hql or hqol or hrqol or hr ql or hrql).tw.
23	cost-utility.ti,ab.
24	cost-effectiveness.ti,ab.
25	cost-benefit.ti,ab.
26	cost-minimisation.ti,ab.
27	cost-minimization.ti,ab.
28	modelling.ti,ab.
29	modeling.ti,ab.
30	decision model.ti,ab.
31	QALY.ti,ab.
32	quality adjusted life year$.ti,ab.
33	cost.ti,ab.
34	life year$.ti,ab.
35	incremental cost-effectiveness ratio.ti,ab.
36	(quality adj2 life).ti,ab.
37	Technology Assessment, Biomedical/
38	"Costs and Cost Analysis"/
39	technology assessment$.ti,ab.
40	economic evaluation$.ti,ab.
41	economic model$.ti,ab.
42	discrete event simulat$.ti,ab.
43	cost utility.ti,ab.
44	cost effectiv$.ti,ab.
45	cost benefit.ti,ab.
46	cost minimisation.ti,ab.
47	cost minimization.ti,ab.
48	ICER$.ti,ab.
49	EQ-5D$.ti,ab.
50	(SF-12 or SF12 or Short Form 12).ti,ab.
51	(SF-36 or SF36 or Short Form 36).ti,ab.
52	(SF-6D or SF6D or Short Form 6D).ti,ab.

For peer review only

For peer review only - http://bmjopen.bmj.com/site/about/guidelines.xhtml

Supplemental Material: Enhanced recovery following hip and knee arthroplasty

53	rosser index.ti,ab.
54	person trade off.ti,ab.
55	standard gamble.ti,ab,kw.
56	time trade off.ti,ab,kw.
57	Hye.ti,ab,kw.
58	Hyes.ti,ab,kw.
59	Euroquol.ti,ab,kw.
60	4 or 5 or 6 or 7 or 8 or 9 or 10 or 11 or 12 or 13 or 14 or 15 or 16 or 17 or 18 or 19 or 20 or 21 or 22 or 23 or 24 or 25 or 26 or 27 or 28 or 29 or 30 or 31 or 32 or 33 or 34 or 35 or 36 or 37 or 38 or 39 or 40 or 41 or 42 or 43 or 44 or 45 or 46 or 47 or 48 or 49 or 50 or 51 or 52 or 53 or 54 or 55 or 56 or 57 or 58 or 59
61	3 and 60
EMBASE	
1	hip replacement/ or hip arthroplasty/
2	total knee replacement/ or knee replacement/ or knee arthroplasty/
3	((knee? or hip) adj (replace$ or arthroplast$)).ti,ab.
4	1 or 2 or 3
5	simulation model$.ti,ab.
6	markov.ti,ab.
7	monte carlo.ti,ab.
8	decision tree$.ti,ab.
9	decision analy$.ti,ab.
10	qaly$.ti,ab.
11	(valu$ adj2 quality).ti,ab.
12	utility value$.ti,ab.
13	((disability or quality) adj adjusted).ti,ab.
14	((life adj2 year$) or health year equivalent$).ti,ab.
15	hui$1.ti,ab.
16	(quality adj3 well$).ti,ab.
17	qwb.ti,ab.
18	(qald$ or qale$ or qtime$).ti,ab.
19	(well being or wellbeing).tw.
20	(health adj2 stat$).tw.
21	((adjusted adj2 life) or qaly$).ti,ab.
22	(daly or qol or hql or hqol or hrqol or hr ql or hrql).tw.
23	cost-utility.ti,ab.
24	cost-benefit.ti,ab.
25	cost-minimisation.ti,ab.
26	cost-minimization.ti,ab.
27	modelling.ti,ab.
28	modeling.ti,ab.
29	QALY.ti,ab.
30	quality adjusted life year$.ti,ab.
31	cost.ti,ab.
32	life year$.ti,ab.
33	incremental cost-effectiveness ratio.ti,ab.
34	(quality adj2 life).ti,ab.
35	decision model$.ti,ab.
36	cost-effectiv$.ti,ab.
37	"cost benefit analysis"/
38	biomedical technology assessment/
39	technology assessment$.ti,ab.
40	economic evaluation$.ti,ab.
41	economic model$.ti,ab.
42	discrete event simulat$.ti,ab.
43	cost utility.ti,ab.
44	cost effectiv$.ti,ab.

For peer review only

For peer review only - http://bmjopen.bmj.com/site/about/guidelines.xhtml

Supplemental Material: Enhanced recovery following hip and knee arthroplasty

45	cost benefit.ti,ab.
46	cost minimisation.ti,ab.
47	cost minimization.ti,ab.
48	ICER$.ti,ab.
49	(health adj utilit$).ti,ab.
50	EQ-5D$.ti,ab.
51	(SF-12 or SF12 or Short Form 12).ti,ab.
52	(SF-36 or SF36 or Short Form 36).ti,ab.
53	(SF-6D or SF6D or Short Form 6D).ti,ab.
54	rosser index.ti,ab.
55	person trade off.ti,ab.
56	standard gamble.ti,ab,kw.
57	time trade off.ti,ab,kw.
58	Hye.ti,ab,kw.
59	Hyes.ti,ab,kw.
60	Euroquol.ti,ab,kw.
61	5 or 6 or 7 or 8 or 9 or 10 or 11 or 12 or 13 or 14 or 15 or 16 or 17 or 18 or 19 or 20 or 21 or 22 or 23 or 24 or 25 or 26 or 27 or 28 or 29 or 30 or 31 or 32 or 33 or 34 or 35 or 36 or 37 or 38 or 39 or 40 or 41 or 42 or 43 or 44 or 45 or 46 or 47 or 48 or 49 or 50 or 51 or 52 or 53 or 54 or 55 or 56 or 57 or 58 or 59 or 60
62	4 and 61
National Health Service Economic Evaluations Database, via the Cochrane Library	
Hip	Title, Abstract, Keywords: “Hip arthroplasty” OR
	Title, Abstract, Keywords: “Hip arthroplasties” OR
	Title, Abstract, Keywords: “Hip replacement”
Knee	Title, Abstract, Keywords: “Knee arthroplasty” OR
	Title, Abstract, Keywords: “Knee arthroplasties” OR
	Title, Abstract, Keywords: “Knee replacement”
EconLit	
	TI,AB(hip) OR TI,AB(knee) AND
	TI,AB(Replace*) OR TI,AB(arthroplasty*) OR TI,AB(Replacement) OR TI,AB(arthroplasties)

For peer review only

For peer review only - http://bmjopen.bmj.com/site/about/guidelines.xhtml

Supplemental Material: Enhanced recovery following hip and knee arthroplasty

Table A2. Hierarchy used to classify data sources used in studies, modified from Cooper and colleagues[1] and Coyle and colleagues[2]

*Source of data for clinical effect sizes, adverse events & complications**	
1	Meta-analysis of RCTs with direct comparison between comparator therapies, measuring final outcomes
2	Single RCT with direct comparison between comparator therapies, measuring final outcomes
3	Meta-analysis of RCTs with direct comparison between comparator therapies, measuring surrogate outcomes; *or* meta-analysis of placebo-controlled RCTs with similar trial populations, measuring final outcomes for each individual therapy
4	Single RCT with direct comparison between comparator therapies, measuring surrogate outcomes; *or* single placebo-controlled RCTs with similar trial populations, measuring final outcomes for each individual therapy
5	Meta-analysis of placebo-controlled RCTs with similar trial populations, measuring surrogate outcomes
6	Single placebo-controlled RCTs with similar trial populations, measuring surrogate outcomes for each individual therapy
7	Case–control or cohort studies
8	Non-analytic studies, for example, case reports, case series
9	Expert opinion
*Source of baseline clinical data**	
1	Case series or analysis of reliable administrative databases specifically conducted for the study covering patients solely from the jurisdiction of interest
2	Recent case series or analysis of reliable administrative databases covering patients solely from the jurisdiction of interest
3	Recent case series or analysis of reliable administrative databases covering patients solely from another jurisdiction
4	Old case series or analysis of reliable administrative databases; *or* estimates from RCTs
5	Estimates from previously published economic analyses: unsourced
6	Expert opinion
*Source of data for duration of primary effect**	
1	Analysis of reliable administrative databases specifically conducted for the study covering patients solely from the jurisdiction of interest
2	Recent analysis of reliable administrative databases covering patients solely from the jurisdiction of interest
3	Recent analysis of reliable administrative databases covering patients solely from another jurisdiction
4	Old analysis of reliable administrative databases
5	Estimates from previously published economic analyses: unsourced
6	Expert opinion
*Source of data for resource use**	
1	Prospective data collection; *or* analysis of reliable administrative data from same jurisdiction for specific study
2	Recently published results of prospective data collection from the same; *or* recent analysis of reliable administrative data from the same jurisdiction
3	Unsourced data from previous economic evaluations from the same jurisdiction
4	Recently published results of prospective data collection from a different jurisdiction; *or* recent analysis of reliable administrative data from a different jurisdiction
5	Unsourced data from previous economic evaluation from a different jurisdiction
6	Expert opinion
Source of data for costs	
1	Cost calculations based on reliable databases or data sources conducted for specific study—same jurisdiction
2	Recently published cost calculations based on reliable databases or data sources—same jurisdiction
3	Unsourced data from previous economic evaluation—same jurisdiction

For peer review only

For peer review only - http://bmjopen.bmj.com/site/about/guidelines.xhtml

Supplemental Material: Enhanced recovery following hip and knee arthroplasty

4	Recently published cost calculations based on reliable databases or data sources—different jurisdiction
5	Unsourced data from previous economic evaluation—different jurisdiction
6	Expert opinion
Source of data for utilities	
1	Direct utility assessment for the specific study from a sample either:<br>of the general population, or<br>with knowledge of the disease(s) of interest, or<br>of patients with the disease(s) of interest; *or*<br>indirect utility assessment for the specific study from patient sample with disease(s) of interest, using a tool validated for the patient population
2	Direct utility assessment from a previous study from a sample either:<br>of the general population, or<br>with knowledge of the disease(s) of interest, or<br>of patients with the disease(s) of interest; *or*<br>indirect utility assessment from a previous study from patient sample with disease(s) of interest, using a tool validated for the patient population
3	Indirect utility assessment from a patient sample with disease(s) of interest, using a tool not validated for the patient population; *or*<br>patient preference values obtained from a visual analogue scale
4	Delphi panels; *or*<br>expert opinion

* Hierarchy used for assessment of models only.
RCT, randomized controlled trial

For peer review only - http://bmjopen.bmj.com/site/about/guidelines.xhtml

Supplemental Material: Enhanced recovery following hip and knee arthroplasty

Table A3. Components of enhanced recovery pathway investigated by included studies and studies excluded due to not including costs per quality-adjusted life year

	Included studies	Excluded due to not including costs per QALY*			
		**Hip + knee**	**Hip**	**Knee**	**Total**
Entire pathway					
Entire pathway	2 [3,4]			1 [5]	1
Preoperative					
Optimization of comorbidities	1 [6]				0
Other preoperative interventions		1 [7]	1 [8]		2
Intraoperative					
Avoid unnecessary blood transfusion	2 [9,10]	3 [11-13]	1 [14]	4 [15-18]	8
Specified anaesthetic requirements		1 [19]	1 [20]	1 [21]	3
Local infiltration of analgesia	1 [22]				0
Tranexamic acid		3 [23-25]		1 [26]	4
Infection preventions	4 [27-30]	2 [31,32]	2 [33,34]		4
Postoperative					
Scheduled anti-emetic		1 [35]			1
Physical therapy	2 [36,37]	3 [38-40]	2 [41,42]	2 [43,44]	7
Aim for early discharge			1 [45]		1
Other postoperative interventions	1 [46]	1 [47]		2 [48,49]	3
**Totals**	**13**	**15**	**8**	**11**	**35**

* Studies of thromboprophylaxis have not been included on this table

QALY, quality-adjusted life year

For peer review only - http://bmjopen.bmj.com/site/about/guidelines.xhtml

Supplemental Material: Enhanced recovery following hip and knee arthroplasty

Table A4. Overview of studies included in the review.

Authors	Population	Perspective	Time horizon	Discounting	Currency, cost year
**Enhanced recovery pathway**					
Brunenberg, *et al.*[3]	Patients on the waiting list for either a THA (48 patients recruited to Joint Recovery Programme (JRP), 50 to usual care) or TKA (30 patients to JRP, 32 to usual care), mean age 64.4 years, one-third male	Societal	1 year	None	USD, 2002
Larsen, *et al.*[4]	Patients in trial[50] having THA, THA or UKA, mean age having accelerated intervention 64, 26 women, 30 men	Societal	1 year	None	DKK, 2006
**Preoperative:** *Assessment and optimization of comorbidities*					
McLawhorn, *et al.*[6]	50-year-old morbidly obese patient with end-stage unilateral knee osteoarthritis	Societal	40 years	3%	USD, 2012
**Preoperative:** Staphylococcus aureus *prophylaxis*					
Courville, *et al.*[27]	65-year-olds with end stage hip or knee osteoarthritis for whom medical management has failed and TJA recommended.	Societal	1 year	None	USD, 2005
**Intraoperative:** *Avoid unnecessary blood transfusion*					
Jackson, *et al.*[9]	Patients undergoing TJA, average age 65 years, range 20 to 80 years	Not stated	Lifetime	3% and 5%	USD, not reported
Sonnenberg[10]	65-year-old of composite sex and race undergoing THA	Not stated	Lifetime	3%	USD, 2000
**Intraoperative:** *Local infiltration of analgesia*					
Marques, *et al.*[22]	Patients in trial[51] having THA or TKA, mean ages 66–69.5 years, 52%–65% female	Health and social care payer	1 year	None	GBP, not reported
**Intraoperative:** *Infection prevention*					
Cummins, *et al.*[28]	68-year-olds of average health for their age undergoing THA due to degenerative arthritis	Hospital	Lifetime	3%	USD, 2002
Graves, *et al.*[29]	Simulated cohort of patients who had THA in 2012	UK NHS	Lifetime	3%	GBP, 2012
Merollini, *et al.*[30]	Not described	Healthcare system	30 years	3%	AUD, 2011
**Postoperative:** *Physical therapy*					
Fusco & Turchetti[36]	Average age of 60 years, 44% males, 19% experienced complications during or after surgery	Societal & healthcare service	Lifetime	3%	Euro, 2013
Kauppila, *et al.*[37]	Patients in trial aged 60–80 years having unilateral TKR for knee osteoarthritis	Healthcare system	1 year	None	Euro, 2006
**Postoperative:** *Scheduling of follow-up*					
Bolz, *et al.*[46]	THA patients, mean age 69.9 years	Health services	7 years	3%	AUD, not reported

NHS: National Health Service; THA: total hip arthroplasty; TJA: total joint arthroplasty; TKA: total knee arthroplasty; UKA: unicompartmental knee arthroplasty; AUD: Australian dollar; DKK: Danish krone; GBP: British pound; USD: US dollar

For peer review only - http://bmjopen.bmj.com/site/about/guidelines.xhtml
For peer review only

Supplemental Material: Enhanced recovery following hip and knee arthroplasty

Table A5. Costs included in each study

	Direct medical	Direct treatment	In-patient	Out-patient	Day care	Community healthcare	Medication	Side effect costs	Staff	Labs/diagnostic	Capital equipment	Social care	Travel costs	Productivity losses	Income forgone due to illness
Brunenberg, *et al.*[3]	+	+	+	+		+	+		+			+		+	+
Larsen, *et al.*[4]	+	+	+	+		+	+		+					+	+
McLawhorn, *et al.*[6]	+	+	+					+							
Courville, *et al.*[27]	+	+	+				+			+					
Jackson, *et al.*[9]	+	+	+	+			+		+	+					
Sonnenberg,[10]	+	+	+	+			+								
Marques, *et al.*[22]	+	+	+	+	+	+	+		+			+			
Cummins, *et al.*[28]	+	+	+												
Graves, *et al.*[29]	+	+	+				+				+				
Merollini, *et al.*[30]	+	+	+				+		+		+				
Fusco & Turchetti,[36]	+	+	+	+					+		+		+	+	+
Kauppila, *et al.*[37]	+	+	+	+	+	+						+			
Bolz, *et al.*[46]	+		+	+						+					

For peer review only

For peer review only - http://bmjopen.bmj.com/site/about/guidelines.xhtml

Supplemental Material: Enhanced recovery following hip and knee arthroplasty

Table A6. Populations and tools used for eliciting utilities.

Authors	EQ-5D	15 D	QWB	SG	VAS / rating scale	TTO	AQoL
Studies eliciting utilities							
Brunenberg, *et al.*[3]	Patients in trial (UK tariff)						
Larsen, *et al.*[4]	Patients in trial (Danish tariff)	—	—	—	—	—	—
Marques, *et al.*[22]	Patients in trial (UK tariff)	—	—	—	—	—	—
Kauppila, *et al.*[37]	—	Patients in trial (Finnish tariff)	—	—	—	—	—
Fusco & Turchetti[36]	TKR patients in trial (UK tariff)	—	—	—	—	—	—
Studies using published utilities[*]							
McLawhorn, *et al.*[6]	TKR patients in a trial; patients having bariatric surgery	—	—	—	Patients having bariatric surgery	—	—
Courville, *et al.*[27]	—	—	Population reporting arthritis	—	—	—	—
Jackson, *et al.*[9]	—	—	People with HIV infection	People with HIV infection	People with HIV infection	People with HIV infection	—
Sonnenberg[10]	—	—	People with HIV infection	—	—	—	—
Cummins, *et al.*[28]	—	—	—	—	—	THA patients	—
Graves, *et al.*[29]	THA patients	THA patients	—	—	—	—	THA patients with prosthesis infection
Merollini, *et al.*[30]	—	THA patients	—	—	—	—	THA patients with prosthesis infection
Bolz, *et al.*[46]	—	THA patients	—	—	—	—	—

* Details of utilities obtained from expert opinion are not included in this table

15-D, 15-dimension instrument; AQoL, Assessment of Quality of Life; EQ-5D, EuroQoL five-dimension instrument; HIV, human immunodeficiency virus; SG, standard gamble; THA, total hip arthroplasty; TKA, total knee arthroplasty; TTO, time trade-off; QWB, Quality of Well-being scale; VAS, visual analogue scale

For peer review only - http://bmjopen.bmj.com/site/about/guidelines.xhtml

For peer review only

Supplemental Material: Enhanced recovery following hip and knee arthroplasty

Table A7. Overview of quality and sources of data used by studies included in this analysis

	Sources of data											
	Clinical effect sizes		Baseline clinical data		Duration of primary effect		Resource use		Costs		Utilities	
Authors	Min	Max	Min	Max	Min	Max	Min	Max	Min	Max	Min	Max
Range of possible scores[*]	*1*	*9*	*1*	*6*	*1*	*6*	*1*	*6*	*1*	*6*	*1*	*4*
Brunenberg, *et al.*[3]	—[†]	—[†]	—[†]	—[†]	—[†]	—[†]	—[†]	—[†]	1	2	1	1
Larsen, *et al.*[4]	—[†]	—[†]	—[†]	—[†]	—[†]	—[†]	—[†]	—[†]	1	4	1	1
McLawhorn, *et al.*[6]	1	8	2	2	NR	NR	NR	NR	2	6	2	4
Courville, *et al.*[27]	1	1	1	1	NR	NR	2	2	2	2	2	2
Jackson, *et al.*[9]	8	8	2	2	NR	NR	NR	NR	4	4	2	2
Sonnenberg[10]	8	8	2	2	NR	NR	3	3	1	3	2	4
Marques, *et al.*[22]	—[†]	—[†]	—[†]	—[†]	—[†]	—[†]	—[†]	—[†]	1	2	1	1
Cummins, *et al.*[28]	8	8	3	3	NR	NR	2	2	2	4	2	2
Graves, *et al.*[29]	1	7	1	4	NR	NR	2	6	1	1	2	4
Merollini, *et al.*[30]	1	7	1	3	NR	NR	NR	NR	1	6	2	4
Fusco & Turchetti[36]	3	3	2	5	3	3	2	2	2	6	2	2
Kauppila, *et al.*[37]	—[†]	—[†]	—[†]	—[†]	—[†]	—[†]	—[†]	—[†]	1	2	1	1
Bolz, *et al.*[46]	NR	NR	2	2	NR	NR	NR	NR	2	2	2	2

Note: Where a study used several data sources within each category the results are reported as a range of the minimum to maximum data quality score within each category.
* Details of the full hierarchy are available in supplementary table A2 online.
† Not applicable as study is not model-based

CHEC, Consensus on Health Economic Criteria; ISPOR, International Society for Pharmacoeconomics and Outcomes Research; NR, not reported

For peer review only

For peer review only - http://bmjopen.bmj.com/site/about/guidelines.xhtml

Supplemental Material: Enhanced recovery following hip and knee arthroplasty

Table A8. Summary of findings from studies included in this analysis

Authors, country	Population	Strategy	Cost	Outcome in QALYs	ICER in cost per QALY*	Probability of cost-effectiveness (threshold in cost per QALY)
**Enhanced care pathway**						
Brunenberg, *et al.*,[3] Netherlands	THA	Conventional care	USD 11 312	0.65		
		Joint Recovery Programme (pre- assessment and intensive rehabilitation)	USD 10 051	0.70		
		Increment	−1261	0.07†	Dominates	0.94 (USD 45 000)
	TKA	Conventional care	USD 12 877	0.61		
		Joint Recovery Programme (pre-operative assessment and intensive rehabilitation)	USD 8541	0.65		
		Increment	−3336	0.04†	Dominates	0.99 (USD 45 000)
Larsen, *et al.*,[4] Denmark	THA + TKA	Conventional care	DKK 90 227	0.78		
		Accelerated perioperative care and rehabilitation	DKK 71 344	0.83		
		Increment	−18 880	0.05	Dominates	0.97‡ (DKK 160 000)
	THA	Conventional care	DKK 87 657	0.75		
		Accelerated perioperative care and rehabilitation	DKK 71 768	0.84		
		Increment	−15 889	0.09	Dominates	0.98‡ (DKK 160 000)
	TKA	Accelerated perioperative care and rehabilitation	DKK 70 644	0.81		
		Conventional care	DKK 95 367	0.85		
		Increment	24 723	0.04	DKK 618 075	NR
**Preoperative**						
Assessment and optimization of comorbidities						
McLawhorn, *et al.*,[6] US	Morbidly obese with unilateral end-stage knee osteoarthritis					
		Immediate TKA	USD 60 453	10.83		
		Bariatric surgery, followed by TKA 2 years later	USD 84 099	12.53		
		Increment	USD 23 646	1.70	USD 13 910	0.988 (USD 100 000)
Staphylococcus aureus *prophylaxis*						
Courville, *et al.*,[27] US	THA	Standard infection prevention measures without *S. aureus* screening or mupirocin decolonization	USD 24 506	0.7980		

For peer review only - http://bmjopen.bmj.com/site/about/guidelines.xhtml

Supplemental Material: Enhanced recovery following hip and knee arthroplasty

Authors, country	Population	Strategy	Cost	Outcome in QALYs	ICER in cost per QALY*	Probability of cost-effectiveness (threshold in cost per QALY)
		Preoperative nasal screening for *S. aureus* followed by mupirocin treatment for patients with positive cultures	USD 24 471	0.7983	D	
		Empirical treatment of all preoperative patients with mupirocin	USD 24 258	0.7985		
		Increment (compared to standard measures)	−248	0.0005	Dominates	NR
	TKA	Standard infection prevention measures without *S. aureus* screening or mupirocin decolonization	USD 24 667	0.6783		
		Preoperative nasal screening for *S. aureus* followed by mupirocin treatment for patients with positive cultures	USD 24 611	0.6785	D	
		Empirical treatment of all preoperative patients with mupirocin	USD 24 378	0.6787		
		Increment (compared to standard measures)	−289	0.0004	Dominates	NR
**Intraoperative**						
Avoid unnecessary blood transfusion						
Jackson, *et al.*,[9] US	THA + TKA	Usual transfusion practice	NR	NR		
		Postoperative erythrocyte recovery and transfusion	NR	NR		
		Increment	USD 53	0.00001	USD 5 700 000	NR (USD 50 000)
Sonnenberg,[10] US	THA	Usual practice without autologous donation	USD 1395	NR		
		Autologous blood donation and transfusion	USD 1539	NR		
		Increment	144	0.0523	USD 2750	NR (USD 50 000)
Local infiltration of analgesia						
Marques, *et al.*,[22] UK	THA	Standard anaesthesia	NR	NR		
		Intraoperative local anaesthetic wound infiltration administered before wound closure in addition to standard anaesthesia	NR	NR		
		Increment	GBP −86	0.052	Dominates	0.98 (GBP 20 000)
	TKA	Standard anaesthesia	NR	NR		
		Intraoperative local anaesthetic wound infiltration administered before wound closure in addition to standard anaesthesia	NR	NR		

For peer review only - http://bmjopen.bmj.com/site/about/guidelines.xhtml

Supplemental Material: Enhanced recovery following hip and knee arthroplasty

Authors, country	Population	Strategy	Cost	Outcome in QALYs	ICER in cost per QALY*	Probability of cost-effectiveness (threshold in cost per QALY)
		Increment	GBP −77	0.009	Dominates	0.60 (GBP 20 000)
Infection prevention						
Cummins, *et al.*,[28] US	THA	Conventional cement	USD 24 100	9.439		
		Antibiotic-impregnated bone cement	USD 23 900	9.454		
		Increment	−200	0.015	Dominates	NR
Graves, *et al.*,[29] UK[§]	THA	No systemic antibiotics, plain cement and conventional ventilation	GBP 0[§]	0[§]		
		Systemic antibiotics, antibiotic-impregnated cement, laminar ventilation and body exhaust suit	GBP 781 075	62	D	0.01[‖] (GBP 18 000)
		No systemic antibiotics, antibiotic-impregnated cement and conventional ventilation	GBP −4 634 647	89	D	0.07[‖] (GBP 18 000)
		Systemic antibiotics, plain cement and conventional ventilation	GBP −7 226 732	101	D	0.15[‖] (GBP 18 000)
		Systemic antibiotics, antibiotic-impregnated cement, conventional ventilation and body exhaust suit	GBP −3 960 897	106	D	0.11[‖] (GBP 18 000)
		Systemic antibiotics, plain cement and laminar airflow	GBP −5 271 040	118	D	0.10[‖] (GBP 18 000)
		Systemic antibiotics, antibiotic-impregnated cement and laminar airflow	GBP −6 152 877	124	D	0.06[‖] (GBP 18 000)
		No systemic antibiotics, plain cement and laminar airflow	GBP −3 271 749	124	D	0.18[‖] (GBP 18 000)
		Systemic antibiotics, antibiotic-impregnated cement and conventional ventilation	GBP −8 325 277	147		
		Increment (compared to systemic antibiotics, plain cement and conventional ventilation)	−1 098 545	46	Dominates	0.32[e] (GBP 18 000)
Merollini, *et al.*,[30] Australia[¶]	THA	No antibiotic prophylaxis	AUD 1 517 954	−163.1		
		Antibiotic prophylaxis and laminar airflow	AUD 4 592 200	−126.9	D	
		Antibiotic prophylaxis	AUD 0[¶]	0[¶]	D	
		Antibiotic prophylaxis and antibiotic-impregnated cement	AUD −126 375	32.3		
		Increment	−126 375	32.3	Dominates	0.986 (AUD 40 000)[**]

For peer review only

For peer review only - http://bmjopen.bmj.com/site/about/guidelines.xhtml

Supplemental Material: Enhanced recovery following hip and knee arthroplasty

Authors, country	Population	Strategy	Cost	Outcome in QALYs	ICER in cost per QALY*	Probability of cost-effectiveness (threshold in cost per QALY)
**Postoperative**						
Physical therapy						
Fusco & Turchetti,[36] Italy	TKA, societal perspective	20 face-to-face rehabilitation sessions	EUR 1315	13.02		
		10 face-to-face rehabilitation sessions plus 10 telesessions	EUR 977	13.02		
		Increment	−338	0	Dominates	NR (EUR 30 000)
	TKA, Italian NHS perspective	20 face-to-face rehabilitation sessions	EUR 1124	13.02		
		10 face-to-face rehabilitation sessions plus 10 telesessions	EUR 862	13.02		
		Increment	−262	0	Dominates	NR (EUR 30 000)
Kauppila, *et al.*,[37] Finland	TKA	Multidisciplinary biopsychosocial outpatient rehabilitation programme	EUR 12 950	NR		
		Conventional orthopaedic care	EUR 11 120	NR		
		Increment	−1830	0.0192	Dominates	NR
Scheduling of follow-up						
Bolz, *et al.*,[46] Australia††	THA, assuming 5% revisions delayed under no follow-up‡‡					
		2-yearly routine follow-up	AUD 26 426 908	147 940		
		Follow-up at 3 months and 1 or 2 years	AUD 21 331 518	147 940	D	
		No follow-up	AUD 14 867 616	147 949		
		Increment	−6 463 902	9	Dominates	NR

* Strategies listed in order of increasing effectiveness; ICER presented compared to next most effective strategy that is not extended-dominated; no ICER presented for dominated or extended-dominated strategies; ICERs have been calculated where not explicitly presented in the reference, where ICERs have been presented they may differ slightly from what would be calculated from the presented figures due to rounding; † Adjusted for baseline quality of life using regression analysis
‡ Probability of being the dominant strategy; § Total costs and outcomes for a cohort of 77 321 patients, relative to the cost of the strategy of no systemic antibiotics, plain cement and conventional ventilation; ‖ Probability of being most cost-effective strategy in the model; ¶ Total costs and outcomes per 30 000 primary THRs, relative to the cost of the strategy of antibiotic prophylaxis; ** Cost-effectiveness ≥98.6% at typical willingness-to-pay values of AUD 40 000 to AUD 64 000; †† Total costs and outcomes for a cohort of 30 440 patients; ‡‡ Four different probabilities were modelled (1%, 5%, 10%, 50%), but the direction of the results did not change between them.
AUD, Australian dollar; D, dominated; DKK, Danish krone; EUR, euro; GBP, British pound; ICER, incremental cost-effectiveness ratio; NHS, National Health Service; NR, not reported; QALY, quality-adjusted life year; THA, total hip arthroplasty; TKA, total knee arthroplasty; UK, United Kingdom; US, United States; USD, United States dollar

For peer review only - http://bmjopen.bmj.com/site/about/guidelines.xhtml

Supplemental Material: Enhanced recovery following hip and knee arthroplasty

Table A9. Components of enhanced recovery pathway in the trials of an entire pathway

	Larsen, *et al.*[50]		Brunenberg, *et al.*[3]	
**Intervention type**	**Standard protocol**	**Accelerated protocol**	**Usual care**	**Joint recovery programme**
Pre-operative assessment	NA	NA	No standardized screening	History, examination & blood testing 6 weeks prior to surgery
Pre-operative education	Information given to patients on admission	Information provided in groups at outpatient clinic prior to hospitalization	No information session	Information session 1–2 weeks prior to surgery
Day of admission	Day before surgery	Day of surgery	NA	NA
Bed allocation in hospital	Amongst other patients	Patients having joint replacement placed together in separate part of ward	NA	NA
Staff involved	Various	One nurse in charge of multidisciplinary team of nurses, OT & PT	NA	Supervised by PT & nurses throughout admission
Nutrition	Screening	Screening plus daily intake 1.5 L fluid & 2 protein drinks	NA	NA
Start of mobilization	1 day after surgery	Day of surgery	NA	NA
Description of mobilization	Individual & gradual mobilization according to patient tolerance	Intensive mobilization in teams with pre-set goals	Conventional physiotherapy	Rehabilitation in groups in a room resembling the home situation
Duration of mobilization	4 hours daily	8 hours daily	1 hour daily	Duration not specified
Discharge planning	NA	NA	During admission	6 weeks prior to surgery
Other details	NA	NA		Patients could involve a family member or friend to give emotional support

NA, not applicable; OT, occupational therapists; PT, physiotherapists

For peer review only - http://bmjopen.bmj.com/site/about/guidelines.xhtml

Supplemental Material: Enhanced recovery following hip and knee arthroplasty

Table A10. Willingness to pay thresholds and incremental cost-effectiveness ratios for all studies converted into international dollars

Authors, country	Currency, cost year	Values as reported*		Adjusted values in international dollars†	
		WTP threshold	ICER (unit cost/QALY)	WTP threshold	ICER (unit cost/QALY)
Brunenberg, *et al.*, Netherlands[3]	USD, 2002	45 000	—‡	45 000	—‡
Larsen, *et al.*, Denmark[4]	DKK, 2006	160 000	618 075	22 122	99 624
McLawhorn, *et al.*, US[6]	USD, 2012	100 000	13 910	100 000	14 995
Courville, *et al.*, US[27]	USD, 2005	NR	—‡	—	—‡
Jackson, *et al.*, US[9]	USD, 2000§	50 000	5 700 000	50 000	7 787 912
Sonnenberg, US[10]	USD, 2000	50 000	2 750	50 000	3 757
Marques, *et al.*, UK[22]	GBP, 2015§	20 000	—‡	28 838	—‡
Cummins, *et al.*, US[28]	USD, 2002	NR	—‡	—	—‡
Graves, *et al.*, UK[29]	GBP, 2012	18 000	—‡	25 954	—‡
Merollini, *et al.*, Australia[30]	AUD, 2011	40 000	—‡	27 287	—‡
Fusco & Turchetti, Italy[36]	EUR, 2013	30 000	—‡	41 482	—‡
Kauppila, *et al.*, Finland[37]	EUR, 2006	NR	—‡	—	—‡
Bolz, *et al.*, Australia[46]	AUS, 2010§	NR	—‡	—	—‡

* Only non-dominating results reported
† Willingness to pay thresholds not adjusted for cost year as they have been constant in most countries since they were introduced, in spite of cost inflation; ICERs additionally adjusted to 2016 values
‡ Dominance reported for all strategies
§ Cost year not reported; assumed as publication year

AUD, Australian dollar; DKK, Danish krone; EUR, euro; GBP, British pound; ICER, incremental cost-effectiveness ratio; NR, not reported; QALY, quality-adjusted life year; UK, United Kingdom; US, United States; USD, United States dollar; WTP, willingness to pay

For peer review only - http://bmjopen.bmj.com/site/about/guidelines.xhtml

Supplemental Material: Enhanced recovery following hip and knee arthroplasty

References for supplementary material

1. Cooper N, Coyle D, Abrams K, et al. Use of evidence in decision models: an appraisal of health technology assessments in the UK since 1997. *J Health Serv Res Policy* 2005;10(4):245-50. doi: 10.1258/135581905774414187
2. Coyle D, Lee KM. Evidence-based economic evaluation: how the use of different data sources can impact results. In: Donaldson C, Mugford M, Vale L, eds. Evidence-based health economics: from effectiveness to efficiency in systematic review. London, UK: BMJ Publishing Group 2002:55-66.
3. Brunenberg DE, van Steyn MJ, Sluimer JC, et al. Joint recovery programme versus usual care: an economic evaluation of a clinical pathway for joint replacement surgery. *Medical care* 2005;43(10):1018-26. [published Online First: 2005/09/17]
4. Larsen K, Hansen TB, Thomsen PB, et al. Cost-effectiveness of accelerated perioperative care and rehabilitation after total hip and knee arthroplasty. *J Bone Joint Surg Am* 2009;91(4):761-72. doi: 10.2106/JBJS.G.01472
5. Köksal I, Tahta M, Şimşek ME, et al. Efficacy of rapid recovery protocol for total knee arthroplasty: a retrospective study. *Acta Orthop Traumatol Turc* 2015;49(4):382-6. doi: 10.3944/AOTT.2015.14.0353
6. McLawhorn AS, Southren D, Wang YC, et al. Cost-effectiveness of bariatric surgery prior to total knee arthroplasty in the morbidly obese: A computer model-based evaluation. *J Bone Joint Surg Am* 2016;98(2):e6. doi: 10.2106/JBJS.N.00416
7. Wang L, Lee M, Zhang Z, et al. Does preoperative rehabilitation for patients planning to undergo joint replacement surgery improve outcomes? A systematic review and meta-analysis of randomised controlled trials. *BMJ Open* 2016;6(2):e009857. doi: 10.1136/bmjopen-2015-009857
8. McGregor AH, Rylands H, Owen A, et al. Does preoperative hip rehabilitation advice improve recovery and patient satisfaction. *J Arthroplast* 2004;19(4):464-68.
9. Jackson BR, Umlas J, AuBuchon JP. The cost-effectiveness of postoperative recovery of RBCs in preventing transfusion-associated virus transmission after joint arthroplasty. *Transfusion* 2000;40(9):1063-6.
10. Sonnenberg FA. A health economic analysis of autologous transfusion. *Infus Ther Transfus Med* 2002;29(3):175-83.
11. Green WS, Toy P, Bozic KJ. Cost minimization analysis of preoperative erythropoietin vs autologous and allogeneic blood donation in total joint arthroplasty. *J Arthroplast* 2010;25(1):93-6. doi: 10.1016/j.arth.2008.10.005
12. Bedair H, Yang J, Dwyer MK, et al. Preoperative erythropoietin alpha reduces postoperative transfusions in THA and TKA but may not be cost-effective. *Clin Orthop Relat Res* 2015;473(2):590-6. doi: 10.1007/s11999-014-3819-z
13. Zhao Y, Jiang C, Peng H, et al. The effectiveness and safety of preoperative use of erythropoietin in patients scheduled for total hip or knee arthroplasty: A systematic review and meta-analysis of randomized controlled trials. *Medicine (Baltimore)* 2016;95(27):e4122. doi: 10.1097/MD.0000000000004122
14. Billote DB, Glisson SN, Green D, et al. A prospective, randomized study of preoperative autologous donation for hip replacement surgery. *J Bone Joint Surg Am* 2002;84-A(8):1299-304.
15. Marcinkowski K, McDonald B. Changing blood transfusion practice in elective joint arthroplasty: a nursing initiative. *Nurs Prax N Z* 2006;22(3):15-21.
16. Boese C, Gallo T, Weis M, et al. Efficacy and cost-effectiveness of a blood salvage system in primary total knee arthroplasty: A retrospective match-controlled chart review. *Iowa Orthop J* 2011; 31(2). http://onlinelibrary.wiley.com/o/cochrane/cleed/articles/NHSEED-22012012669/frame.html.
17. Muñoz M, Ariza D, Campos A, et al. The cost of post-operative shed blood salvage after total

For peer review only - http://bmjopen.bmj.com/site/about/guidelines.xhtml

Supplemental Material: Enhanced recovery following hip and knee arthroplasty

knee arthroplasty: an analysis of 1,093 consecutive procedures. *Blood Transfus* 2013;11(2):260-71. doi: 10.2450/2012.0139-12

18. Klika AK, Small TJ, Saleh A, et al. Primary total knee arthroplasty allogenic transfusion trends, length of stay, and complications: Nationwide inpatient sample 2000-2009. *J Arthroplast* 2014;29(11):2070-77. doi: 10.1016/j.arth.2014.06.018
19. Chen WH, Hung KC, Tan PH, et al. Neuraxial anesthesia improves long-term survival after total joint replacement: a retrospective nationwide population-based study in Taiwan. *Can J Anaesth* 2015;62(4):369-76. doi: 10.1007/s12630-015-0316-0
20. Demeere JL, Merckx C, Demeere N. Cost minimisation and cost effectiveness in anaesthesia for total hip replacement surgery, in Belgium? A study comparing three general anaesthesia techniques. *Acta Anaesthesiol Belg* 2006; 57(2). http://onlinelibrary.wiley.com/o/cochrane/cleed/articles/NHSEED-22006001662/frame.html.
21. Ilfeld BM, Mariano ER, Williams BA, et al. Hospitalization costs of total knee arthroplasty with a continuous femoral nerve block provided only in the hospital versus on an ambulatory basis: a retrospective, case-control, cost-minimization analysis. *Reg Anesth Pain Med* 2007;32(1):46-54.
22. Marques EMR, Blom AW, Lenguerrand E, et al. Local anaesthetic wound infiltration in addition to standard anaesthetic regimen in total hip and knee replacement: Long-term cost-effectiveness analyses alongside the APEX randomised controlled trials. *BMC Med* 2015;13:151. doi: 10.1186/s12916-015-0389-1
23. Panchmatia JR, Chegini S, Lobban C, et al. The routine use of tranexamic acid in hip and knee replacements. *Bull NYU Hosp Jt Dis* 2012;70(4):246-9.
24. Slover J, Bosco J. Cost analysis of use of tranexamic acid to prevent major bleeding complications in hip and knee arthroplasty surgery. *Am J Orthop (Belle Mead NJ)* 2014;43(10):E217-20.
25. Tuttle JR, Ritterman SA, Cassidy DB, et al. Cost benefit analysis of topical tranexamic acid in primary total hip and knee arthroplasty. *J Arthroplast* 2014;29(8):1512-5. doi: 10.1016/j.arth.2014.01.031
26. Chimento GF, Huff T, Ochsner JL, Jr., et al. An evaluation of the use of topical tranexamic acid in total knee arthroplasty. *J Arthroplast* 2013;28(8 Suppl):74-7. doi: 10.1016/j.arth.2013.06.037
27. Courville XF, Tomek IM, Kirkland KB, et al. Cost-effectiveness of preoperative nasal mupirocin treatment in preventing surgical site infection in patients undergoing total hip and knee arthroplasty: a cost-effectiveness analysis. *Infect Control Hosp Epidemiol* 2012;33(2):152-59. doi: 10.1086/663704
28. Cummins JS, Tomek IM, Kantor SR, et al. Cost-effectiveness of antibiotic-impregnated bone cement used in primary total hip arthroplasty. *J Bone Joint Surg Am* 2009;91(3):634-41. doi: 10.2106/JBJS.G.01029
29. Graves N, Wloch C, Wilson J, et al. A cost-effectiveness modelling study of strategies to reduce risk of infection following primary hip replacement based on a systematic review. *Health Technol Assess* 2016;20(54):1-144. doi: 10.3310/hta20540
30. Merollini KM, Crawford RW, Whitehouse SL, et al. Surgical site infection prevention following total hip arthroplasty in Australia: a cost-effectiveness analysis. *Am J Infect Control* 2013;41(9):803-9. doi: 10.1016/j.ajic.2012.11.015
31. Slover J, Haas JP, Quirno M, et al. Cost-effectiveness of a *Staphylococcus aureus* screening and decolonization program for high-risk orthopedic patients. *J Arthroplast* 2011; 26(3). http://onlinelibrary.wiley.com/o/cochrane/cleed/articles/NHSEED-22011000750/frame.html.
32. Meda M, Gentry V, Perry A, et al. Is preoperative screening for meticillin-susceptible *Staphylococcus aureus* in joint replacement surgery cost-effective to reduce surgical site infections? *J Hosp Infect* 2016;94(3):305-06. doi: 10.1016/j.jhin.2016.06.001
33. Elliott RA, Weatherly HLA, Hawkins NS, et al. An economic model for the prevention of MRSA

For peer review only - http://bmjopen.bmj.com/site/about/guidelines.xhtml

Supplemental Material: Enhanced recovery following hip and knee arthroplasty

infections after surgery: Non-glycopeptide or glycopeptide antibiotic prophylaxis? *Eur J Health Econ* 2010;11(1):57-66. doi: 10.1007/s10198-009-0175-0

34. Romanò CL, Romanò D, Logoluso N, et al. Septic versus aseptic hip revision: How different? *J Orthop Traumato* 2010;11(3):167-74. doi: 10.1007/s10195-010-0106-y
35. Chang P, Okamoto M, Chen J, et al. Cost-effectiveness analysis of ondansetron and prochlorperazine for the prevention of postoperative nausea and vomiting. *J Manag Care Pharm* 2005;11(4):317-21. doi: 10.18553/jmcp.2005.11.4.317
36. Fusco F, Turchetti G. Telerehabilitation after total knee replacement in Italy: cost-effectiveness and cost-utility analysis of a mixed telerehabilitation-standard rehabilitation programme compared with usual care. *BMJ Open* 2016;6(5):e009964. doi: 10.1136/bmjopen-2015-009964
37. Kauppila AM, Sintonen H, Aronen P, et al. Economic evaluation of multidisciplinary rehabilitation after primary total knee arthroplasty based on a randomized controlled trial. *Arthritis Care Res* 2011;63(3):335-41. doi: 10.1002/acr.20398
38. Iyengar KP, Nadkarni JB, Ivanovic N, et al. Targeted early rehabilitation at home after total hip and knee joint replacement: Does it work? *Disabil Rehabil* 2007;29(6):495-502. doi: 10.1080/09638280600841471
39. Mahomed NN, Davis AM, Hawker G, et al. Inpatient compared with home-based rehabilitation following primary unilateral total hip or knee replacement: A randomized controlled trial. *J Bone Joint Surg Am* 2008;90(8):1673-80. doi: 10.2106/JBJS.G.01108
40. Pengas IP, Khan WS, Bennett CA, et al. Impact of weekend physiotherapy service on the cost effectiveness of elective orthopaedic hip and knee arthroplasty. *Open Orthop J* 2015;9:515-9. doi: 10.2174/1874325001509010515
41. Krummenauer F, Günther KP, Witzleb WC. The incremental cost effectiveness of in-patient versus out-patient rehabilitation after total hip arthroplasty - Results of a pilot investigation. *Eur J Med Res* 2008;13(6):267-74.
42. Sigurdsson E, Siggeirsdottir K, Jonsson H, Jr., et al. Early discharge and home intervention reduces unit costs after total hip replacement: Results of a cost analysis in a randomized study. *Int J Health Care Finance Econ* 2008;8(3):181-92. doi: 10.1007/s10754-008-9036-0
43. Mitchell C, Walker J, Walters S, et al. Costs and effectiveness of pre- and post-operative home physiotherapy for total knee replacement: Randomized controlled trial. *J Eval Clin Pract* 2005; 11(3). http://onlinelibrary.wiley.com/o/cochrane/cleed/articles/NHSEED-22005000951/frame.html
http://onlinelibrary.wiley.com/store/10.1111/j.1365-2753.2005.00535.x/asset/j.1365-2753.2005.00535.x.pdf?v=1&t=j5wd9454&s=eca2eea1d978216556aa04bb5df99d1c0d1e7c08.
44. Joshi RN, White PB, Murray-Weir M, et al. Prospective randomized trial of the efficacy of continuous passive motion post total knee arthroplasty: Experience of the Hospital for Special Surgery. *J Arthroplast* 2015;30(12):2364-9. doi: 10.1016/j.arth.2015.06.006
45. Hunt GR, Crealey G, Murthy BV, et al. The consequences of early discharge after hip arthroplasty for patient outcomes and health care costs: Comparison of three centres with differing durations of stay. *Clin Rehabil* 2009;23(12):1067-77. doi: 10.1177/0269215509339000
46. Bolz KMD, Crawford RW, Donnelly B, et al. The cost-effectiveness of routine follow-up after primary total hip arthroplasty. *J Arthroplast* 2010;25(2):191-96. doi: 10.1016/j.arth.2008.12.009
47. Marsh J, Hoch JS, Bryant D, et al. Economic evaluation of web-based compared with in-person follow-up after total joint arthroplasty. *J Bone Joint Surg Am* 2014;96(22):1910-6. doi: 10.2106/JBJS.M.01558
48. Glaser D, Lotke P. Cost-effectiveness of immediate postoperative radiographs after uncomplicated total knee arthroplasty: A retrospective and prospective study of 750 patients. *J Arthroplast* 2000;15(4):475-78. doi: 10.1054/arth.2000.4338

For peer review only - http://bmjopen.bmj.com/site/about/guidelines.xhtml

Supplemental Material: Enhanced recovery following hip and knee arthroplasty

49. Manoharan V, Grant AL, Harris AC, et al. Closed incision negative pressure wound therapy vs conventional dry dressings after primary knee arthroplasty: A randomized controlled study. *J Arthroplast* 2016;31(11):2487-94. doi: 10.1016/j.arth.2016.04.016
50. Larsen K, Sørensen OG, Hansen TB, et al. Accelerated perioperative care and rehabilitation intervention for hip and knee replacement is effective: a randomized clinical trial involving 87 patients with 3 months of follow-up. *Acta Orthop* 2008;79(2):149-59. doi: 10.1080/17453670710014923 [published Online First: 2008/05/15]
51. Wylde V, Lenguerrand E, Gooberman-Hill R, et al. Effect of local anaesthetic infiltration on chronic postsurgical pain after total hip and knee replacement: the APEX randomised controlled trials. *Pain* 2015;156(6):1161-70. doi: 10.1097/j.pain.0000000000000114

For peer review only

For peer review only - http://bmjopen.bmj.com/site/about/guidelines.xhtml

PRISMA 2009 Checklist

Section/topic	#	Checklist item	Reported on page #
**TITLE**			
Title	1	Identify the report as a systematic review, meta-analysis, or both.	1
**ABSTRACT**			
Structured summary	2	Provide a structured summary including, as applicable: background; objectives; data sources; study eligibility criteria, participants, and interventions; study appraisal and synthesis methods; results; limitations; conclusions and implications of key findings; systematic review registration number.	2
**INTRODUCTION**			
Rationale	3	Describe the rationale for the review in the context of what is already known.	5
Objectives	4	Provide an explicit statement of questions being addressed with reference to participants, interventions, comparisons, outcomes, and study design (PICOS).	5
**METHODS**			
Protocol and registration	5	Indicate if a review protocol exists, if and where it can be accessed (e.g., Web address), and, if available, provide registration information including registration number.	6
Eligibility criteria	6	Specify study characteristics (e.g., PICOS, length of follow-up) and report characteristics (e.g., years considered, language, publication status) used as criteria for eligibility, giving rationale.	6
Information sources	7	Describe all information sources (e.g., databases with dates of coverage, contact with study authors to identify additional studies) in the search and date last searched.	6
Search	8	Present full electronic search strategy for at least one database, including any limits used, such that it could be repeated.	6 and Supplementary Table A1
Study selection	9	State the process for selecting studies (i.e., screening, eligibility, included in systematic review, and, if applicable, included in the meta-analysis).	6
Data collection process	10	Describe method of data extraction from reports (e.g., piloted forms, independently, in duplicate) and any processes for obtaining and confirming data from investigators.	6
Data items	11	List and define all variables for which data were sought (e.g., PICOS, funding sources) and any assumptions and simplifications made.	6-7
Risk of bias in individual studies	12	Describe methods used for assessing risk of bias of individual studies (including specification of whether this was done at the study or outcome level), and how this information is to be used in any data synthesis.	7
Summary measures	13	State the principal summary measures (e.g., risk ratio, difference in means).	7
Synthesis of results	14	Describe the methods of handling data and combining results of studies, if done, including measures of consistency (e.g., I^2) for each meta-analysis.	7

For peer review only

PRISMA 2009 Checklist

Section/topic	#	Checklist item	Reported on page #
Risk of bias across studies	15	Specify any assessment of risk of bias that may affect the cumulative evidence (e.g., publication bias, selective reporting within studies).	7
Additional analyses	16	Describe methods of additional analyses (e.g., sensitivity or subgroup analyses, meta-regression), if done, indicating which were pre-specified.	Not applicable
**RESULTS**			
Study selection	17	Give numbers of studies screened, assessed for eligibility, and included in the review, with reasons for exclusions at each stage, ideally with a flow diagram.	9 and Figure 1
Study characteristics	18	For each study, present characteristics for which data were extracted (e.g., study size, PICOS, follow-up period) and provide the citations.	Table 1 and Supplementary Tables A4,A5 and A6
Risk of bias within studies	19	Present data on risk of bias of each study and, if available, any outcome level assessment (see item 12).	Figure 2 and Table 2
Results of individual studies	20	For all outcomes considered (benefits or harms), present, for each study: (a) simple summary data for each intervention group (b) effect estimates and confidence intervals, ideally with a forest plot.	Table 3 and Supplementary Table A7 and A9
Synthesis of results	21	Present results of each meta-analysis done, including confidence intervals and measures of consistency.	Not applicable
Risk of bias across studies	22	Present results of any assessment of risk of bias across studies (see Item 15).	11
Additional analysis	23	Give results of additional analyses, if done (e.g., sensitivity or subgroup analyses, meta-regression [see Item 16]).	Not applicable
**DISCUSSION**			
Summary of evidence	24	Summarize the main findings including the strength of evidence for each main outcome; consider their relevance to key groups (e.g., healthcare providers, users, and policy makers).	17-18
Limitations	25	Discuss limitations at study and outcome level (e.g., risk of bias), and at review-level (e.g., incomplete retrieval of identified research, reporting bias).	18-19
Conclusions	26	Provide a general interpretation of the results in the context of other evidence, and implications for future research.	19
**FUNDING**			
Funding	27	Describe sources of funding for the systematic review and other support (e.g., supply of data); role of funders for the systematic review.	4

For peer review only - http://bmjopen.bmj.com/site/about/guidelines.xhtml

PRISMA 2009 Checklist

From: Moher D, Liberati A, Tetzlaff J, Altman DG, The PRISMA Group (2009). Preferred Reporting Items for Systematic Reviews and Meta-Analyses: The PRISMA Statement. PLoS Med 6(7): e1000097. doi:10.1371/journal.pmed1000097

For more information, visit: **www.prisma-statement.org**. Page 2 of 2

For peer review only

BMJ Open

Enhanced recovery following hip and knee arthroplasty: A systematic review of cost-effectiveness evidence

Journal:	*BMJ Open*
Manuscript ID	bmjopen-2019-032204.R1
Article Type:	Original research
Date Submitted by the Author:	31-Oct-2019
Complete List of Authors:	Pritchard, Mark; University of Oxford, Nuffield Department of Population Health<br>Murphy, Jacqueline; Queen Mary University of London,<br>Cheng, Lok; University of Oxford, Nuffield Department of Population Health<br>Janarthanan, Roshni; University of Oxford, Nuffield Department of Population Health<br>Judge, Andrew; University of Bristol, Musculoskeletal Research Unit, Translational Health Sciences, Bristol Medical School, University of Bristol, Learning and Research Building, Level 1, Southmead Hospital, Southmead, BS10 5NB<br>Leal, Jose; University of Oxford, Nuffield Department of Population Health
**Primary Subject Heading**:	Health economics
Secondary Subject Heading:	Surgery
Keywords:	Systematic review, hip replacement, knee replacement, osteoarthritis, economic evaluation, cost-effectiveness

SCHOLARONE™
Manuscripts

Enhanced recovery following hip and knee arthroplasty: A systematic review of cost-effectiveness evidence

Mark G Pritchard, MB ChB, MSc,[1,2,*] markgpritchard@gmail.com, ORCID iD 0000-0003-1726-8989

Jacqueline Murphy, MMath,[1,*] jacqueline.murphy@ndph.ox.ac.uk, ORCID iD 0000-0002-3927-7002

Lok Yin Cheng, MBChB, BSc,[1,3] lok.cheng@nhs.net

Roshni Janarthanan MBBS,[1,2] roshni.janarthanan@medsci.ox.ac.uk

Andrew Judge, PhD,[4,5] andrew.judge@bristol.ac.uk, ORCID iD 0000-0003-3015-0432

José Leal, DPhil,[1] jose.leal@ndph.ox.ac.uk, ORCID iD 0000-0001-7870-6730

1 Health Economics Research Centre, Nuffield Department of Population Health, University of Oxford, Oxford, United Kingdom

2 Oxford University Hospitals NHS Foundation Trust, John Radcliffe Hospital, Oxford, United Kingdom

3 Stoke Mandeville Hospital, Buckinghamshire Healthcare NHS Trust, Aylesbury, Buckinghamshire, United Kingdom

4 Nuffield Department of Orthopaedics, Rheumatology and Musculoskeletal Sciences (NDORMS), Centre for Statistics in Medicine, University of Oxford, Oxford, United Kingdom

5 Musculoskeletal Research Unit, Translational Health Sciences, Bristol Medical School, University of Bristol, Bristol, United Kingdom

* JM and MGP contributed equally to this study.

Correspondence to: Dr José Leal, Health Economics Research Centre, Nuffield Department of Population Health, University of Oxford, Old Road Campus, Oxford OX3 7LF, United Kingdom.

Telephone: 01865 289 263. jose.leal@dph.ox.ac.uk

Word count: 4055

For peer review only - http://bmjopen.bmj.com/site/about/guidelines.xhtml

Structured Abstract

Background: Hip and knee arthroplasties are common procedures and numbers are increasing. Enhanced recovery pathways are designed to optimize preoperative, intraoperative and postoperative phases of care. Previous reviews have assessed effectiveness but not cost-effectiveness of pathways for total hip arthroplasty (THA) and total knee arthroplasty (TKA).

Objectives: To assess evidence from cost–utility analyses of enhanced recovery pathways for patients having THA or TKA for osteoarthritis, report on study quality, and identify research gaps for future work.

Design: Systematic review of English-language peer-reviewed papers published between January 2000 and August 2019, describing cost–utility analyses of enhanced recovery pathways, or components of one, compared to usual care, in patients having THA or TKA.

Results: We identified 17 studies: five trial-based and 12 model-based. Two cost–utility analyses evaluated the entire enhanced recovery pathways and reported these to be cost effective compared to usual care. Eleven pathway components were found to be more effective and cost-saving compared to usual care, two were found to be cost-effective, and two were not cost-effective. We had concerns around risk of bias for all included studies, particularly regarding the short time horizon of the trials and lack of reporting of model validation.

Conclusions: Consistent results were found supporting enhanced recovery pathways as a whole, prophylactic systemic antibiotics, antibiotic-impregnated cement and conventional ventilation for infection prevention. No other interventions were subject of more than one study. We found ample scope for future cost-effectiveness studies, in particular analyses of entire recovery pathways and comparison of incremental changes within pathways rather than considering aspects of care in isolation.

Registration: *International Prospective Register of Systematic Reviews* number CRD42017059473

Keywords: Arthroplasty, cost-effectiveness, cost–utility, enhanced recovery, hip, knee, systematic review

For peer review only - http://bmjopen.bmj.com/site/about/guidelines.xhtml

ARTICLE SUMMARY

Strengths and limitations of this study

- This systematic review of enhanced recovery pathways for hip and knee arthroplasties had a detailed search strategy, including entire pathways and their components.
- Appropriate tools were used to assess quality and validity of models, trials and economic evaluations.
- Conclusions were reliant on the availability, quality and validity of published studies into cost-effectiveness of hip and knee arthroplasty.

For peer review only

For peer review only - http://bmjopen.bmj.com/site/about/guidelines.xhtml

Funding statement

This project was funded by the National Institute for Health Research Health Services and Delivery Research Programme (project number 14/46/02). Support was received from the Oxford NIHR Biomedical Research Centre, Nuffield Orthopaedic Centre, University of Oxford. AJ was supported by the NIHR Biomedical Research Centre at the University Hospitals Bristol NHS Foundation Trust and the University of Bristol. The views and opinions expressed herein are those of the authors and do not necessarily reflect those of the HS&DR Programme, NIHR, NHS or the Department of Health and Social Care.

Competing interests statement

The authors declare they have no competing interests.

For peer review only

For peer review only - http://bmjopen.bmj.com/site/about/guidelines.xhtml

Introduction

Hip and knee arthroplasties are common procedures: around a million of each were performed in 2011 within Organisation for Economic Co-operation and Development (OECD) countries,[1 2] and rates continue to increase.[3-6] Enhanced recovery is a multimodal approach to reduce surgical morbidity and mortality. Recognizing that factors other than surgical technique affect patient outcomes, Kehlet[7] considered how to optimize the preoperative, intraoperative and postoperative phases of patient care. These principles have been further developed specifically within the context of hip and knee arthroplasty.[8-15] Common components of an enhanced recovery pathway for hip and knee arthroplasty are listed in Box 1. A recent systematic review[16] found that enhanced recovery after hip and knee arthroplasties reduced length of stay in hospital. The authors cited a study from New Zealand[17] that found an enhanced recovery pathway to be cost saving, but the study did not include any data on cost-effectiveness.

Cost–utility analyses have become the preferred approach to inform decisions on healthcare resource allocation.[18 19] In these, the effects of treatments are measured in quality-adjusted life years (QALY): the product of health-related quality of life (anchored at 0 for death and 1 for perfect health), and the time (in years) spent experiencing that level of health. Incremental cost-effectiveness ratios (ICER) are used to compare a treatment to a less effective alternative and a threshold value used to determine whether it is cost-effective. Different countries have different thresholds for how much they are willing to pay per QALY gained.[20]

Systematic reviews of cost-effectiveness analyses have considered arthroplasty *vs* conservative management,[21 22] and specific components of enhanced recovery such as thromboprophylaxis.[23-25] However, we are not aware of any systematic reviews investigating the cost-effectiveness of a complete enhanced recovery pathway, or of most of the components. Our aim was to assess the cost-effectiveness evidence of enhanced recovery for patients having hip and knee arthroplasty. Specifically, we were interested in studies of adults having total hip arthroplasty (THA) or total knee arthroplasty (TKA) for osteoarthritis, comparing an enhanced recovery pathway

For peer review only - http://bmjopen.bmj.com/site/about/guidelines.xhtml

or components of one against usual care. Secondarily, we wanted to report study quality and identify research gaps for future work.

Methods

The complete methods are available in the published protocol,[26] registered with the International Prospective Register of Systematic Reviews, number CRD42017059473.[27] The selection of electronic databases and the search strategy were developed with an information specialist and in line with the preferred reporting items for systematic reviews and meta-analyses (PRISMA).[28] We searched Ovid MEDLINE, Embase, the National Health Service Economic Evaluations Database (via the Cochrane Library), and EconLit (via ProQuest), for English-language peer-reviewed papers published between 1 January 2000 and 1 March 2017 that included a cost–utility analysis of an enhanced recovery pathway or components of one, compared to usual care in patients having hip or knee arthroplasties (the complete search strategy for each database is presented in the Supplementary Table A1). Additional publications meeting our inclusion criteria were identified from the reference lists of the included studies. We repeated our search in August 2019 to identify more recently published studies. Our target population was adults having surgery for osteoarthritis. Studies exclusively concerning populations with other indications for surgery were excluded. We included studies with patients having different indications for surgery if the majority had osteoarthritis, or if the presented results allowed independent extraction of data for the subpopulation with osteoarthritis. Given that osteoarthritis is an indication for 92% of hip and 96% of knee arthroplasties,[29] we assumed that studies not providing details of the indication for surgery were representative of a population with osteoarthritis, and therefore included these studies. Evaluations of surgical technique or choice of implant were excluded.

Studies were independently screened based on their titles and abstracts by four reviewers (MGP, JM, LYC and JL). Full texts were obtained for studies chosen for inclusion by any reviewer. As an amendment to the protocol, evaluations of thromboprophylaxis were excluded at the full-text

For peer review only - http://bmjopen.bmj.com/site/about/guidelines.xhtml

stage due to a recent comprehensive systematic review in that area.[23] Data extraction was performed for remaining studies by three reviewers (MGP, JM and RJ), with disagreements resolved by a fourth reviewer (JL). The data extraction proforma is included in the published protocol.[26]

To assess the quality of studies, we completed the Consensus on Health Economic Criteria (CHEC) list[30] for each publication. For model-based studies, we also used the questionnaire produced by the International Society for Pharmacoeconomics and Outcomes Research (ISPOR),[31] and the Assessment of the Validation Status of Health-Economic (AdViSHE) decision models tool.[32] We added the Cochrane Collaboration's tool[33] to the original protocol to assess risk of bias in trial-based studies, referring to the original reports of trial outcomes where necessary. We assessed the quality of the data sources used in the studies according to a pre-specified hierarchy of evidence tool (see Supplementary Table A2).[34 35] For each component of a study, a score of 1 represented the use of the most appropriate data source, with increasing numbers representing progressively less appropriate sources.

The principal outcomes were a point estimate of cost-effectiveness in terms of incremental cost per QALY gained, and the probability of an intervention being cost effective according to the willingness to pay threshold used by the authors of each study. We also examined whether an intervention could be cost-effective in a different country or setting than the original study. Hence, we assumed the studies to be generalizable and the ICERs to be comparable after conversion into 2016 purchasing power parity international dollars (US PPP).[36] As the studies had different methodologies and looked at different combinations of components of the enhanced recovery pathway, no quantitative synthesis of the study results was attempted.

For peer review only - http://bmjopen.bmj.com/site/about/guidelines.xhtml

Box 1. Suggested components of an enhanced recovery pathway for hip and knee arthroplasty patients, data from references.[8-15]

Preoperative

- Education
- Discharge planning
- Multidisciplinary assessment
- Neuromuscular electrical stimulation
- Nutrition screening
- Optimization of comorbidities
- Physiotherapy
- Premedication (possibly including standardized analgesia and steroids)
- Pulsed electromagnetic fields

Intraoperative

- Standardized intravenous fluids
- Avoid unnecessary blood transfusion
- Minimally invasive surgery
- Reduce heat loss
- Specified anaesthetic requirements, such as spinal anaesthesia
- Local infiltration of anaesthesia
- Tranexamic acid
- Prophylactic antibiotics
- Intravenous dexamethasone
- Avoid unnecessary drains
- Computer-assisted surgery

Postoperative

- Analgesia
    - Continuous neural block
    - Standardized multimodal analgesia, scheduled and as required
    - Reduce opioid use / avoid patient-controlled intravenous opioid analgesia
- Scheduled anti-emetic
- Physical therapy starting on day of surgery
- Avoid unnecessary blood transfusion
- Oxygen administration
- Avoid sleep disturbances
- Early oral nutrition
- Wound care

For peer review only - http://bmjopen.bmj.com/site/about/guidelines.xhtml

Thromboprophylaxis<br>Neuromuscular electrical stimulation<br>Aim for early discharge

Patient and Public Involvement

No Patient involved.

Results

Study selection

Our original search identified 11 060 publications, and one additional publication was found from other sources. We repeated the search in August 2019 and identified 3903 additional studies. After excluding duplicates, we screened 8657 titles and abstracts. We excluded 8482 papers based on their abstracts. For one study, we were unable to obtain the full text but this did not appear to be a cost-effectiveness study from the abstract. We therefore reviewed 174 full texts. We excluded 157 studies following review of their full texts (reasons for exclusion given in Figure 1), and included seventeen papers in this review.[37-53]

Overview of included studies

Table 1 summarises included studies. Eight studies included both THA and TKA, [37 38 40-43 45 49] five only THA,[44 46-48 53] and four only TKA.[39 50-52] Two papers looked at an entire enhanced recovery pathway.[37 38] We identified cost–utility evaluations of optimization of comorbidities (specifically morbid obesity),[39] measures to reduce allogenic blood transfusion,[42-44] local infiltration of anaesthetic,[45] prophylactic antibiotics and other infection prevention measures,[41 46-49] and physical therapy before[40] or after surgery. [40 50-52] The final study[53] concerned the optimal timing of follow-up which, although not included in our prior list of enhanced recovery interventions, we have included here as being allied to the pathway. Five studies were trial-based economic evaluations,[37 38 40 45 51] the

For peer review only - http://bmjopen.bmj.com/site/about/guidelines.xhtml

remaining twelve being model-based. Eight studies were from Europe,[37 38 40 45 47 49-51] seven from the United States,[39 41-44 46 52] and two from Australia.[48 53] Perspective, time horizon, discount rates and price year used in each study are reported in Supplementary Table A3. The study perspective varied from only hospital costs [42-44 46 48 52 53] to a broader (societal) cost perspective.[37-41 49 50] Types of costs captured in each study are reported in Supplementary Table A4. Of the seven studies eliciting utilities, QALYs were informed by utility values based on the EQ-5D-3L instrument in six [37 40 45 50 52 54] and on the 15D instrument in one (Supplementary Table A5).[51]

For peer review only

For peer review only - http://bmjopen.bmj.com/site/about/guidelines.xhtml

Table 1. Summary of studies included in this analysis

Authors, publication year	Comparison	Joint	Study type	Country
**Enhanced recovery pathway**				
Brunenberg, *et al.* 2005[37]	Joint Recovery Programme (pre- assessment and intensive rehabilitation), *vs* conventional care	Hip and knee	Trial-based	Netherlands
Larsen, *et al.*, 2009[38]	Accelerated perioperative care and rehabilitation, *vs* conventional care	Hip and knee	Trial-based	Denmark
**Preoperative**				
McLawhorn, *et al.*, 2016[39]	Bariatric surgery followed by TKA 2 years later, *vs* immediate TKA	Knee	Markov model	US
Fernandes, *et al.*, 2017[40]	Supervised neuromuscular exercise and an educational package, *vs* educational package alone	Hip and knee	Trial-based	Denmark
Courville, *et al.*, 2012[41]	Preoperative nasal screening for *S. aureus* colonization followed by mupirocin treatment for patients with positive cultures, *vs* empirical treatment of all preoperative patients with mupirocin, *vs* standard infection prevention measures without *S. aureus* screening or mupirocin decolonization.	Hip and knee	Decision tree model	US
**Intraoperative**				
Jackson, *et al.*, 2000[42]	Postoperative erythrocyte recovery and transfusion, *vs* usual transfusion practice	Hip and knee	Markov model	US
Ramkumar, *et al.*, 2018[43]	Single dose intravenous tranexamic acid, *vs* single dose intravenous aminocaproic acid, *vs* no pharmacologic haemostatic agent	Hip and knee	Decision-tree model	US
Sonnenberg, 2002[44]	Autologous blood donation and transfusion, *vs* usual practice without autologous donation	Hip	Markov model	US
Marques, *et al.*, 2015[45]	Intraoperative local anaesthetic wound infiltration administered before wound closure in addition to standard anaesthesia, *vs* standard anaesthesia	Hip and knee	Trial-based	UK
Cummins, *et al.*, 2009[46]	Antibiotic-impregnated bone cement, *vs* conventional cement	Hip	Markov model	US
Graves, *et al.*, 2016[47]	Nine arms, comparing combinations of prophylactic systemic antibiotics, antibiotic-impregnated cement, laminar airflow and body exhaust suits	Hip	Markov model	UK
Merollini, *et al.*, 2013[48]	No antibiotic prophylaxis, antibiotic prophylaxis and antibiotic-impregnated cement, and antibiotic prophylaxis and laminar airflow, each compared to a baseline strategy of routine antibiotic prophylaxis	Hip	Markov model	Australia
Nherera, *et al.*, 2017[49]	Single-use negative pressure wound therapy dressings, *vs* usual care	Hip and knee	Decision-tree model	UK
**Postoperative**				
Fusco & Turchetti, 2016[50]	10 face-to-face rehabilitation sessions plus 10 telesessions, *vs* 20 face-to-face rehabilitation sessions	Knee	Markov model	Italy
Kauppila, *et al.*, 2011[51]	Multidisciplinary biopsychosocial outpatient rehabilitation programme, *vs* conventional orthopaedic care	Knee	Trial-based	Finland

For peer review only

Authors, publication year	Comparison	Joint	Study type	Country
Smith, *et al.*, 2018[52]	Telephonic health coaching and financial incentives, vs telephone calls conveying general health messages	Knee	Markov model	US
Bolz, *et al.*, 2010[53]	2-yearly routine follow-up, *vs* follow-up at 3 months and 1 or 2 years, *vs* no follow-up	Hip	Markov model	Australia

NHS: National Health Service; THA: total hip arthroplasty; TJA: total joint arthroplasty; TKA: total knee arthroplasty; UKA: unicompartmental knee arthroplasty

For peer review only

For peer review only - http://bmjopen.bmj.com/site/about/guidelines.xhtml

Assessment of study and reporting quality

Using the CHEC list, the quality of the studies was generally good (Figure 2a). Items raising more concerns were the short time horizons of the five trial-based studies (one year)[37 38 40 45 51] and three model-based studies (between six weeks[49] and seven years[53]). Using the Cochrane Collaboration's tool (Figure 2b) for the five trials[37 54-57] studies were based on, the risk of bias was low for items such as incomplete outcome data and selective reporting but high or uncertain for the remainder. Four trial reports stated that participants were allocated at random,[54-57] and the fifth was a before and after trial whereby patients were recruited consecutively from a waiting list.[37] Larsen and colleagues[54] discussed stratification, Kauppila and colleagues[55] reported a computer-generated sequence, and Villadsen and colleagues[56] used sequentially numbered sealed opaque envelopes. The staff administering the interventions were not blinded to allocation in any trial, and in three trials,[37 55 57] outcomes were assessed by researchers aware of the treatment allocation. Using the ISPOR questionnaire, the quality of the model-based studies was generally good (Figure 2c). However, none of the model-based studies reported a detailed process for internal and external validation. Four studies[42 44 50 52] were based on previously published models.[58-61] Of these, only Briggs and colleagues[58] (the basis for Fusco and Turchetti's model[50]) provided details of model validation. Some model-based studies excluded potential important outcomes, for example, Cummins and colleagues[46] did not include the possibility of requiring more than one revision surgery, and in Bolz and colleagues' model[53] there was no reduction in utility associated with delayed revisions. Further limitations in model validation were highlighted with the AdViSHE tool (Figure 2d).[32]

The hierarchy of evidence used in the studies is reported in Supplementary Table A6. Three model-based studies[42 44 46] scored poorly as they did not use randomised trial data to inform the clinical effective size of the interventions being analysed. Most studies did not cite a source for the assumption of the duration of treatment effect beyond that observed in the primary source for clinical effect size. Four trial-based studies and one model-based study included assessments of

For peer review only - http://bmjopen.bmj.com/site/about/guidelines.xhtml

utilities from patients using validated tools and scored highly in terms of quality of evidence.

Results of economic evaluation

The results of the included studies are summarized in Table 2. Costs, QALYs and incremental cost-effectiveness ratios for each comparison performed in each study are listed in Supplementary Table A7.

For peer review only

Table 2. Summary of findings from studies included in this analysis

Authors, country	Population	Strategy	Cost-effective?
**Enhanced recovery pathway**			
Brunenberg, *et al.*[37] Netherlands	THA and TKA	Conventional care	-
		Joint Recovery Programme (pre- assessment and intensive rehabilitation)	Yes, more effective and less costly
Larsen, *et al.*,[38] Denmark	THA	Conventional care	-
		Accelerated perioperative care and rehabilitation	Yes, more effective and less costly
	TKA	Conventional care	-
		Accelerated perioperative care and rehabilitation	Yes, less effective but less costly
**Preoperative**			
McLawhorn, *et al.*,[39] US	Morbid obese TKA	Immediate TKA	-
		Bariatric surgery, followed by TKA 2 years later	Yes
Fernandes, *et al.*[40] Denmark	THA and TKA	Educational package	-
		Supervised neuromuscular exercise in addition to educational package	Yes, more effective and less costly
Courville, *et al.*,[41] US	THA and TKA	Standard infection prevention measures without *S. aureus* screening or mupirocin decolonization OR preoperative nasal screening for *S. aureus* followed by mupirocin treatment for patients with positive cultures	-
		Empirical treatment of all preoperative patients with mupirocin	Yes, more effective and less costly
**Intraoperative**			
Jackson, *et al.*,[42] US	THA and TKA	Usual transfusion practice	-
		Postoperative erythrocyte recovery and transfusion	No
Ramkumar, *et al.*,[43] US	THA and TKA	No pharmacologic haemostatic agent OR single dose intravenous aminocaproic acid	-
		Single dose intravenous tranexamic acid	Yes, more effective and less costly
Sonnenberg,[44] US	THA	Usual practice without autologous donation	-
		Autologous blood donation and transfusion	Yes
Marques, *et al.*,[45] UK	THA and TKA	Standard anaesthesia	-
		Intraoperative local anaesthetic wound infiltration administered before wound closure in addition to standard anaesthesia	Yes, more effective and less costly
Cummins, *et al.*,[46] US	THA	Conventional cement	-
		Antibiotic-impregnated bone cement	Yes, more effective and less costly
Graves, *et al.*,[47] UK§	THA	No systemic antibiotics, plain cement and conventional ventilation	-
		Systemic antibiotics, antibiotic-impregnated cement and conventional ventilation	Yes, more effective and less costly
Merollini, *et al.*,[48] Australia	THA	No antibiotic prophylaxis OR antibiotic prophylaxis OR antibiotic prophylaxis and laminar airflow	-
		Antibiotic prophylaxis and antibiotic-impregnated cement	Yes, more effective and less costly
Nherera, *et al.*,[49] UK	THA and TKA	Usual care	-
		Single-use negative pressure wound therapy dressings	Yes, more effective and less costly
**Postoperative**			
Fusco &	TKA	20 face-to-face rehabilitation sessions	-

For peer review only

For peer review only - http://bmjopen.bmj.com/site/about/guidelines.xhtml

Authors, country	Population	Strategy	Cost-effective?
Turchetti,[50] Italy		10 face-to-face rehabilitation sessions plus 10 telesessions	Yes, same effectiveness but less costly
Kauppila, *et al.*,[51] Finland	TKA	Conventional orthopaedic care	-
		Multidisciplinary biopsychosocial outpatient rehabilitation programme	No
Smith, *et al.*,[52] US	TKA	Telephone calls conveying general health messages	-
		Telephonic health coaching and financial incentives to increase physical activity	Yes
Bolz, *et al.*,[53] Australia	THA	2-yearly routine follow-up OR follow-up at 3 months and 1 or 2 years	-
		No follow-up	Yes

Whole recovery pathway

Two studies considered multiple components of the enhanced recovery pathway. The first study was an economic analysis of a randomized trial with 56 THA and 31 TKA participants in Denmark.[38] The pathway used in the treatment arm ("accelerated care") had no difference from the control group ("conventional rehabilitation") in terms of intraoperative management, analgesia, nausea control or bowel regulation. Differences in the treatment protocols between the two arms involved patient education, nutrition, admission times, staffing, and mobilization (described in Supplementary Table A8). The accelerated care pathway was the dominant strategy both overall and in the subgroup of THA patients (i.e. less costly and more effective than the control group). For TKA patients, the authors found the accelerated care pathway to be cost-saving but less effective compared to the control group although not statistically significant. There was a cost saving of 618 075 Danish krone per QALY lost with accelerated care compared to conventional rehabilitation, which made it cost-effective (threshold of 160 000 krone per QALY in Denmark).

The second study was an economic evaluation of a before and after trial with 98 THA and 62 TKA participants in The Netherlands.[37] The intervention ('Joint Recovery Programme') consisted of a 20-minute pre-assessment screening 6 weeks before the operation for physical assessment and analysis of the home situation to aid discharge planning, patient education sessions one to two weeks before surgery; group rehabilitation sessions, and supervision by physical therapists and

For peer review only - http://bmjopen.bmj.com/site/about/guidelines.xhtml

nurses (Supplementary Table A7). Patients in the 'usual care' group underwent conventional physiotherapy for one hour daily, did not receive pre-assessment screening or information sessions, and discharge arrangements were addressed during admission to hospital. The joint recovery programme intervention was dominant for both hip and knee replacement, resulting in a cost saving of 1261 United States dollars (USD) per patient for THA and 3336 USD per patient for TKA, with no statistically significant difference in effect. The probability that the joint recovery programme was the most cost-effective option was above 80% for THA and TKA for willingness to pay thresholds up to 45 000 USD.

Preoperative components

McLawhorn and colleagues[39] used a Markov model to assess the cost-effectiveness of bariatric surgery two years before TKA for morbidly obese (body mass index ≥35 kg/m^2) patients who were candidates for both operations due to end-stage knee osteoarthritis and failed non-operative weight-loss interventions. The strategy including bariatric surgery was cost-effective at the stated willingness to pay threshold of 100 000 USD per QALY in 98.8% of probabilistic simulations.

Fernandes and colleagues[40] conducted an economic evaluation alongside a trial of 8 weeks of supervised neuromuscular exercise in addition to an educational package prior to surgery. Their point estimate was that the intervention was dominant, with a saving of 132 Euro and a benefit of 0.04 QALYs. It had an 84% probability of being cost effective at a willingness to pay threshold of 40 000 Euro per QALY.

Courville and colleagues[41] compared three strategies of screening for and treating *Staphylococcus aureus* colonization to prevent deep surgical site infections for TKA and THA. They found that decolonization of all preoperative patients with mupirocin, without testing for *S. aureus*, was the dominant strategy (cheaper and more effective) when compared to treating only patients testing positive for *S. aureus*, or no screening or decolonization for *S. aureus*.

For peer review only - http://bmjopen.bmj.com/site/about/guidelines.xhtml

Intraoperative components

Strategies to reduce allogenic blood transfusions were collection of autologous blood prior to surgery,[44] aseptic collection of wound drainage,[42] and use of aminocaproic acid or tranexamic acid to reduce bleeding.[43] Autologous blood collection was found to be cost-effective at 2750 USD per QALY gained, whereas wound drainage collection was not, costing 5.7 million USD per QALY gained. However, in the latter study, the only benefit of avoiding allogenic transfusion considered was a reduced risk of blood-borne virus infection.[42] In contrast, Sonnenberg's model for THA[44] found a minimal effect from the risk of blood-borne virus infection in either costs or outcomes: 99.6% of the increase in QALYs was due to a reduced risk of bacterial infection. When the risk of bacterial infection was removed from this model, the ICER increased to 2.5 million USD per QALY gained. Use of tranexamic acid was more effective and cost-saving compared to either aminocaproic acid or not using a haemostatic agent.[43]

Marques and colleagues conducted economic evaluations[45] alongside two randomized controlled trials of adding local wound infiltration with bupivacaine to usual anaesthetic care for THA and TKA.[57] The infiltration of local anaesthetic was found to be dominant compared to standard anaesthesia in both THA and TKA patients.

Three studies used Markov models to investigate similar measures to reduce surgical site infection in the United States,[46] United Kingdom,[47] and Australia.[48] The dominant strategy in all three included use of antibiotic-impregnated cement. The two studies that looked at other factors[47 48] each found use of prophylactic systemic antibiotics to be dominant over non-use, and use of conventional ventilation in operating theatres to be dominant over laminar airflow ventilation. Graves and colleagues[47] considered use of body exhaust suits, and found them to be dominated by strategies that did not include use of these suits. A fourth study investigated using single use negative pressure wound dressings to reduce surgical site infection.[49] This was found to be dominant compared with usual care.

For peer review only - http://bmjopen.bmj.com/site/about/guidelines.xhtml

Postoperative components

Fusco and Turchetti[50] used a Markov model to evaluate a strategy of ten face-to-face rehabilitation sessions followed by ten telerehabilitation sessions after TKA, compared to twenty face-to-face sessions. They found the strategy including telerehabilitation to be cost saving, and improved range of movement (knee flexion). However, they found no utility data for patients following a telerehabilitation programme so for their base case assumed it to be non-inferior to face-to-face rehabilitation. In a sensitivity analysis, if telerehabilitation conferred an improvement in quality of life of at least 2.5%, the strategy's probability of being cost-effective was 1; if it led to a reduction in quality of life of at least 2.5%, the probability of being cost-effective was 0 (each at a willingness to pay threshold of 30 000 euros per QALY).

Kauppila and colleagues[51] performed an economic evaluation of a ten-day outpatient rehabilitation course between two and four months after TKA that included clinical assessments, physical activity, sessions with a psychologist, and lectures from an orthopaedic surgeon, nutritionist and social worker.[55] They found that patients who completed this course had higher costs and slightly worse quality of life outcomes over the 1 year of follow-up (though the difference was non-significant at the 5% level) compared to those receiving conventional orthopaedic care.

Smith and colleagues[52] used a Markov model to investigate a post-operative strategy combining telephonic health coaching and financial incentives to increase physical activity. The intervention increased costs by 300 US dollars and was associated with an increase in utility of 0.005 QALYs. The point-estimate ICER was 57 200 dollars per QALY, with a 70% probability of being cost effective at a willingness to pay threshold of 100 000 US dollars per QALY.

Bolz and colleagues[53] compared three follow-up strategies: two-yearly routine follow-up; follow-up twice (at three months, and between one and two years after surgery); or no follow-up. The model assumed no revisions would be delayed in either strategy that included follow-up, and the outcomes for these two strategies were identical in each analysis. The no follow-up strategy was dominant for any assumed rate of delayed revision between 1% and 50%.

For peer review only - http://bmjopen.bmj.com/site/about/guidelines.xhtml

Effects of standardizing currencies and price years

Most studies showed one strategy to be dominant (i.e. cheaper and more effective) over the others[37 38 40 41 43 45-51 53] and were therefore not affected by changes in currency or price year. When converted into 2016 US PPP, willingness to pay thresholds ranged between 22 112 (Denmark) and 100 000 dollars (US; Supplementary Table A9). Autologous blood transfusion[44] and bariatric surgery[39] would be cost-effective across all willingness to pay thresholds identified in the review. The capture and replacement of red cells[42] cost 7.8 million US PPP dollars per QALY gained and would not be cost-effective by any study's thresholds. Telephonic health coaching and financial incentives to increase physical activity[52] were cost-effective at a willingness to pay of 100 000 dollars per QALY but not with any lower threshold. Accelerated care pathway in TKA patients in Denmark[38] would not be cost-effective in a US setting using a willingness to pay of 100 000 dollars per QALY.

Discussion

Summary of evidence

Our objective was to assess the cost-effectiveness evidence of enhanced recovery following THA or TKA through a systematic collection of published cost–utility data. Previous systematic reviews considered effectiveness[16] and patient satisfaction,[62] but we believe this is the first systematic review assessing the cost-effectiveness of enhanced recovery for THA or TKA patients. We identified two cost–utility analyses of an entire pathway. This is consistent with reviews of cost-effectiveness of enhanced recovery programmes for other surgical sites,[63-65] which have found few studies reported the effect on quality of life and none presented cost-effectiveness results using QALYs. Both studies that considered cost-effectiveness of an entire recovery pathway were trials in both TKA and THA patients.[37 38] The enhanced recovery pathway was found to be associated with reduced costs for all patients and the incremental cost-effectiveness estimate favoured the

For peer review only - http://bmjopen.bmj.com/site/about/guidelines.xhtml

enhanced recovery protocol, with a high probability of being the most cost-effective option.

We identified 15 studies presenting cost–utility data for components of an enhanced recovery pathway. These studies covered only a few of the potential enhanced recovery pathway components and were conducted across different healthcare systems using used different cost perspectives. Three studies investigated overlapping strategies for reducing surgical infections supporting the use of prophylactic systemic antibiotics, antibiotic-impregnated cement, and conventional ventilation.[46-48] No other interventions were examined by more than one study. Scope to combine or generalize results is therefore limited.

The studies identified in this review were generally of good quality according to the CHEC list[30] with a short time horizon identified as a key limitation in nine studies (between 6 weeks and 7 years). This is of significant concern as short time horizons will not capture or model the impact of the interventions on costs and benefits accruing over the long post-acute care period of interest. Furthermore, we also found that the large majority of studies did not consider ethical aspects and distributional implications of their findings. When the models were assessed against the ISPOR questionnaire,[31] there were concerns about the lack of model validation work, potentially questioning the reliability of 10 of the 12 studies identified. The trials were generally of good quality. One trial was a non-randomized before and after trial,[37] and participants were not blinded to the intervention for postoperative interventions in any of the studies. However, we concluded that the overall risk of bias among the studies appeared low.

Limitations of this review

We may have missed relevant evidence by limiting our search to reports published in the English language and excluding studies that did not report QALYs. Cost–utility analyses enable comparison between different interventions and are the preferred intelligence for healthcare allocation decisions.[18 19] We therefore felt justified in limiting our review to studies reporting QALYs. We identified one study excluded due to not reporting QALYs that investigated a complete enhanced

For peer review only - http://bmjopen.bmj.com/site/about/guidelines.xhtml

recovery pathway.[66] In this study, the recovery pathway was found to be cost-saving and associated with statistically significant differences in knee flexion and extension at six months. However, the clinical significance of these differences was not discussed and no health-related quality of life data were collected.[66]

A second limitation is that standard practices have changed during the period covered by the included studies. For example, many enhanced recovery techniques are now standard practice,[8] and Nherera and colleagues[49] even included enhanced recovery pathway as usual care in their model. Furthermore, other practices in the included studies are now outdated. For example, patients in the control group of Brunenberg and colleagues' trial had had an in-patient stay of 9.4 days,[37] whereas by 2015, the mean length of stay in England was 4.9 days for TKA and 5.4 days for THA[8] and has further decreased since.[67] Also, practices such as blood transfusion rates have changed greatly: the two studies included in this review assumed transfusion rates of 89%[44] to 100%,[42] whereas rates in 2014 were 22% for THA and 18% for TKA patients,[68] and rates of infection from blood transfusion are now lower than those used in the models.[69]

Research gaps for future work

Use of prophylactic systemic antibiotics and antibiotic-impregnated cement, and thromboprophylaxis[23-25] are the only measures investigated in more than one cost–utility analysis. There is therefore very wide scope for further investigation of components of enhanced recovery. Pathways have a 'whole package' benefit in excess of the sum of their parts and can be used to ensure dissemination of evidence-based practice and delivery of more consistent care.[70-72] From this perspective, specific components within a pathway might be less important than the degree of compliance with a protocol. However, as the use of care pathways becomes more prevalent, optimizing their components will be the next step in improving outcomes.

For peer review only - http://bmjopen.bmj.com/site/about/guidelines.xhtml

Conclusions

There is limited cost–utility evidence, either for an entire enhanced recovery pathway or for individual components of a pathway, for patients having THA or TKA. There are also concerns regarding the ability of short time horizons in trials in this area to capture relevant outcomes, and regarding a general lack of reporting of model validation. Our findings support the use of enhanced recovery pathways as a whole, prophylactic systemic antibiotics, antibiotic-impregnated cement and conventional ventilation. No other interventions were assessed by more than one study. Those single studies supported use of empirical preoperative *S. aureus* decolonization of all patients with mupiricin, single dose intravenous tranexamic acid, wound infiltration with local anaesthetic, single-use negative pressure wound therapy dressings, bariatric surgery for morbidly obese patients requiring TKA, use of telerehabilitation, and telephonic health coaching with financial incentives to increase physical activity. However, we were unable to conclude how transferable these findings would be into other healthcare systems. There is ample scope for future cost-effectiveness studies into enhanced recovery for THA and TKA patients. In particular, we recommend analysis of entire enhanced recovery pathways and comparison of incremental changes of components within pathways rather than considering aspects of care in isolation.

ACKNOWLEDGEMENTS

We are grateful to Nia Roberts, information specialist, and Rafael Pinedo-Villanueva, senior research associate in health economics, both at the University of Oxford, for their assistance with the search strategy.

AUTHOR STATEMENT:

JM, AJ and JL designed the study and all authors defined the inclusion criteria. JM, LYC and JL developed the search strategies. JM and LYC piloted the search strategy and inclusion criteria. JM and JL developed the data extraction pro forma. JM, LYC and JL screened the studies. JM, MGP and

For peer review only - http://bmjopen.bmj.com/site/about/guidelines.xhtml

RJ extracted the data. MGP wrote the draft of the manuscript. JM and JL provided critical revisions to the article and all authors approved the final version of the article to be published. JL is the guarantor of the review.

DATA STATEMENT:

All data relevant to the study are included in the article or uploaded as supplementary information.

PATIENT CONSENT:

Not required.

PROVENANCE AND PEER REVIEW

Not commissioned; externally peer reviewed.

For peer review only

FIGURE LEGENDS:

Figure 1. Flow diagram of studies included in this review and reasons for exclusion, modified from Moher, *et al*.[28] (NHS EED, National Health Service Economic Evaluations Database; QALY, quality-adjusted life year)

Figure 2. Assessments of study quality based on tools from (*a*) Consensus on Health Economic Criteria,[30] (*b*) International Society for Pharmacoeconomics and Outcomes Research,[31] (*c*) Assessment of the Validation Status of Health-Economic decision models tool,[32] (*d*) Cochrane Collaboration.[33] Note that the study by Brunenberg and colleagues[37] was a non-randomised before and after trial and we have included it in Figure 2*d* for completeness.

For peer review only - http://bmjopen.bmj.com/site/about/guidelines.xhtml

References

1. Pabinger C, Geissler A. Utilization rates of hip arthroplasty in OECD countries. *Osteoarthritis and cartilage* 2014;22(6):734-41. doi: 10.1016/j.joca.2014.04.009

2. Pabinger C, Lothaller H, Geissler A. Utilization rates of knee-arthroplasty in OECD countries. *Osteoarthritis and Cartilage* 2015;23(10):1664-73. doi: 10.1016/j.joca.2015.05.008

3. Ackerman IN, Bohensky MA, de Steiger R, et al. Lifetime risk of primary total hip replacement surgery for osteoarthritis from 2003 to 2013: A multinational analysis using national registry data. *Arthritis Care & Research* 2017;69(11):1659-67. doi: 10.1002/acr.23197

4. Ackerman IN, Bohensky MA, de Steiger R, et al. Substantial rise in the lifetime risk of primary total knee replacement surgery for osteoarthritis from 2003 to 2013: an international, population-level analysis. *Osteoarthritis Cartilage* 2017;25(4):455-61. doi: 10.1016/j.joca.2016.11.005 [published Online First: 2016/11/20]

5. Culliford DJ, Maskell J, Kiran A, et al. The lifetime risk of total hip and knee arthroplasty: results from the UK general practice research database. *Osteoarthritis Cartilage* 2012;20(6):519-24. doi: 10.1016/j.joca.2012.02.636 [published Online First: 2012/03/08]

6. Culliford D, Maskell J, Judge A, et al. Future projections of total hip and knee arthroplasty in the UK: results from the UK Clinical Practice Research Datalink. *Osteoarthritis Cartilage* 2015;23(4):594-600. doi: 10.1016/j.joca.2014.12.022 [published Online First: 2015/01/13]

7. Kehlet H. Multimodal approach to control postoperative pathophysiology and rehabilitation. *British journal of anaesthesia* 1997;78(5):606-17. doi: 10.1093/bja/78.5.606

8. Nagra NS, Hamilton TW, Strickland L, et al. Enhanced recovery programmes for lower limb arthroplasty in the UK. *Annals of the Royal College of Surgeons of England* 2017:1-6. doi: 10.1308/rcsann.2017.0124 [published Online First: 2017/08/05]

9. Auyong DB, Allen CJ, Pahang JA, et al. Reduced length of hospitalization in primary total knee arthroplasty patients using an updated enhanced recovery after orthopedic surgery (ERAS) pathway. *The Journal of arthroplasty* 2015;30(10):1705-09. doi: 10.1016/j.arth.2015.05.007

10. Maempel J, Walmsley P. Enhanced recovery programmes can reduce length of stay after total knee replacement without sacrificing functional outcome at one year. *The Annals of The Royal College of Surgeons of England* 2015;97(8):563-67. doi: 10.1308/rcsann.2015.0016

11. Dwyer AJ, Thomas W, Humphry S, et al. Enhanced recovery programme for total knee replacement to reduce the length of hospital stay. *Journal of Orthopaedic Surgery* 2014;22(2):150-54. doi: 10.1177/230949901402200206

12. Stowers MD, Lemanu DP, Coleman B, et al. Review article: Perioperative care in enhanced recovery for total hip and knee arthroplasty. *Journal of Orthopaedic Surgery* 2014;22(3):383-92. doi: 10.1177/230949901402200324

13. Ibrahim MS, Alazzawi S, Nizam I, et al. An evidence-based review of enhanced recovery interventions in knee replacement surgery. *Annals of the Royal College of Surgeons of England* 2013;95(6):386-9. doi: 10.1308/003588413x13629960046435 [published Online First: 2013/09/13]

14. McDonald D, Siegmeth R, Deakin A, et al. An enhanced recovery programme for primary total knee arthroplasty in the United Kingdom—follow up at one year. *The Knee* 2012;19(5):525-29. doi: 10.1016/j.knee.2011.07.012

15. Malviya A, Martin K, Harper I, et al. Enhanced recovery program for hip and knee replacement reduces death rate: A study of 4,500 consecutive primary hip and knee replacements. *Acta Orthopaedica* 2011;82(5):577-81. doi: 10.3109/17453674.2011.618911

16. Zhu S, Qian W, Jiang C, et al. Enhanced recovery after surgery for hip and knee arthroplasty: a systematic review and meta-analysis. *Postgraduate Medical Journal* 2017;93(1106):736-42. doi: 10.1136/postgradmedj-2017-134991

17. Stowers MDJ, Manuopangai L, Hill AG, et al. Enhanced Recovery After Surgery in elective hip and knee arthroplasty reduces length of hospital stay. *ANZ Journal of Surgery* 2016;86(6):475-79.

For peer review only - http://bmjopen.bmj.com/site/about/guidelines.xhtml

doi: 10.1111/ans.13538
18. National Institute for Health and Care Excellence. Guide to the methods of technology appraisal 2013 2013 [Available from: https://www.nice.org.uk/process/pmg9/chapter/foreword accessed September 6 2017.
19. Weinstein MC, Siegel JE, Gold MR, et al. Recommendations of the panel on cost-effectiveness in health and medicine. *JAMA* 1996;276(15):1253-58. doi: 10.1001/jama.1996.03540150055031
20. Schwarzer R, Rochau U, Saverno K, et al. Systematic overview of cost-effectiveness thresholds in ten countries across four continents. *Journal of comparative effectiveness research* 2015;4(5):485-504. doi: 10.2217/cer.15.38 [published Online First: 2015/10/23]
21. Nwachukwu BU, Bozic KJ, Schairer WW, et al. Current status of cost utility analyses in total joint arthroplasty: a systematic review. *Clinical orthopaedics and related research* 2015;473(5):1815-27. doi: 10.1007/s11999-014-3964-4 [published Online First: 2014/10/01]
22. Daigle ME, Weinstein AM, Katz JN, et al. The cost-effectiveness of total joint arthroplasty: a systematic review of published literature. *Best practice & research Clinical rheumatology* 2012;26(5):649-58. doi: 10.1016/j.berh.2012.07.013 [published Online First: 2012/12/12]
23. Brockbank J, Wolowacz S. Economic evaluations of new oral anticoagulants for the prevention of venous thromboembolism after total hip or knee replacement: A systematic review. *Pharmacoeconomics* 2017;35:517-35. doi: 10.1007/s40273-017-0486-4
24. Kapoor A, Chuang W, Radhakrishnan N, et al. Cost effectiveness of venous thromboembolism pharmacological prophylaxis in total hip and knee replacement: A systematic review. *Pharmacoeconomics* 2010;28(7):521-38. doi: 10.2165/11535210-000000000-00000
25. Wolowacz S, Hess N, Brennan V, et al. Cost-effectiveness of venous thromboembolism prophylaxis in total hip and knee replacement surgery: the evolving application of health economic modelling over 20 years. *Current medical research and opinion* 2008;24(10):2993-3006. doi: 10.1185/03007990802443255
26. Murphy J, Pritchard MG, Cheng LY, et al. Cost-effectiveness of enhanced recovery in hip and knee replacement: a systematic review protocol. *BMJ Open* 2018;8(3):e019740. doi: https://dx.doi.org/10.1136/bmjopen-2017-019740
27. Murphy J, Cheng, LY., Leal, J. Cost-effectiveness and quality of life of enhanced recovery in hip and knee replacement: a systematic review protocol. PROSPERO 2017:CRD42017059473 Available from http://www.crd.york.ac.uk/PROSPERO/display_record.asp?ID=CRD42017059473. PROSPERO International prospective register of systematic reviews, 2017.
28. Moher D, Liberati A, Tetzlaff J, et al. Preferred reporting items for systematic reviews and meta-analyses: the PRISMA statement. *PLoS medicine* 2009;6(7):e1000097. doi: 10.1371/journal.pmed.1000097
29. National Joint Registry. 13th annual report 2016: National Joint Registry for England, Wales, Northern Ireland and the Isle of Man surgical data to 31 December 2015. Hemel Hempstead, UK: National Joint Registry, 2016.
30. Evers S, Goossens M, de Vet H, et al. Criteria list for assessment of methodological quality of economic evaluations: Consensus on Health Economic Criteria. *International journal of technology assessment in health care* 2005;21(2):240-5. [published Online First: 2005/06/01]
31. Caro JJ, Eddy DM, Kan H, et al. Questionnaire to assess relevance and credibility of modeling studies for informing health care decision making: an ISPOR-AMCP-NPC Good Practice Task Force report. *Value in health* 2014;17(2):174-82. doi: 10.1016/j.jval.2014.01.003.
32. Vemer P, Corro Ramos I, van Voorn GA, et al. AdViSHE: A validation-assessment tool of health-economic models for decision makers and model users. *Pharmacoeconomics* 2016;34(4):349-61. doi: 10.1007/s40273-015-0327-2 [published Online First: 2015/12/15]
33. Higgins JP, Altman DG, Gøtzsche PC, et al. The Cochrane Collaboration's tool for assessing risk of bias in randomised trials. *BMJ (Clinical research ed)* 2011;343:d5928. doi:

For peer review only - http://bmjopen.bmj.com/site/about/guidelines.xhtml

10.1136/bmj.d5928 [published Online First: 2011/10/20]
34. Cooper N, Coyle D, Abrams K, et al. Use of evidence in decision models: an appraisal of health technology assessments in the UK since 1997. *Journal of Health Services Research & Policy* 2005;10(4):245-50. doi: 10.1258/135581905774414187
35. Coyle D, Lee KM. Evidence-based economic evaluation: how the use of different data sources can impact results. In: Donaldson C, Mugford M, Vale L, eds. Evidence-based health economics: from effectiveness to efficiency in systematic review. London, UK: BMJ Publishing Group 2002:55-66.
36. Organisation for Economic Co-operation and Development. Prices and purchasing power parities 2017 [Available from: http://stats.oecd.org/Index.aspx?DataSetCode=CPL accessed September 19 2017.
37. Brunenberg DE, van Steyn MJ, Sluimer JC, et al. Joint recovery programme versus usual care: an economic evaluation of a clinical pathway for joint replacement surgery. *Medical care* 2005;43(10):1018-26. [published Online First: 2005/09/17]
38. Larsen K, Hansen TB, Thomsen PB, et al. Cost-effectiveness of accelerated perioperative care and rehabilitation after total hip and knee arthroplasty. *Journal of Bone and Joint Surgery American volume* 2009;91(4):761-72. doi: 10.2106/JBJS.G.01472
39. McLawhorn AS, Southren D, Wang YC, et al. Cost-effectiveness of bariatric surgery prior to total knee arthroplasty in the morbidly obese: A computer model-based evaluation. *Journal of Bone & Joint Surgery - American Volume* 2016;98(2):e6. doi: 10.2106/JBJS.N.00416
40. Fernandes L, Roos EM, Overgaard S, et al. Supervised neuromuscular exercise prior to hip and knee replacement: 12-month clinical effect and cost-utility analysis alongside a randomised controlled trial. *BMC Musculoskelet Disord* 2017;18(1):5. doi: https://dx.doi.org/10.1186/s12891-016-1369-0
41. Courville XF, Tomek IM, Kirkland KB, et al. Cost-effectiveness of preoperative nasal mupirocin treatment in preventing surgical site infection in patients undergoing total hip and knee arthroplasty: a cost-effectiveness analysis. *Infection Control and Hospital Epidemiology* 2012;33(2):152-59. doi: 10.1086/663704
42. Jackson BR, Umlas J, AuBuchon JP. The cost-effectiveness of postoperative recovery of RBCs in preventing transfusion-associated virus transmission after joint arthroplasty. *Transfusion* 2000;40(9):1063-6.
43. Ramkumar DB, Ramkumar N, Tapp SJ, et al. Pharmacologic Hemostatic Agents in Total Joint Arthroplasty-A Cost-Effectiveness Analysis. *J Arthroplasty* 2018;33(7):2092-99.e9. doi: https://dx.doi.org/10.1016/j.arth.2018.02.068
44. Sonnenberg FA. A health economic analysis of autologous transfusion. *Infusionstherapie und Transfusionsmedizin* 2002;29(3):175-83.
45. Marques EMR, Blom AW, Lenguerrand E, et al. Local anaesthetic wound infiltration in addition to standard anaesthetic regimen in total hip and knee replacement: Long-term cost-effectiveness analyses alongside the APEX randomised controlled trials. *BMC Medicine* 2015;13:151. doi: 10.1186/s12916-015-0389-1
46. Cummins JS, Tomek IM, Kantor SR, et al. Cost-effectiveness of antibiotic-impregnated bone cement used in primary total hip arthroplasty. *Journal of Bone and Joint Surgery American volume* 2009;91(3):634-41. doi: 10.2106/JBJS.G.01029
47. Graves N, Wloch C, Wilson J, et al. A cost-effectiveness modelling study of strategies to reduce risk of infection following primary hip replacement based on a systematic review. *Health Technology Assessment (Winchester, England)* 2016;20(54):1-144. doi: 10.3310/hta20540
48. Merollini KM, Crawford RW, Whitehouse SL, et al. Surgical site infection prevention following total hip arthroplasty in Australia: a cost-effectiveness analysis. *American Journal of Infection Control* 2013;41(9):803-9. doi: 10.1016/j.ajic.2012.11.015
49. Nherera LM, Trueman P, Karlakki SL. Cost-effectiveness analysis of single-use negative pressure wound therapy dressings (sNPWT) to reduce surgical site complications (SSC) in routine

For peer review only - http://bmjopen.bmj.com/site/about/guidelines.xhtml

primary hip and knee replacements. *Wound Repair Regen* 2017;25(3):474-82. doi: https://dx.doi.org/10.1111/wrr.12530
50. Fusco F, Turchetti G. Telerehabilitation after total knee replacement in Italy: cost-effectiveness and cost-utility analysis of a mixed telerehabilitation-standard rehabilitation programme compared with usual care. *BMJ Open* 2016;6(5):e009964. doi: 10.1136/bmjopen-2015-009964
51. Kauppila AM, Sintonen H, Aronen P, et al. Economic evaluation of multidisciplinary rehabilitation after primary total knee arthroplasty based on a randomized controlled trial. *Arthritis care & research* 2011;63(3):335-41. doi: 10.1002/acr.20398
52. Smith KC, Paltiel AD, Yang HY, et al. Cost-effectiveness of health coaching and financial incentives to promote physical activity after total knee replacement. *Osteoarthritis Cartilage* 2018;26(11):1495-505. doi: https://dx.doi.org/10.1016/j.joca.2018.07.014
53. Bolz KMD, Crawford RW, Donnelly B, et al. The cost-effectiveness of routine follow-up after primary total hip arthroplasty. *J Arthroplasty* 2010;25(2):191-96. doi: 10.1016/j.arth.2008.12.009
54. Larsen K, Sørensen OG, Hansen TB, et al. Accelerated perioperative care and rehabilitation intervention for hip and knee replacement is effective: a randomized clinical trial involving 87 patients with 3 months of follow-up. *Acta Orthop* 2008;79(2):149-59. doi: 10.1080/17453670710014923 [published Online First: 2008/05/15]
55. Kauppila A-M, Kyllönen E, Ohtonen P, et al. Multidisciplinary rehabilitation after primary total knee arthroplasty: a randomized controlled study of its effects on functional capacity and quality of life. *Clinical rehabilitation* 2010;24(5):398-411. doi: 10.1177/0269215509346089
56. Villadsen A, Overgaard S, Holsgaard-Larsen A, et al. Postoperative effects of neuromuscular exercise prior to hip or knee arthroplasty: a randomised controlled trial. *Annals of the rheumatic diseases* 2014;73(6):1130-7. doi: 10.1136/annrheumdis-2012-203135 [published Online First: 2013/05/11]
57. Wylde V, Lenguerrand E, Gooberman-Hill R, et al. Effect of local anaesthetic infiltration on chronic postsurgical pain after total hip and knee replacement: the APEX randomised controlled trials. *Pain* 2015;156(6):1161-70. doi: 10.1097/j.pain.0000000000000114
58. Briggs A, Sculpher M, Britton A, et al. The costs and benefits of primary total hip replacement. How likely are new prostheses to be cost-effective? *International journal of technology assessment in health care* 1998;14(4):743-61. [published Online First: 1999/01/14]
59. Birkmeyer JD, Goodnough LT, AuBuchon JP, et al. The cost-effectiveness of preoperative autologous blood donation for total hip and knee replacement. *Transfusion* 1993;33(7):544-51.
60. Sonnenberg FA, Gregory P, Yomtovian R, et al. The cost-effectiveness of autologous transfusion revisited: implications of an increased risk of bacterial infection with allogeneic transfusion. *Transfusion* 1999;39(8):808-17.
61. Losina E, Walensky RP, Kessler CL, et al. Cost-effectiveness of total knee arthroplasty in the United States: patient risk and hospital volume. *Archives of internal medicine* 2009;169(12):1113-21; discussion 21-2. doi: 10.1001/archinternmed.2009.136 [published Online First: 2009/06/24]
62. Jones E, Wainwright T, Foster J, et al. A systematic review of patient reported outcomes and patient experience in enhanced recovery after orthopaedic surgery. *The Annals of The Royal College of Surgeons of England* 2014;96(2):89-94. doi: 10.1308/003588414x13824511649571
63. Stowers MD, Lemanu DP, Hill AG. Health economics in Enhanced Recovery After Surgery programs. *Canadian journal of anaesthesia = Journal canadien d'anesthesie* 2015;62(2):219-30. doi: 10.1007/s12630-014-0272-0 [published Online First: 2014/11/14]
64. Lemanu DP, Singh PP, Stowers MD, et al. A systematic review to assess cost effectiveness of enhanced recovery after surgery programmes in colorectal surgery. *Colorectal disease : the*

For peer review only - http://bmjopen.bmj.com/site/about/guidelines.xhtml

official journal of the Association of Coloproctology of Great Britain and Ireland 2014;16(5):338-46. doi: 10.1111/codi.12505 [published Online First: 2013/11/29]
65. Lee L, Li C, Landry T, et al. A systematic review of economic evaluations of enhanced recovery pathways for colorectal surgery. *Annals of surgery* 2014;259(4):670-6. doi: 10.1097/SLA.0b013e318295fef8 [published Online First: 2013/05/16]
66. Köksal I, Tahta M, Şimşek ME, et al. Efficacy of rapid recovery protocol for total knee arthroplasty: a retrospective study. *Acta Orthopaedica et Traumatologica Turcica* 2015;49(4):382-6. doi: 10.3944/AOTT.2015.14.0353
67. Garriga C, Murphy J, Leal J, et al. Impact of a national enhanced recovery after surgery programme on patient outcomes of primary total knee replacement: an interrupted time series analysis from "The National Joint Registry of England, Wales, Northern Ireland and the Isle of Man". *Osteoarthritis Cartilage* 2019;27(9):1280-93. doi: 10.1016/j.joca.2019.05.001 [published Online First: 2019/05/13]
68. Hart A, Khalil JA, Carli A, et al. Blood transfusion in primary total hip and knee arthroplasty. Incidence, risk factors, and thirty-day complication rates. *J Bone Joint Surg Am* 2014;96(23):1945-51. doi: 10.2106/JBJS.N.00077
69. Norfolk D, editor. *Handbook of transfusion medicine*. Norwich, UK: TSO, 2013.
70. Simpson JC, Moonesinghe SR, Grocott MPW, et al. Enhanced recovery from surgery in the UK: an audit of the enhanced recovery partnership programme 2009–2012. *BJA: British Journal of Anaesthesia* 2015;115(4):560-68. doi: 10.1093/bja/aev105
71. Panella M, Marchisio S, Di Stanislao F. Reducing clinical variations with clinical pathways: do pathways work? *International journal for quality in health care : journal of the International Society for Quality in Health Care* 2003;15(6):509-21. doi: 10.1093/intqhc/mzg057 [published Online First: 2003/12/09]
72. Rotter T, Kinsman L, James E, et al. Clinical pathways: effects on professional practice, patient outcomes, length of stay and hospital costs. *The Cochrane database of systematic reviews* 2010(3):CD006632. doi: 10.1002/14651858.CD006632.pub2 [published Online First: 2010/03/20]

For peer review only

For peer review only - http://bmjopen.bmj.com/site/about/guidelines.xhtml

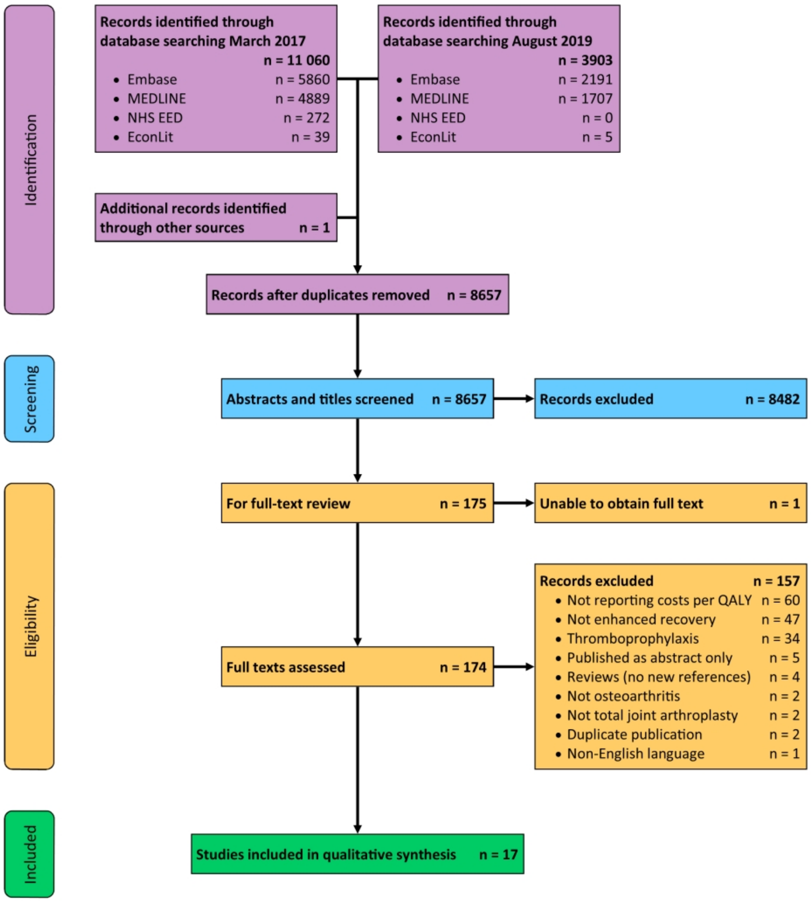

Figure 1. Flow diagram of studies included in this review and reasons for exclusion, modified from Moher, et al.28 (NHS EED, National Health Service Economic Evaluations Database; QALY, quality-adjusted life year)

209x297mm (300 x 300 DPI)

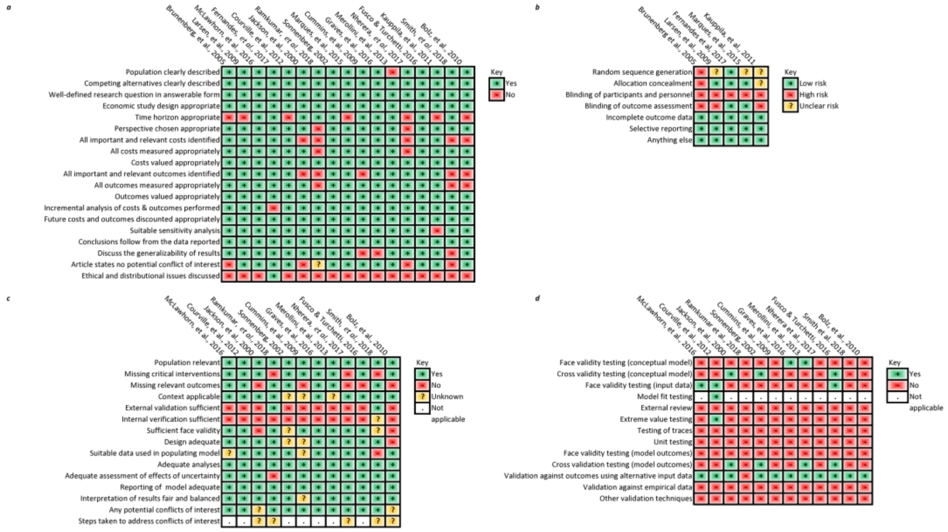

Figure 2. Assessments of study quality based on tools from (a) Consensus on Health Economic Criteria,30 (b) International Society for Pharmacoeconomics and Outcomes Research,31 (c) Assessment of the Validation Status of Health-Economic decision models tool,32 (d) Cochrane Collaboration.33 Note that the study by Brunenberg and colleagues37 was a non-randomised before and after trial and we have included it in Figure 2d for completeness.

297x209mm (300 x 300 DPI)

Supplemental Material: Enhanced recovery following hip and knee arthroplasty

Table A1. Full search terms for databases included in this review

Query	Search term
Ovid MEDLINE	
1	arthroplasty, replacement, hip/ or arthroplasty, replacement, knee/
2	((knee? or hip) adj (replace$ or arthroplast*)).ti,ab.
3	1 or 2
4	simulation model$.ti,ab.
5	markov.ti,ab.
6	monte carlo.ti,ab.
7	decision tree$.ti,ab.
8	decision analy$.ti,ab.
9	qaly$.ti,ab.
10	(valu$ adj2 quality).ti,ab.
11	utility value$.ti,ab.
12	((disability or quality) adj adjusted).ti,ab.
13	((life adj2 year$) or health year equivalent$).ti,ab.
14	(health adj utilit$).ti,ab.
15	hui$1.ti,ab.
16	(quality adj3 well$).ti,ab.
17	qwb.ti,ab.
18	(qald$ or qale$ or qtime$).ti,ab.
19	(well being or wellbeing).tw.
20	(health adj2 stat$).tw.
21	((adjusted adj2 life) or qaly$).ti,ab.
22	(daly or qol or hql or hqol or hrqol or hr ql or hrql).tw.
23	cost-utility.ti,ab.
24	cost-effectiveness.ti,ab.
25	cost-benefit.ti,ab.
26	cost-minimisation.ti,ab.
27	cost-minimization.ti,ab.
28	modelling.ti,ab.
29	modeling.ti,ab.
30	decision model.ti,ab.
31	QALY.ti,ab.
32	quality adjusted life year$.ti,ab.
33	cost.ti,ab.
34	life year$.ti,ab.
35	incremental cost-effectiveness ratio.ti,ab.
36	(quality adj2 life).ti,ab.
37	Technology Assessment, Biomedical/
38	"Costs and Cost Analysis"/
39	technology assessment$.ti,ab.
40	economic evaluation$.ti,ab.
41	economic model$.ti,ab.
42	discrete event simulat$.ti,ab.
43	cost utility.ti,ab.
44	cost effectiv$.ti,ab.
45	cost benefit.ti,ab.
46	cost minimisation.ti,ab.
47	cost minimization.ti,ab.
48	ICER$.ti,ab.
49	EQ-5D$.ti,ab.
50	(SF-12 or SF12 or Short Form 12).ti,ab.
51	(SF-36 or SF36 or Short Form 36).ti,ab.
52	(SF-6D or SF6D or Short Form 6D).ti,ab.

For peer review only

For peer review only - http://bmjopen.bmj.com/site/about/guidelines.xhtml

Supplemental Material: Enhanced recovery following hip and knee arthroplasty

53	rosser index.ti,ab.
54	person trade off.ti,ab.
55	standard gamble.ti,ab,kw.
56	time trade off.ti,ab,kw.
57	Hye.ti,ab,kw.
58	Hyes.ti,ab,kw.
59	Euroquol.ti,ab,kw.
60	4 or 5 or 6 or 7 or 8 or 9 or 10 or 11 or 12 or 13 or 14 or 15 or 16 or 17 or 18 or 19 or 20 or 21 or 22 or 23 or 24 or 25 or 26 or 27 or 28 or 29 or 30 or 31 or 32 or 33 or 34 or 35 or 36 or 37 or 38 or 39 or 40 or 41 or 42 or 43 or 44 or 45 or 46 or 47 or 48 or 49 or 50 or 51 or 52 or 53 or 54 or 55 or 56 or 57 or 58 or 59
61	3 and 60
EMBASE	
1	hip replacement/ or hip arthroplasty/
2	total knee replacement/ or knee replacement/ or knee arthroplasty/
3	((knee? or hip) adj (replace$ or arthroplast$)).ti,ab.
4	1 or 2 or 3
5	simulation model$.ti,ab.
6	markov.ti,ab.
7	monte carlo.ti,ab.
8	decision tree$.ti,ab.
9	decision analy$.ti,ab.
10	qaly$.ti,ab.
11	(valu$ adj2 quality).ti,ab.
12	utility value$.ti,ab.
13	((disability or quality) adj adjusted).ti,ab.
14	((life adj2 year$) or health year equivalent$).ti,ab.
15	hui$1.ti,ab.
16	(quality adj3 well$).ti,ab.
17	qwb.ti,ab.
18	(qald$ or qale$ or qtime$).ti,ab.
19	(well being or wellbeing).tw.
20	(health adj2 stat$).tw.
21	((adjusted adj2 life) or qaly$).ti,ab.
22	(daly or qol or hql or hqol or hrqol or hr ql or hrql).tw.
23	cost-utility.ti,ab.
24	cost-benefit.ti,ab.
25	cost-minimisation.ti,ab.
26	cost-minimization.ti,ab.
27	modelling.ti,ab.
28	modeling.ti,ab.
29	QALY.ti,ab.
30	quality adjusted life year$.ti,ab.
31	cost.ti,ab.
32	life year$.ti,ab.
33	incremental cost-effectiveness ratio.ti,ab.
34	(quality adj2 life).ti,ab.
35	decision model$.ti,ab.
36	cost-effectiv$.ti,ab.
37	"cost benefit analysis"/
38	biomedical technology assessment/
39	technology assessment$.ti,ab.
40	economic evaluation$.ti,ab.
41	economic model$.ti,ab.
42	discrete event simulat$.ti,ab.
43	cost utility.ti,ab.
44	cost effectiv$.ti,ab.

For peer review only

For peer review only - http://bmjopen.bmj.com/site/about/guidelines.xhtml

Supplemental Material: Enhanced recovery following hip and knee arthroplasty

45	cost benefit.ti,ab.
46	cost minimisation.ti,ab.
47	cost minimization.ti,ab.
48	ICER$.ti,ab.
49	(health adj utilit$).ti,ab.
50	EQ-5D$.ti,ab.
51	(SF-12 or SF12 or Short Form 12).ti,ab.
52	(SF-36 or SF36 or Short Form 36).ti,ab.
53	(SF-6D or SF6D or Short Form 6D).ti,ab.
54	rosser index.ti,ab.
55	person trade off.ti,ab.
56	standard gamble.ti,ab,kw.
57	time trade off.ti,ab,kw.
58	Hye.ti,ab,kw.
59	Hyes.ti,ab,kw.
60	Euroquol.ti,ab,kw.
61	5 or 6 or 7 or 8 or 9 or 10 or 11 or 12 or 13 or 14 or 15 or 16 or 17 or 18 or 19 or 20 or 21 or 22 or 23 or 24 or 25 or 26 or 27 or 28 or 29 or 30 or 31 or 32 or 33 or 34 or 35 or 36 or 37 or 38 or 39 or 40 or 41 or 42 or 43 or 44 or 45 or 46 or 47 or 48 or 49 or 50 or 51 or 52 or 53 or 54 or 55 or 56 or 57 or 58 or 59 or 60
62	4 and 61
National Health Service Economic Evaluations Database, via the Cochrane Library	
Hip	Title, Abstract, Keywords: “Hip arthroplasty” OR<br>Title, Abstract, Keywords: “Hip arthroplasties” OR<br>Title, Abstract, Keywords: “Hip replacement”
Knee	Title, Abstract, Keywords: “Knee arthroplasty” OR<br>Title, Abstract, Keywords: “Knee arthroplasties” OR<br>Title, Abstract, Keywords: “Knee replacement”
EconLit	
	TI,AB(hip) OR TI,AB(knee) AND<br>TI,AB(Replace*) OR TI,AB(arthroplasty*) OR TI,AB(Replacement) OR TI,AB(arthroplasties)

For peer review only

For peer review only - http://bmjopen.bmj.com/site/about/guidelines.xhtml

Supplemental Material: Enhanced recovery following hip and knee arthroplasty

Table A2. Hierarchy used to classify data sources used in studies, modified from Cooper and colleagues[1] and Coyle and colleagues[2]

*Source of data for clinical effect sizes, adverse events & complications**	
1	Meta-analysis of RCTs with direct comparison between comparator therapies, measuring final outcomes
2	Single RCT with direct comparison between comparator therapies, measuring final outcomes
3	Meta-analysis of RCTs with direct comparison between comparator therapies, measuring surrogate outcomes; *or*<br>meta-analysis of placebo-controlled RCTs with similar trial populations, measuring final outcomes for each individual therapy
4	Single RCT with direct comparison between comparator therapies, measuring surrogate outcomes; *or* single placebo-controlled RCTs with similar trial populations, measuring final outcomes for each individual therapy
5	Meta-analysis of placebo-controlled RCTs with similar trial populations, measuring surrogate outcomes
6	Single placebo-controlled RCTs with similar trial populations, measuring surrogate outcomes for each individual therapy
7	Case–control or cohort studies
8	Non-analytic studies, for example, case reports, case series
9	Expert opinion
*Source of baseline clinical data**	
1	Case series or analysis of reliable administrative databases specifically conducted for the study covering patients solely from the jurisdiction of interest
2	Recent case series or analysis of reliable administrative databases covering patients solely from the jurisdiction of interest
3	Recent case series or analysis of reliable administrative databases covering patients solely from another jurisdiction
4	Old case series or analysis of reliable administrative databases; *or*<br>estimates from RCTs
5	Estimates from previously published economic analyses: unsourced
6	Expert opinion
*Source of data for duration of primary effect**	
1	Analysis of reliable administrative databases specifically conducted for the study covering patients solely from the jurisdiction of interest
2	Recent analysis of reliable administrative databases covering patients solely from the jurisdiction of interest
3	Recent analysis of reliable administrative databases covering patients solely from another jurisdiction
4	Old analysis of reliable administrative databases
5	Estimates from previously published economic analyses: unsourced
6	Expert opinion
*Source of data for resource use**	
1	Prospective data collection; *or*<br>analysis of reliable administrative data from same jurisdiction for specific study
2	Recently published results of prospective data collection from the same; *or*<br>recent analysis of reliable administrative data from the same jurisdiction
3	Unsourced data from previous economic evaluations from the same jurisdiction
4	Recently published results of prospective data collection from a different jurisdiction; *or*<br>recent analysis of reliable administrative data from a different jurisdiction
5	Unsourced data from previous economic evaluation from a different jurisdiction
6	Expert opinion
Source of data for costs	
1	Cost calculations based on reliable databases or data sources conducted for specific study—same jurisdiction
2	Recently published cost calculations based on reliable databases or data sources—same jurisdiction
3	Unsourced data from previous economic evaluation—same jurisdiction

For peer review only

For peer review only - http://bmjopen.bmj.com/site/about/guidelines.xhtml

Supplemental Material: Enhanced recovery following hip and knee arthroplasty

4	Recently published cost calculations based on reliable databases or data sources—different jurisdiction
5	Unsourced data from previous economic evaluation—different jurisdiction
6	Expert opinion
Source of data for utilities	
1	Direct utility assessment for the specific study from a sample either:<br>of the general population, or<br>with knowledge of the disease(s) of interest, or<br>of patients with the disease(s) of interest; *or*<br>indirect utility assessment for the specific study from patient sample with disease(s) of interest, using a tool validated for the patient population
2	Direct utility assessment from a previous study from a sample either:<br>of the general population, or<br>with knowledge of the disease(s) of interest, or<br>of patients with the disease(s) of interest; *or*<br>indirect utility assessment from a previous study from patient sample with disease(s) of interest, using a tool validated for the patient population
3	Indirect utility assessment from a patient sample with disease(s) of interest, using a tool not validated for the patient population; *or*<br>patient preference values obtained from a visual analogue scale
4	Delphi panels; *or*<br>expert opinion

* Hierarchy used for assessment of models only.
RCT, randomized controlled trial

For peer review only - http://bmjopen.bmj.com/site/about/guidelines.xhtml

Supplemental Material: Enhanced recovery following hip and knee arthroplasty

Table A3. Overview of studies included in the review.

Authors	Population	Perspective	Horizon	Discounting	Currency, year
**Enhanced recovery pathway**					
Brunenberg 2005[37]	Patients on the waiting list for either a THA (48 patients recruited to Joint Recovery Programme (JRP), 50 to usual care) or TKA (30 patients to JRP, 32 to usual care), mean age 64.4 years, one-third male	Societal	1 year	None	USD, 2002
Larsen 2009[38]	Patients in trial[50] having THA, THA or UKA, mean age having accelerated intervention 64, 26 women, 30 men	Societal	1 year	None	DKK, 2006
**Preoperative:** *Assessment and optimization of comorbidities*					
McLawhorn 2016[39]	50-year-old morbidly obese patient with end-stage unilateral knee osteoarthritis	Societal	40 years	3%	USD, 2012
**Preoperative:** *Exercise*					
Fernandes 2017[40]	Adults having unilateral THA or TKA for symptomatic osteoarthritis	Healthcare sector	61 weeks	None	EUR, 2012
**Preoperative:** *Staphylococcus aureus prophylaxis*					
Courville 2012[41]	65-year-olds with end stage hip or knee osteoarthritis for whom medical management has failed and TJA recommended.	Societal	1 year	None	USD, 2005
**Intraoperative:** *Avoid unnecessary blood transfusion*					
Jackson 2000[42]	Patients undergoing TJA, average age 65 years, range 20 to 80 years	Not stated	Lifetime	3% and 5%	USD, not reported
Ramkumar 2018[43]	65-year-old having TJA for severe osteoarthritits	Medical costs	10 years	3%	
Sonnenberg 2002[44]	65-year-old of composite sex and race undergoing THA	Not stated	Lifetime	3%	USD, 2000
**Intraoperative:** *Local infiltration of analgesia*					
Marques 2015[45]	Patients in trial[51] having THA or TKA, mean ages 66–69.5 years, 52%–65% female	Health and social care payer	1 year	None	GBP, not reported
**Intraoperative:** *Infection prevention*					
Cummins 2009[46]	68-year-olds of average health for their age undergoing THA due to degenerative arthritis	Hospital	Lifetime	3%	USD, 2002
Graves 2016[47]	Simulated cohort of patients who had THA in 2012	UK NHS	Lifetime	3%	GBP, 2012
Merollini 2013[48]	Not described	Healthcare system	30 years	3%	AUD, 2011
Nherera 2017[49]	Adults having routine TJA	UK NHS	6 weeks	None	GBP, 2015
**Postoperative:** *Physical therapy*					
Fusco & Turchetti, 2016[50]	Average age of 60 years, 44% males, 19% experienced complications during or after surgery	Societal & healthcare service	Lifetime	3%	Euro, 2013
Smith 2018[52]	Patients having unilateral TKA for knee osteoarthritis	Healthcare sector	Lifetime	3%	USD, 2016
Kauppila 2011[51]	60–80 year olds having unilateral TKR for knee osteoarthritis	Healthcare system	1 year	None	Euro, 2006

For peer review only - http://bmjopen.bmj.com/site/about/guidelines.xhtml

Supplemental Material: Enhanced recovery following hip and knee arthroplasty

**Postoperative:** *Scheduling of follow-up*					
Bolz 2010[53]	THA patients, mean age 69.9 years	Health services	7 years	3%	AUD, not reported

NHS: National Health Service; THA: total hip arthroplasty; TJA: total joint arthroplasty; TKA: total knee arthroplasty; UKA: unicompartmental knee arthroplasty; AUD: Australian dollar; DKK: Danish krone; GBP: British pound; USD: US dollar

For peer review only

For peer review only - http://bmjopen.bmj.com/site/about/guidelines.xhtml

Supplemental Material: Enhanced recovery following hip and knee arthroplasty

Table A4. Costs included in each study

	Direct medical	Direct treatment	In-patient	Out-patient	Day care	Community healthcare	Medication	Side effect costs	Staff	Labs/diagnostic	Capital equipment	Social care	Travel costs	Productivity losses	Income forgone due to illness
Brunenberg 2005[37]	+	+	+	+		+	+		+			+		+	+
Larsen 2009[38]	+	+	+	+		+	+		+					+	+
McLawhorn 2016[39]	+	+	+					+							
Fernandes 2017[40]	+	+							+				+		+
Courville 2012[41]	+	+	+				+			+					
Jackson 2000[42]	+	+	+	+			+		+	+					
Ramkumar 2018[43]	+	+					+								
Sonnenberg 2002[44]	+	+	+	+			+								
Marques 2015[45]	+	+	+	+	+	+	+		+			+			
Cummins 2009[46]	+	+	+												
Graves 2016[47]	+	+	+				+				+				
Merollini 2013[48]	+	+	+				+		+		+				
Nherera 2017[49]	+	+	+	+		+	+								
Fusco & Turchetti, 2016[50]	+	+	+	+					+		+		+	+	+
Kauppila 2011[51]	+	+	+	+	+	+						+			
Smith 2018[52]	+	+					+	+							
Bolz 2010[53]	+		+	+						+					

For peer review only

Supplemental Material: Enhanced recovery following hip and knee arthroplasty

Table A5. Populations and tools used for eliciting utilities.

Authors	EQ-5D	15 D	QWB	SG	VAS / rating scale	TTO	AQoL	Other
Studies eliciting utilities								
Brunenberg 2005[37]	Patients in trial (UK tariff)	—	—	—	—	—	—	—
Larsen 2009[38]	Patients in trial (Danish tariff)	—	—	—	—	—	—	—
Fernandes 2017[40]	Patients in trial (Danish tariff)	—	—	—	—	—	—	—
Marques 2015[45]	Patients in trial (UK tariff)	—	—	—	—	—	—	—
Fusco & Turchetti, 2016[50]	TKR patients in trial (UK tariff)	—	—	—	—	—	—	—
Smith 2018[52]	Patients in trial (tariff not reported)	—	—	—	—	—	—	—
Kauppila 2011[50]	—	Patients in trial (Finnish tariff)	—	—	—	—	—	—
Studies using published utilities[*]								
McLawhorn 2016[39]	TKR patients in a trial; patients having bariatric surgery	—	—	—	Patients having bariatric surgery	—	—	—
Courville 2012[41]	—	—	Population reporting arthritis	—	—	—	—	—
Jackson 2000[42]	—	—	People with HIV infection	People with HIV infection	People with HIV infection	People with HIV infection	—	—
Ramkumar 2018[43]	—	—	—	—	—	—	—	Several sources
Sonnenberg 2002[45]	—	—	People with HIV infection	—	—	—	—	—
Cummins 2009[46]	—	—	—	—	—	THA patients	—	—

For peer review only - http://bmjopen.bmj.com/site/about/guidelines.xhtml

Supplemental Material: Enhanced recovery following hip and knee arthroplasty

Authors	EQ-5D	15 D	QWB	SG	VAS / rating scale	TTO	AQoL	Other
Graves 2016[47]	THA patients	THA patients	—	—	—	—	THA patients with prosthesis infection	—
Merollini 2013[48]	—	THA patients	—	—	—	—	THA patients with prosthesis infection	—
Nherera 2017[49]	—	—	—	—	—	—	—	SF-36 in patients undergoing orthopaedic surgery
Bolz 2010[53]	—	THA patients	—	—	—	—	—	—

* Details of utilities obtained from expert opinion are not included in this table
15-D, 15-dimension instrument; AQoL, Assessment of Quality of Life; EQ-5D, EuroQoL five-dimension instrument; HIV, human immunodeficiency virus; SF-36, Short Form (36) Health Survey; SG, standard gamble; THA, total hip arthroplasty; TKA, total knee arthroplasty; TTO, time trade-off; QWB, Quality of Well-being scale; VAS, visual analogue scale

For peer review only

For peer review only - http://bmjopen.bmj.com/site/about/guidelines.xhtml

Supplemental Material: Enhanced recovery following hip and knee arthroplasty

Table A6. Overview of quality and sources of data used by studies included in this analysis

Authors	Sources of data: Clinical effect sizes		Baseline clinical data		Duration of primary effect		Resource use		Costs		Utilities	
	Min	Max	Min	Max	Min	Max	Min	Max	Min	Max	Min	Max
Range of possible scores[*]	*1*	*9*	*1*	*6*	*1*	*6*	*1*	*6*	*1*	*6*	*1*	*4*
Brunenberg 2005[37]	—†	—†	—†	—†	—†	—†	—†	—†	1	2	1	1
Larsen 2009[38]	—†	—†	—†	—†	—†	—†	—†	—†	1	4	1	1
McLawhorn 2016[39]	1	8	2	2	NR	NR	NR	NR	2	6	2	4
Fernandes 2017[40]	—†	—†	—†	—†	—†	—†	—†	—†	1	1	1	1
Courville 2012[41]	1	1	1	1	NR	NR	2	2	2	2	2	2
Jackson 2000[42]	8	8	2	2	NR	NR	NR	NR	4	4	2	2
Ramkumar 2018[43]	1	8	4	4	6	6	4	4	4	4	1	4‡
Sonnenberg 2002[44]	8	8	2	2	NR	NR	3	3	1	3	2	4
Marques 2015[45]	—†	—†	—†	—†	—†	—†	—†	—†	1	2	1	1
Cummins 2009[46]	8	8	3	3	NR	NR	2	2	2	4	2	2
Graves 2016[47]	1	7	1	4	NR	NR	2	6	1	1	2	4
Merollini 2013[48]	1	7	1	3	NR	NR	NR	NR	1	6	2	4
Nherera 2017[49]	2	2	4	4	NA‖	NA‖	2	2	2	2	2	2
Fusco & Turchetti, 2016[50]	3	3	2	5	3	3	2	2	2	6	2	2
Kauppila 2011[51]	—†	—†	—†	—†	—†	—†	—†	—†	1	2	1	1
Smith 2018[52]	2	2	4	4	6	6	2	2	2	2	1	1
Bolz 2010[53]	NR	NR	2	2	NR	NR	NR	NR	2	2	2	2

Note: Where a study used several data sources within each category the results are reported as a range of the minimum to maximum data quality score within each category.
* Details of the full hierarchy are available in supplementary table A2 online.
† Not applicable as study is not model-based
‡ Utilities from patients with other conditions
‖ Model time horizon limited to duration of study providing clinical effect sizes

CHEC, Consensus on Health Economic Criteria; ISPOR, International Society for Pharmacoeconomics and Outcomes Research; NA, not applicable; NR, not reported

For peer review only

For peer review only - http://bmjopen.bmj.com/site/about/guidelines.xhtml

Supplemental Material: Enhanced recovery following hip and knee arthroplasty

Table A7. Summary of findings from studies included in this analysis

Authors, country	Population	Strategy	Cost	Outcome in QALYs	ICER in cost per QALY*	Probability of cost-effectiveness (threshold in cost per QALY)
**Enhanced care pathway**						
Brunenberg, *et al.*[37] Netherlands	THA	Conventional care	USD 11 312	0.65		
		Joint Recovery Programme (pre- assessment and intensive rehabilitation)	USD 10 051	0.70		
		Increment	−1261	0.07†	Dominates	0.94 (USD 45 000)
	TKA	Conventional care	USD 12 877	0.61		
		Joint Recovery Programme (pre-operative assessment and intensive rehabilitation)	USD 8541	0.65		
		Increment	−3336	0.04†	Dominates	0.99 (USD 45 000)
Larsen, *et al.*,[38] Denmark	THA + TKA	Conventional care	DKK 90 227	0.78		
		Accelerated perioperative care and rehabilitation	DKK 71 344	0.83		
		Increment	−18 880	0.05	Dominates	0.97‡ (DKK 160 000)
	THA	Conventional care	DKK 87 657	0.75		
		Accelerated perioperative care and rehabilitation	DKK 71 768	0.84		
		Increment	−15 889	0.09	Dominates	0.98‡ (DKK 160 000)
	TKA	Accelerated perioperative care and rehabilitation	DKK 70 644	0.81		
		Conventional care	DKK 95 367	0.85		
		Increment	24 723	0.04	DKK 618 075	NR

For peer review only - http://bmjopen.bmj.com/site/about/guidelines.xhtml

Supplemental Material: Enhanced recovery following hip and knee arthroplasty

Table A7. Continued.

Authors, country	Population	Strategy	Cost	Outcome in QALYs	ICER in cost per QALY*	Probability of cost-effectiveness (threshold in cost per QALY)
**Preoperative**						
Assessment and optimization of comorbidities						
McLawhorn, *et al.*,[39] US	Morbidly obese with unilateral end-stage knee osteoarthritis					
		Immediate TKA	USD 60 453	10.83		
		Bariatric surgery, followed by TKA 2 years later	USD 84 099	12.53		
		Increment	USD 23 646	1.70	USD 13 910	0.988 (USD 100 000)
Exercise						
Fernandes, *et al.*[40] Denmark	THA + TKA	Educational package	EUR 16 313	0.61		
		Supervised neuromuscular exercise + educational package	EUR 16 181	0.66		
		Increment	EUR −132	0.04[†]	Dominates	0.84 (EUR 40 000)
Staphylococcus aureus *prophylaxis*						
Courville, *et al.*,[41] US	THA	Standard infection prevention measures without *S. aureus* screening or mupirocin decolonization	USD 24 506	0.7980		
		Preoperative nasal screening for *S. aureus* followed by mupirocin treatment for patients with positive cultures	USD 24 471	0.7983	D	
		Empirical treatment of all preoperative patients with mupirocin	USD 24 258	0.7985		
		Increment (compared to standard measures)	−248	0.0005	Dominates	NR
	TKA	Standard infection prevention measures without *S. aureus* screening or mupirocin decolonization	USD 24 667	0.6783		
		Preoperative nasal screening for *S. aureus* followed by mupirocin treatment for patients with positive cultures	USD 24 611	0.6785	D	
		Empirical treatment of all preoperative patients with mupirocin	USD 24 378	0.6787		
		Increment (compared to standard measures)	−289	0.0004	Dominates	NR

For peer review only - http://bmjopen.bmj.com/site/about/guidelines.xhtml

Supplemental Material: Enhanced recovery following hip and knee arthroplasty

Authors, country	Population	Strategy	Cost	Outcome in QALYs	ICER in cost per QALY*	Probability of cost-effectiveness (threshold in cost per QALY)
**Intraoperative**						
Avoid unnecessary blood transfusion						
Jackson, *et al.*,[42] US	THA + TKA	Usual transfusion practice	NR	NR		
		Postoperative erythrocyte recovery and transfusion	NR	NR		
		Increment	USD 53	0.00001	USD 5 700 000	NR (USD 50 000)
Ramkumar, *et al.*,[43] US	THA + TKA	No pharmacologic haemostatic agent	USD 951	8.893		
		Single dose intravenous aminocaproic acid	USD 1175	9.152	D	
		Single dose intravenous tranexamic acid	USD 460	9.339		
		Increment (compared to no pharmacologic haemostatic agent)	USD −491	0.447	Dominates	NR (USD 100 000)
Sonnenberg,[44] US	THA	Usual practice without autologous donation	USD 1395	NR		
		Autologous blood donation and transfusion	USD 1539	NR		
		Increment	144	0.0523	USD 2750	NR (USD 50 000)
Local infiltration of analgesia						
Marques, *et al.*,[45] UK	THA	Standard anaesthesia	NR	NR		
		Intraoperative local anaesthetic wound infiltration administered before wound closure in addition to standard anaesthesia	NR	NR		
		Increment	GBP −86	0.052	Dominates	0.98 (GBP 20 000)
	TKA	Standard anaesthesia	NR	NR		
		Intraoperative local anaesthetic wound infiltration administered before wound closure in addition to standard anaesthesia	NR	NR		
		Increment	GBP −77	0.009	Dominates	0.60 (GBP 20 000)
Infection prevention						
Cummins, *et al.*,[46] US	THA	Conventional cement	USD 24 100	9.439		
		Antibiotic-impregnated bone cement	USD 23 900	9.454		
		Increment	−200	0.015	Dominates	NR

For peer review only

For peer review only - http://bmjopen.bmj.com/site/about/guidelines.xhtml

Supplemental Material: Enhanced recovery following hip and knee arthroplasty

Authors, country	Population	Strategy	Cost	Outcome in QALYs	ICER in cost per QALY*	Probability of cost-effectiveness (threshold in cost per QALY)
Graves, *et al.*,[47] UK[§]	THA	No systemic antibiotics, plain cement and conventional ventilation	GBP 0[§]	0[§]		
		Systemic antibiotics, antibiotic-impregnated cement, laminar ventilation and body exhaust suit	GBP 781 075	62	D	0.01[‖] (GBP 18 000)
		No systemic antibiotics, antibiotic-impregnated cement and conventional ventilation	GBP −4 634 647	89	D	0.07[‖] (GBP 18 000)
		Systemic antibiotics, plain cement and conventional ventilation	GBP −7 226 732	101	D	0.15[‖] (GBP 18 000)
		Systemic antibiotics, antibiotic-impregnated cement, conventional ventilation and body exhaust suit	GBP −3 960 897	106	D	0.11[‖] (GBP 18 000)
		Systemic antibiotics, plain cement and laminar airflow	GBP −5 271 040	118	D	0.10[‖] (GBP 18 000)
		Systemic antibiotics, antibiotic-impregnated cement and laminar airflow	GBP −6 152 877	124	D	0.06[‖] (GBP 18 000)
		No systemic antibiotics, plain cement and laminar airflow	GBP −3 271 749	124	D	0.18[‖] (GBP 18 000)
		Systemic antibiotics, antibiotic-impregnated cement and conventional ventilation	GBP −8 325 277	147		
		Increment (compared to systemic antibiotics, plain cement and conventional ventilation)	−1 098 545	46	Dominates	0.32[e] (GBP 18 000)
Merollini, *et al.*,[48] Australia	THA	No antibiotic prophylaxis	AUD 1 517 954	−163.1		
		Antibiotic prophylaxis and laminar airflow	AUD 4 592 200	−126.9	D	
		Antibiotic prophylaxis	AUD 0[¶]	0[¶]	D	
		Antibiotic prophylaxis and antibiotic-impregnated cement	AUD −126 375	32.3		
		Increment	−126 375	32.3	Dominates	0.986 (AUD 40 000)[**]
Nherera, *et al.*,[49] UK	THA + TKA	Usual care	GBP 6713	0.115		
		Single-use negative pressure wound therapy dressings	GBP 5602	0.116		
		Increment	GBP −1049	0.0014	Dominates	0.91 (GBP 20 000)

For peer review only - http://bmjopen.bmj.com/site/about/guidelines.xhtml

Supplemental Material: Enhanced recovery following hip and knee arthroplasty

Table A7. Continued.

Authors, country	Population	Strategy	Cost	Outcome in QALYs	ICER in cost per QALY*	Probability of cost-effectiveness (threshold in cost per QALY)
**Postoperative**						
Physical therapy						
Fusco & Turchetti,[50] Italy	TKA, societal perspective	20 face-to-face rehabilitation sessions	EUR 1315	13.02		
		10 face-to-face rehabilitation sessions plus 10 telesessions	EUR 977	13.02		
		Increment	−338	0	Dominates	NR (EUR 30 000)
	TKA, Italian NHS perspective	20 face-to-face rehabilitation sessions	EUR 1124	13.02		
		10 face-to-face rehabilitation sessions plus 10 telesessions	EUR 862	13.02		
		Increment	−262	0	Dominates	NR (EUR 30 000)
Kauppila, *et al.*,[51] Finland	TKA	Multidisciplinary biopsychosocial outpatient rehabilitation programme	EUR 12 950	NR		
		Conventional orthopaedic care	EUR 11 120	NR		
		Increment	−1830	0.0192	Dominates	NR
Smith, *et al.*,[52] US		Telephone calls conveying general health messages	USD 140 700	9.783		
		Telephonic health coaching and financial incentives	USD 141 000	9.788		
		Increment	USD 300	0.005	USD 57 200	0.70 (USD 100 000)
Scheduling of follow-up						
Bolz, *et al.*,[53] Australia[††]	THA, assuming 5% revisions delayed under no follow-up[‡‡]					
		2-yearly routine follow-up	AUD 26 426 908	147 940		
		Follow-up at 3 months and 1 or 2 years	AUD 21 331 518	147 940	D	
		No follow-up	AUD 14 867 616	147 949		
		Increment	−6 463 902	9	Dominates	NR

* Strategies listed in order of increasing effectiveness; ICER presented compared to next most effective strategy that is not extended-dominated; no ICER presented for dominated or extended-dominated strategies; ICERs have been calculated where not explicitly presented in the reference, where ICERs have been presented they may differ slightly from what would be calculated from the presented figures due to rounding.
† Adjusted for baseline quality of life using regression analysis
‡ Probability of being the dominant strategy

For peer review only - http://bmjopen.bmj.com/site/about/guidelines.xhtml

Supplemental Material: Enhanced recovery following hip and knee arthroplasty

§ Total costs and outcomes for a cohort of 77 321 patients, relative to the cost of the strategy of no systemic antibiotics, plain cement and conventional ventilation
‖ Probability of being most cost-effective strategy in the model
¶ Total costs and outcomes per 30 000 primary THRs, relative to the cost of the strategy of antibiotic prophylaxis
** Cost-effectiveness ≥98.6% at typical willingness-to-pay values of AUD 40 000 to AUD 64 000
†† Total costs and outcomes for a cohort of 30 440 patients
‡‡ Four different probabilities were modelled (1%, 5%, 10%, 50%), but the direction of the results did not change between them

AUD, Australian dollar; D, dominated; DKK, Danish krone; EUR, euro; GBP, British pound; ICER, incremental cost-effectiveness ratio; NHS, National Health Service; NR, not reported; QALY, quality-adjusted life year; THA, total hip arthroplasty; TKA, total knee arthroplasty; UK, United Kingdom; US, United States; USD, United States dollar

For peer review only

For peer review only - http://bmjopen.bmj.com/site/about/guidelines.xhtml

Supplemental Material: Enhanced recovery following hip and knee arthroplasty

Table A8. Components of enhanced recovery pathway in the trials of an entire pathway

	Larsen, *et al.*[38]		Brunenberg, *et al.*[37]	
**Intervention type**	**Standard protocol**	**Accelerated protocol**	**Usual care**	**Joint recovery programme**
Pre-operative assessment	NA	NA	No standardized screening	History, examination & blood testing 6 weeks prior to surgery
Pre-operative education	Information given to patients on admission	Information provided in groups at outpatient clinic prior to hospitalization	No information session	Information session 1–2 weeks prior to surgery
Day of admission	Day before surgery	Day of surgery	NA	NA
Bed allocation in hospital	Amongst other patients	Patients having joint replacement placed together in separate part of ward	NA	NA
Staff involved	Various	One nurse in charge of multidisciplinary team of nurses, OT & PT	NA	Supervised by PT & nurses throughout admission
Nutrition	Screening	Screening plus daily intake 1.5 L fluid & 2 protein drinks	NA	NA
Start of mobilization	1 day after surgery	Day of surgery	NA	NA
Description of mobilization	Individual & gradual mobilization according to patient tolerance	Intensive mobilization in teams with pre-set goals	Conventional physiotherapy	Rehabilitation in groups in a room resembling the home situation
Duration of mobilization	4 hours daily	8 hours daily	1 hour daily	Duration not specified
Discharge planning	NA	NA	During admission	6 weeks prior to surgery
Other details	NA	NA		Patients could involve a family member or friend to give emotional support

NA, not applicable; OT, occupational therapists; PT, physiotherapists

For peer review only - http://bmjopen.bmj.com/site/about/guidelines.xhtml

Supplemental Material: Enhanced recovery following hip and knee arthroplasty

Table A9. Willingness to pay thresholds and incremental cost-effectiveness ratios for all studies converted into international dollars

		Values as reported*		Adjusted values in international dollars[†]	
**Authors, country**	**Currency, cost year**	**WTP threshold**	**ICER (unit cost/QALY)**	**WTP threshold**	**ICER (unit cost/QALY)**
Brunenberg, *et al.*[37] Netherlands	USD, 2002	45 000	—[‡]	45 000	—[‡]
Larsen, *et al.*,[38] Denmark	DKK, 2006	160 000	618 075	22 122	99 624
McLawhorn, *et al.*,[39] US	USD, 2012	100 000	13 910	100 000	14 995
Fernandes, *et al.*[40] Denmark	EUR, 2012	40 000	—[‡]	41 720	—[‡]
Courville, *et al.*,[41] US	USD, 2005	NR	—[‡]	—	—[‡]
Jackson, *et al.*,[42] US	USD, 2000[§]	50 000	5 700 000	50 000	7 787 912
Ramkumar, *et al.*,[43] US	USD, 2016	100 000	—[‡]	100 000	—[‡]
Sonnenberg,[44] US	USD, 2000	50 000	2 750	50 000	3 757
Marques, *et al.*,[45] UK	GBP, 2015[§]	20 000	—[‡]	28 838	—[‡]
Cummins, *et al.*,[46] US	USD, 2002	NR	—[‡]	—	—[‡]
Graves, *et al.*,[47] UK	GBP, 2012	18 000	—[‡]	25 954	—[‡]
Merollini, *et al.*[48], Australia	AUD, 2011	40 000	—[‡]	27 287	—[‡]
Nherera, *et al.*,[49] UK	GBP, 2015	20 000	—[‡]	28 838	—[‡]
Fusco & Turchetti,[50] Italy	EUR, 2013	30 000	—[‡]	41 482	—[‡]
Kauppila, *et al.*,[51] Finland	EUR, 2006	NR	—[‡]	—	—[‡]
Smith, *et al.*,[52] US	USD, 2016	100 000	57 200	100 000	57 200
Bolz, *et al.*,[53] Australia	AUS, 2010[§]	NR	—[‡]	—	—[‡]

* Only non-dominating results reported
† Willingness to pay thresholds not adjusted for cost year as they have been constant in most countries since they were introduced, in spite of cost inflation; ICERs additionally adjusted to 2016 values
‡ Dominance reported for all strategies
§ Cost year not reported; assumed as publication year

AUD, Australian dollar; DKK, Danish krone; EUR, euro; GBP, British pound; ICER, incremental cost-effectiveness ratio; NR, not reported; QALY, quality-adjusted life year; UK, United Kingdom; US, United States; USD, United States dollar; WTP, willingness to pay

For peer review only

For peer review only - http://bmjopen.bmj.com/site/about/guidelines.xhtml

PRISMA 2009 Checklist

Section/topic	#	Checklist item	Reported on page #
**TITLE**			
Title	1	Identify the report as a systematic review, meta-analysis, or both.	1
**ABSTRACT**			
Structured summary	2	Provide a structured summary including, as applicable: background; objectives; data sources; study eligibility criteria, participants, and interventions; study appraisal and synthesis methods; results; limitations; conclusions and implications of key findings; systematic review registration number.	2
**INTRODUCTION**			
Rationale	3	Describe the rationale for the review in the context of what is already known.	5
Objectives	4	Provide an explicit statement of questions being addressed with reference to participants, interventions, comparisons, outcomes, and study design (PICOS).	5
**METHODS**			
Protocol and registration	5	Indicate if a review protocol exists, if and where it can be accessed (e.g., Web address), and, if available, provide registration information including registration number.	6
Eligibility criteria	6	Specify study characteristics (e.g., PICOS, length of follow-up) and report characteristics (e.g., years considered, language, publication status) used as criteria for eligibility, giving rationale.	6
Information sources	7	Describe all information sources (e.g., databases with dates of coverage, contact with study authors to identify additional studies) in the search and date last searched.	6
Search	8	Present full electronic search strategy for at least one database, including any limits used, such that it could be repeated.	6 and Supplementary Table A1
Study selection	9	State the process for selecting studies (i.e., screening, eligibility, included in systematic review, and, if applicable, included in the meta-analysis).	6
Data collection process	10	Describe method of data extraction from reports (e.g., piloted forms, independently, in duplicate) and any processes for obtaining and confirming data from investigators.	6
Data items	11	List and define all variables for which data were sought (e.g., PICOS, funding sources) and any assumptions and simplifications made.	6-7
Risk of bias in individual studies	12	Describe methods used for assessing risk of bias of individual studies (including specification of whether this was done at the study or outcome level), and how this information is to be used in any data synthesis.	7
Summary measures	13	State the principal summary measures (e.g., risk ratio, difference in means).	7
Synthesis of results	14	Describe the methods of handling data and combining results of studies, if done, including measures of consistency (e.g., I^2) for each meta-analysis.	7

For peer review only

For peer review only - http://bmjopen.bmj.com/site/about/guidelines.xhtml

PRISMA 2009 Checklist

Section/topic	#	Checklist item	Reported on page #
Risk of bias across studies	15	Specify any assessment of risk of bias that may affect the cumulative evidence (e.g., publication bias, selective reporting within studies).	7
Additional analyses	16	Describe methods of additional analyses (e.g., sensitivity or subgroup analyses, meta-regression), if done, indicating which were pre-specified.	Not applicable
**RESULTS**			
Study selection	17	Give numbers of studies screened, assessed for eligibility, and included in the review, with reasons for exclusions at each stage, ideally with a flow diagram.	9-10 and Figure 1
Study characteristics	18	For each study, present characteristics for which data were extracted (e.g., study size, PICOS, follow-up period) and provide the citations.	9-10, Table 1 and Supplementary Tables A3,A4 and A5
Risk of bias within studies	19	Present data on risk of bias of each study and, if available, any outcome level assessment (see item 12).	13, Figure 2 and Table A6
Results of individual studies	20	For all outcomes considered (benefits or harms), present, for each study: (a) simple summary data for each intervention group (b) effect estimates and confidence intervals, ideally with a forest plot.	14-20, Table 2 and Supplementary Table A7
Synthesis of results	21	Present results of each meta-analysis done, including confidence intervals and measures of consistency.	Not applicable
Risk of bias across studies	22	Present results of any assessment of risk of bias across studies (see Item 15).	13, Figure 2 and Table A6
Additional analysis	23	Give results of additional analyses, if done (e.g., sensitivity or subgroup analyses, meta-regression [see Item 16]).	Not applicable
**DISCUSSION**			
Summary of evidence	24	Summarize the main findings including the strength of evidence for each main outcome; consider their relevance to key groups (e.g., healthcare providers, users, and policy makers).	20-21
Limitations	25	Discuss limitations at study and outcome level (e.g., risk of bias), and at review-level (e.g., incomplete retrieval of identified research, reporting bias).	21-22
Conclusions	26	Provide a general interpretation of the results in the context of other evidence, and implications for future research.	23
**FUNDING**			
Funding	27	Describe sources of funding for the systematic review and other support (e.g., supply of data); role of funders for the systematic review.	4

For peer review only - http://bmjopen.bmj.com/site/about/guidelines.xhtml

PRISMA 2009 Checklist

From: Moher D, Liberati A, Tetzlaff J, Altman DG, The PRISMA Group (2009). Preferred Reporting Items for Systematic Reviews and Meta-Analyses: The PRISMA Statement. PLoS Med 6(7): e1000097. doi:10.1371/journal.pmed1000097

For more information, visit: **www.prisma-statement.org**. Page 2 of 2

For peer review only

BMJ Open

Enhanced recovery following hip and knee arthroplasty: A systematic review of cost-effectiveness evidence

Journal:	*BMJ Open*
Manuscript ID	bmjopen-2019-032204.R2
Article Type:	Original research
Date Submitted by the Author:	02-Dec-2019
Complete List of Authors:	Pritchard, Mark; University of Oxford, Nuffield Department of Population Health<br>Murphy, Jacqueline; Queen Mary University of London,<br>Cheng, Lok; University of Oxford, Nuffield Department of Population Health<br>Janarthanan, Roshni; University of Oxford, Nuffield Department of Population Health<br>Judge, Andrew; University of Bristol, Musculoskeletal Research Unit, Translational Health Sciences, Bristol Medical School, University of Bristol, Learning and Research Building, Level 1, Southmead Hospital, Southmead, BS10 5NB<br>Leal, Jose; University of Oxford, Nuffield Department of Population Health
**Primary Subject Heading**:	Health economics
Secondary Subject Heading:	Surgery
Keywords:	Systematic review, hip replacement, knee replacement, osteoarthritis, economic evaluation, cost-effectiveness

SCHOLARONE™
Manuscripts

Enhanced recovery following hip and knee arthroplasty: A systematic review of cost-effectiveness evidence

Mark G Pritchard, MB ChB, MSc,[1,2,*] markgpritchard@gmail.com, ORCID iD 0000-0003-1726-8989

Jacqueline Murphy, MMath,[1,*] jacqueline.murphy@ndph.ox.ac.uk, ORCID iD 0000-0002-3927-7002

Lok Yin Cheng, MBChB, BSc,[1,3] lok.cheng@nhs.net

Roshni Janarthanan MBBS,[1,2] roshni.janarthanan@medsci.ox.ac.uk

Andrew Judge, PhD,[4,5] andrew.judge@bristol.ac.uk, ORCID iD 0000-0003-3015-0432

José Leal, DPhil,[1] jose.leal@ndph.ox.ac.uk, ORCID iD 0000-0001-7870-6730

1 Health Economics Research Centre, Nuffield Department of Population Health, University of Oxford, Oxford, United Kingdom

2 Oxford University Hospitals NHS Foundation Trust, John Radcliffe Hospital, Oxford, United Kingdom

3 Stoke Mandeville Hospital, Buckinghamshire Healthcare NHS Trust, Aylesbury, Buckinghamshire, United Kingdom

4 Nuffield Department of Orthopaedics, Rheumatology and Musculoskeletal Sciences (NDORMS), Centre for Statistics in Medicine, University of Oxford, Oxford, United Kingdom

5 Musculoskeletal Research Unit, Translational Health Sciences, Bristol Medical School, University of Bristol, Bristol, United Kingdom

* JM and MGP contributed equally to this study.

Correspondence to: Dr José Leal, Health Economics Research Centre, Nuffield Department of Population Health, University of Oxford, Old Road Campus, Oxford OX3 7LF, United Kingdom.

Telephone: 01865 289 263. jose.leal@dph.ox.ac.uk

Word count: 4055

For peer review only

For peer review only - http://bmjopen.bmj.com/site/about/guidelines.xhtml

Structured Abstract

Objectives: To assess cost-effectiveness of enhanced recovery pathways following total hip and knee arthroplasties. Secondary objectives were to report on quality of studies and identify research gaps for future work.

Design: Systematic review of cost–utility analyses.

Data Sources: Ovid MEDLINE, Embase, the National Health Service Economic Evaluations Database, and EconLit, January 2000 to August 2019.

Eligibility Criteria: English-language peer-reviewed cost–utility analyses of enhanced recovery pathways, or components of one, compared to usual care, in patients having total hip or knee arthroplasties for osteoarthritis.

Data extraction and synthesis: Data extracted by three reviewers with disagreements resolved by a fourth. Study quality assessed using the Consensus on Health Economic Criteria (CHEC) list, the International Society for Pharmacoeconomics and Outcomes Research (ISPOR) and Assessment of the Validation Status of Health-Economic (AdViSHE) decision models tools; for trial-based studies the Cochrane Collaboration's tool to assess risk of bias. No quantitative synthesis was undertaken.

Results: We identified 17 studies: five trial-based and 12 model-based. Two analyses evaluated entire enhanced recovery pathways and reported these to be cost effective compared to usual care. Eleven pathway components were more effective and cost-saving compared to usual care, two were cost-effective, and two were not cost-effective. We had concerns around risk of bias for all included studies, particularly regarding the short time horizon of the trials and lack of reporting of model validation.

Conclusions: Consistent results supported enhanced recovery pathways as a whole, prophylactic systemic antibiotics, antibiotic-impregnated cement and conventional ventilation for infection prevention. No other interventions were subject of more than one study. We found ample scope for future cost-effectiveness studies, particularly analyses of entire recovery pathways and comparison of incremental changes within pathways. A key limitation is that standard practices have changed

For peer review only - http://bmjopen.bmj.com/site/about/guidelines.xhtml

over the period covered by the included studies.

Registration: International Prospective Register of Systematic Reviews: CRD42017059473

Keywords: Arthroplasty, cost-effectiveness, cost–utility, enhanced recovery, hip, knee, systematic review

For peer review only

For peer review only - http://bmjopen.bmj.com/site/about/guidelines.xhtml

ARTICLE SUMMARY

Strengths and limitations of this study

- This systematic review of enhanced recovery pathways for hip and knee arthroplasties had a detailed search strategy, including entire pathways and their components.
- Appropriate tools were used to assess quality and validity of models, trials and economic evaluations.
- Conclusions were reliant on the availability, quality and validity of published studies into cost-effectiveness of hip and knee arthroplasty.

For peer review only

Funding statement

This project was funded by the National Institute for Health Research Health Services and Delivery Research Programme (project number 14/46/02). Support was received from the Oxford NIHR Biomedical Research Centre, Nuffield Orthopaedic Centre, University of Oxford. AJ was supported by the NIHR Biomedical Research Centre at the University Hospitals Bristol NHS Foundation Trust and the University of Bristol. The views and opinions expressed herein are those of the authors and do not necessarily reflect those of the HS&DR Programme, NIHR, NHS or the Department of Health and Social Care.

Competing interests statement

The authors declare they have no competing interests.

For peer review only

For peer review only - http://bmjopen.bmj.com/site/about/guidelines.xhtml

Introduction

Hip and knee arthroplasties are common procedures: around a million of each were performed in 2011 within Organisation for Economic Co-operation and Development (OECD) countries,[1 2] and rates continue to increase.[3-6] Enhanced recovery is a multimodal approach to reduce surgical morbidity and mortality. Recognizing that factors other than surgical technique affect patient outcomes, Kehlet[7] considered how to optimize the preoperative, intraoperative and postoperative phases of patient care. These principles have been further developed specifically within the context of hip and knee arthroplasty.[8-15] Common components of an enhanced recovery pathway for hip and knee arthroplasty are listed in Box 1. A recent systematic review[16] found that enhanced recovery after hip and knee arthroplasties reduced length of stay in hospital. The authors cited a study from New Zealand[17] that found an enhanced recovery pathway to be cost saving, but the study did not include any data on cost-effectiveness.

Cost–utility analyses have become the preferred approach to inform decisions on healthcare resource allocation.[18 19] In these, the effects of treatments are measured in quality-adjusted life years (QALY): the product of health-related quality of life (anchored at 0 for death and 1 for perfect health), and the time (in years) spent experiencing that level of health. Incremental cost-effectiveness ratios (ICER) are used to compare a treatment to a less effective alternative and a threshold value used to determine whether it is cost-effective. Different countries have different thresholds for how much they are willing to pay per QALY gained.[20]

Systematic reviews of cost-effectiveness analyses have considered arthroplasty *vs* conservative management,[21 22] and specific components of enhanced recovery such as thromboprophylaxis.[23-25] However, we are not aware of any systematic reviews investigating the cost-effectiveness of a complete enhanced recovery pathway, or of most of the components. Our aim was to assess the cost-effectiveness evidence of enhanced recovery for patients having hip and knee arthroplasty. Specifically, we were interested in studies of adults having total hip arthroplasty (THA) or total knee arthroplasty (TKA) for osteoarthritis, comparing an enhanced recovery pathway

For peer review only - http://bmjopen.bmj.com/site/about/guidelines.xhtml

or components of one against usual care. Secondarily, we wanted to report study quality and identify research gaps for future work.

Methods

The complete methods are available in the published protocol,[26] registered with the International Prospective Register of Systematic Reviews, number CRD42017059473.[27] The selection of electronic databases and the search strategy were developed with an information specialist and in line with the preferred reporting items for systematic reviews and meta-analyses (PRISMA).[28] We searched Ovid MEDLINE, Embase, the National Health Service Economic Evaluations Database (via the Cochrane Library), and EconLit (via ProQuest), for English-language peer-reviewed papers published between 1 January 2000 and 1 March 2017 that included a cost–utility analysis of an enhanced recovery pathway or components of one, compared to usual care in patients having hip or knee arthroplasties (the complete search strategy for each database is presented in the Supplementary Table A1). Additional publications meeting our inclusion criteria were identified from the reference lists of the included studies. We repeated our search in August 2019 to identify more recently published studies. Our target population was adults having surgery for osteoarthritis. Studies exclusively concerning populations with other indications for surgery were excluded. We included studies with patients having different indications for surgery if the majority had osteoarthritis, or if the presented results allowed independent extraction of data for the subpopulation with osteoarthritis. Given that osteoarthritis is an indication for 92% of hip and 96% of knee arthroplasties,[29] we assumed that studies not providing details of the indication for surgery were representative of a population with osteoarthritis, and therefore included these studies. Evaluations of surgical technique or choice of implant were excluded.

Studies were independently screened based on their titles and abstracts by four reviewers (MGP, JM, LYC and JL). Full texts were obtained for studies chosen for inclusion by any reviewer. As an amendment to the protocol, evaluations of thromboprophylaxis were excluded at the full-text

For peer review only - http://bmjopen.bmj.com/site/about/guidelines.xhtml

stage due to a recent comprehensive systematic review in that area.[23] Data extraction was performed for remaining studies by three reviewers (MGP, JM and RJ), with disagreements resolved by a fourth reviewer (JL). The data extraction proforma is included in the published protocol.[26]

To assess the quality of studies, we completed the Consensus on Health Economic Criteria (CHEC) list[30] for each publication. For model-based studies, we also used the questionnaire produced by the International Society for Pharmacoeconomics and Outcomes Research (ISPOR),[31] and the Assessment of the Validation Status of Health-Economic (AdViSHE) decision models tool.[32] We added the Cochrane Collaboration's tool[33] to the original protocol to assess risk of bias in trial-based studies, referring to the original reports of trial outcomes where necessary. We assessed the quality of the data sources used in the studies according to a pre-specified hierarchy of evidence tool (see Supplementary Table A2).[34 35] For each component of a study, a score of 1 represented the use of the most appropriate data source, with increasing numbers representing progressively less appropriate sources.

The principal outcomes were a point estimate of cost-effectiveness in terms of incremental cost per QALY gained, and the probability of an intervention being cost effective according to the willingness to pay threshold used by the authors of each study. We also examined whether an intervention could be cost-effective in a different country or setting than the original study. Hence, we assumed the studies to be generalizable and the ICERs to be comparable after conversion into 2016 purchasing power parity international dollars (US PPP).[36] As the studies had different methodologies and looked at different combinations of components of the enhanced recovery pathway, no quantitative synthesis of the study results was attempted.

For peer review only - http://bmjopen.bmj.com/site/about/guidelines.xhtml

Box 1. Suggested components of an enhanced recovery pathway for hip and knee arthroplasty patients, data from references cited in text.

- Preoperative
  - Education
  - Discharge planning
  - Multidisciplinary assessment
  - Neuromuscular electrical stimulation
  - Nutrition screening
  - Optimization of comorbidities
  - Physiotherapy
  - Premedication (possibly including standardized analgesia and steroids)
  - Pulsed electromagnetic fields
- Intraoperative
  - Standardized intravenous fluids
  - Avoid unnecessary blood transfusion
  - Minimally invasive surgery
  - Reduce heat loss
  - Specified anaesthetic requirements, such as spinal anaesthesia
  - Local infiltration of anaesthesia
  - Tranexamic acid
  - Prophylactic antibiotics
  - Intravenous dexamethasone
  - Avoid unnecessary drains
  - Computer-assisted surgery
- Postoperative
  - Analgesia
    - Continuous neural block
    - Standardized multimodal analgesia, scheduled and as required
    - Reduce opioid use / avoid patient-controlled intravenous opioid analgesia
  - Scheduled anti-emetic
  - Physical therapy starting on day of surgery
  - Avoid unnecessary blood transfusion
  - Oxygen administration
  - Avoid sleep disturbances
  - Early oral nutrition
  - Wound care

For peer review only

Thromboprophylaxis<br>Neuromuscular electrical stimulation<br>Aim for early discharge

Patient and Public Involvement

No Patient involved.

Results

For peer review only

Study selection

Our original search identified 11 060 publications, and one additional publication was found from other sources. We repeated the search in August 2019 and identified 3903 additional studies. After excluding duplicates, we screened 8657 titles and abstracts. We excluded 8482 papers based on their abstracts. For one study, we were unable to obtain the full text but this did not appear to be a cost-effectiveness study from the abstract. We therefore reviewed 174 full texts. We excluded 157 studies following review of their full texts (reasons for exclusion given in Figure 1), and included seventeen papers in this review.[37-53]

Overview of included studies

Table 1 summarises included studies. Eight studies included both THA and TKA, [37 38 40-43 45 49] five only THA,[44 46-48 53] and four only TKA.[39 50-52] Two papers looked at an entire enhanced recovery pathway.[37 38] We identified cost–utility evaluations of optimization of comorbidities (specifically morbid obesity),[39] measures to reduce allogenic blood transfusion,[42-44] local infiltration of anaesthetic,[45] prophylactic antibiotics and other infection prevention measures,[41 46-49] and physical therapy before[40] or after surgery. [40 50-52] The final study[53] concerned the optimal timing of follow-up which, although not included in our prior list of enhanced recovery interventions, we have included here as being allied to the pathway. Five studies were trial-based economic evaluations,[37 38 40 45 51] the

For peer review only - http://bmjopen.bmj.com/site/about/guidelines.xhtml

remaining twelve being model-based. Eight studies were from Europe,[37 38 40 45 47 49-51] seven from the United States,[39 41-44 46 52] and two from Australia.[48 53] Perspective, time horizon, discount rates and price year used in each study are reported in Supplementary Table A3. The study perspective varied from only hospital costs [42-44 46 48 52 53] to a broader (societal) cost perspective.[37-41 49 50] Types of costs captured in each study are reported in Supplementary Table A4. Of the seven studies eliciting utilities, QALYs were informed by utility values based on the EQ-5D-3L instrument in six [37 40 45 50 52 54] and on the 15D instrument in one (Supplementary Table A5).[51]

For peer review only

Table 1. Summary of studies included in this analysis

Authors, publication year	Comparison	Joint	Study type	Country
**Enhanced recovery pathway**				
Brunenberg, *et al.* 2005[37]	Joint Recovery Programme (pre- assessment and intensive rehabilitation), *vs* conventional care	Hip and knee	Trial-based	Netherlands
Larsen, *et al.*, 2009[38]	Accelerated perioperative care and rehabilitation, *vs* conventional care	Hip and knee	Trial-based	Denmark
**Preoperative**				
McLawhorn, *et al.*, 2016[39]	Bariatric surgery followed by TKA 2 years later, *vs* immediate TKA	Knee	Markov model	US
Fernandes, *et al.*, 2017[40]	Supervised neuromuscular exercise and an educational package, *vs* educational package alone	Hip and knee	Trial-based	Denmark
Courville, *et al.*, 2012[41]	Preoperative nasal screening for *S. aureus* colonization followed by mupirocin treatment for patients with positive cultures, *vs* empirical treatment of all preoperative patients with mupirocin, *vs* standard infection prevention measures without *S. aureus* screening or mupirocin decolonization.	Hip and knee	Decision tree model	US
**Intraoperative**				
Jackson, *et al.*, 2000[42]	Postoperative erythrocyte recovery and transfusion, *vs* usual transfusion practice	Hip and knee	Markov model	US
Ramkumar, *et al.*, 2018[43]	Single dose intravenous tranexamic acid, *vs* single dose intravenous aminocaproic acid, *vs* no pharmacologic haemostatic agent	Hip and knee	Decision-tree model	US
Sonnenberg, 2002[44]	Autologous blood donation and transfusion, *vs* usual practice without autologous donation	Hip	Markov model	US
Marques, *et al.*, 2015[45]	Intraoperative local anaesthetic wound infiltration administered before wound closure in addition to standard anaesthesia, *vs* standard anaesthesia	Hip and knee	Trial-based	UK
Cummins, *et al.*, 2009[46]	Antibiotic-impregnated bone cement, *vs* conventional cement	Hip	Markov model	US
Graves, *et al.*, 2016[47]	Nine arms, comparing combinations of prophylactic systemic antibiotics, antibiotic-impregnated cement, laminar airflow and body exhaust suits	Hip	Markov model	UK
Merollini, *et al.*, 2013[48]	No antibiotic prophylaxis, antibiotic prophylaxis and antibiotic-impregnated cement, and antibiotic prophylaxis and laminar airflow, each compared to a baseline strategy of routine antibiotic prophylaxis	Hip	Markov model	Australia
Nherera, *et al.*, 2017[49]	Single-use negative pressure wound therapy dressings, *vs* usual care	Hip and knee	Decision-tree model	UK
**Postoperative**				
Fusco & Turchetti, 2016[50]	10 face-to-face rehabilitation sessions plus 10 telesessions, *vs* 20 face-to-face rehabilitation sessions	Knee	Markov model	Italy
Kauppila, *et al.*, 2011[51]	Multidisciplinary biopsychosocial outpatient rehabilitation programme, *vs* conventional orthopaedic care	Knee	Trial-based	Finland

For peer review only - http://bmjopen.bmj.com/site/about/guidelines.xhtml

Authors, publication year	Comparison	Joint	Study type	Country
Smith, *et al.*, 2018[52]	Telephonic health coaching and financial incentives, vs telephone calls conveying general health messages	Knee	Markov model	US
Bolz, *et al.*, 2010[53]	2-yearly routine follow-up, *vs* follow-up at 3 months and 1 or 2 years, *vs* no follow-up	Hip	Markov model	Australia

NHS: National Health Service; THA: total hip arthroplasty; TJA: total joint arthroplasty; TKA: total knee arthroplasty; UKA: unicompartmental knee arthroplasty

For peer review only

Assessment of study and reporting quality

Using the CHEC list, the quality of the studies was generally good (Figure 2a). Items raising more concerns were the short time horizons of the five trial-based studies (one year)[37 38 40 45 51] and three model-based studies (between six weeks[49] and seven years[53]). Using the Cochrane Collaboration's tool (Figure 2b) for the five trials[37 54-57] studies were based on, the risk of bias was low for items such as incomplete outcome data and selective reporting but high or uncertain for the remainder. Four trial reports stated that participants were allocated at random,[54-57] and the fifth was a before and after trial whereby patients were recruited consecutively from a waiting list.[37] Larsen and colleagues[54] discussed stratification, Kauppila and colleagues[55] reported a computer-generated sequence, and Villadsen and colleagues[56] used sequentially numbered sealed opaque envelopes. The staff administering the interventions were not blinded to allocation in any trial, and in three trials,[37 55 57] outcomes were assessed by researchers aware of the treatment allocation. Using the ISPOR questionnaire, the quality of the model-based studies was generally good (Figure 2c). However, none of the model-based studies reported a detailed process for internal and external validation. Four studies[42 44 50 52] were based on previously published models.[58-61] Of these, only Briggs and colleagues[58] (the basis for Fusco and Turchetti's model[50]) provided details of model validation. Some model-based studies excluded potential important outcomes, for example, Cummins and colleagues[46] did not include the possibility of requiring more than one revision surgery, and in Bolz and colleagues' model[53] there was no reduction in utility associated with delayed revisions. Further limitations in model validation were highlighted with the AdViSHE tool (Figure 2d).[32]

The hierarchy of evidence used in the studies is reported in Supplementary Table A6. Three model-based studies[42 44 46] scored poorly as they did not use randomised trial data to inform the clinical effective size of the interventions being analysed. Most studies did not cite a source for the assumption of the duration of treatment effect beyond that observed in the primary source for clinical effect size. Four trial-based studies and one model-based study included assessments of

For peer review only - http://bmjopen.bmj.com/site/about/guidelines.xhtml

utilities from patients using validated tools and scored highly in terms of quality of evidence.

Results of economic evaluation

The results of the included studies are summarized in Table 2. Costs, QALYs and incremental cost-effectiveness ratios for each comparison performed in each study are listed in Supplementary Table A7.

For peer review only

Table 2. Summary of findings from studies included in this analysis

Authors, country	Population	Strategy	Cost-effective?
**Enhanced recovery pathway**			
Brunenberg, *et al.*[37] Netherlands	THA and TKA	Conventional care	-
		Joint Recovery Programme (pre- assessment and intensive rehabilitation)	Yes, more effective and less costly
Larsen, *et al.*,[38] Denmark	THA	Conventional care	-
		Accelerated perioperative care and rehabilitation	Yes, more effective and less costly
	TKA	Conventional care	-
		Accelerated perioperative care and rehabilitation	Yes, less effective but less costly
**Preoperative**			
McLawhorn, *et al.*,[39] US	Morbid obese TKA	Immediate TKA	-
		Bariatric surgery, followed by TKA 2 years later	Yes
Fernandes, *et al.*[40] Denmark	THA and TKA	Educational package	-
		Supervised neuromuscular exercise in addition to educational package	Yes, more effective and less costly
Courville, *et al.*,[41] US	THA and TKA	Standard infection prevention measures without *S. aureus* screening or mupirocin decolonization OR preoperative nasal screening for *S. aureus* followed by mupirocin treatment for patients with positive cultures	-
		Empirical treatment of all preoperative patients with mupirocin	Yes, more effective and less costly
**Intraoperative**			
Jackson, *et al.*,[42] US	THA and TKA	Usual transfusion practice	-
		Postoperative erythrocyte recovery and transfusion	No
Ramkumar, *et al.*,[43] US	THA and TKA	No pharmacologic haemostatic agent OR single dose intravenous aminocaproic acid	-
		Single dose intravenous tranexamic acid	Yes, more effective and less costly
Sonnenberg,[44] US	THA	Usual practice without autologous donation	-
		Autologous blood donation and transfusion	Yes
Marques, *et al.*,[45] UK	THA and TKA	Standard anaesthesia	-
		Intraoperative local anaesthetic wound infiltration administered before wound closure in addition to standard anaesthesia	Yes, more effective and less costly
Cummins, *et al.*,[46] US	THA	Conventional cement	-
		Antibiotic-impregnated bone cement	Yes, more effective and less costly
Graves, *et al.*,[47] UK[§]	THA	No systemic antibiotics, plain cement and conventional ventilation	-
		Systemic antibiotics, antibiotic-impregnated cement and conventional ventilation	Yes, more effective and less costly
Merollini, *et al.*,[48] Australia	THA	No antibiotic prophylaxis OR antibiotic prophylaxis OR antibiotic prophylaxis and laminar airflow	-
		Antibiotic prophylaxis and antibiotic-impregnated cement	Yes, more effective and less costly
Nherera, *et al.*,[49] UK	THA and TKA	Usual care	-
		Single-use negative pressure wound therapy dressings	Yes, more effective and less costly
**Postoperative**			
Fusco &	TKA	20 face-to-face rehabilitation sessions	-

For peer review only - http://bmjopen.bmj.com/site/about/guidelines.xhtml

Authors, country	Population	Strategy	Cost-effective?
Turchetti,[50] Italy		10 face-to-face rehabilitation sessions plus 10 telesessions	Yes, same effectiveness but less costly
Kauppila, *et al.*,[51] Finland	TKA	Conventional orthopaedic care	-
		Multidisciplinary biopsychosocial outpatient rehabilitation programme	No
Smith, *et al.*,[52] US	TKA	Telephone calls conveying general health messages	-
		Telephonic health coaching and financial incentives to increase physical activity	Yes
Bolz, *et al.*,[53] Australia	THA	2-yearly routine follow-up OR follow-up at 3 months and 1 or 2 years	-
		No follow-up	Yes

Whole recovery pathway

Two studies considered multiple components of the enhanced recovery pathway. The first study was an economic analysis of a randomized trial with 56 THA and 31 TKA participants in Denmark.[38] The pathway used in the treatment arm ("accelerated care") had no difference from the control group ("conventional rehabilitation") in terms of intraoperative management, analgesia, nausea control or bowel regulation. Differences in the treatment protocols between the two arms involved patient education, nutrition, admission times, staffing, and mobilization (described in Supplementary Table A8). The accelerated care pathway was the dominant strategy both overall and in the subgroup of THA patients (i.e. less costly and more effective than the control group). For TKA patients, the authors found the accelerated care pathway to be cost-saving but less effective compared to the control group although not statistically significant. There was a cost saving of 618 075 Danish krone per QALY lost with accelerated care compared to conventional rehabilitation, which made it cost-effective (threshold of 160 000 krone per QALY in Denmark).

The second study was an economic evaluation of a before and after trial with 98 THA and 62 TKA participants in The Netherlands.[37] The intervention ('Joint Recovery Programme') consisted of a 20-minute pre-assessment screening 6 weeks before the operation for physical assessment and analysis of the home situation to aid discharge planning, patient education sessions one to two weeks before surgery; group rehabilitation sessions, and supervision by physical therapists and

For peer review only - http://bmjopen.bmj.com/site/about/guidelines.xhtml

nurses (Supplementary Table A7). Patients in the 'usual care' group underwent conventional physiotherapy for one hour daily, did not receive pre-assessment screening or information sessions, and discharge arrangements were addressed during admission to hospital. The joint recovery programme intervention was dominant for both hip and knee replacement, resulting in a cost saving of 1261 United States dollars (USD) per patient for THA and 3336 USD per patient for TKA, with no statistically significant difference in effect. The probability that the joint recovery programme was the most cost-effective option was above 80% for THA and TKA for willingness to pay thresholds up to 45 000 USD.

Preoperative components

McLawhorn and colleagues[39] used a Markov model to assess the cost-effectiveness of bariatric surgery two years before TKA for morbidly obese (body mass index ≥35 kg/m^2) patients who were candidates for both operations due to end-stage knee osteoarthritis and failed non-operative weight-loss interventions. The strategy including bariatric surgery was cost-effective at the stated willingness to pay threshold of 100 000 USD per QALY in 98.8% of probabilistic simulations.

Fernandes and colleagues[40] conducted an economic evaluation alongside a trial of 8 weeks of supervised neuromuscular exercise in addition to an educational package prior to surgery. Their point estimate was that the intervention was dominant, with a saving of 132 Euro and a benefit of 0.04 QALYs. It had an 84% probability of being cost effective at a willingness to pay threshold of 40 000 Euro per QALY.

Courville and colleagues[41] compared three strategies of screening for and treating *Staphylococcus aureus* colonization to prevent deep surgical site infections for TKA and THA. They found that decolonization of all preoperative patients with mupirocin, without testing for *S. aureus*, was the dominant strategy (cheaper and more effective) when compared to treating only patients testing positive for *S. aureus*, or no screening or decolonization for *S. aureus*.

For peer review only - http://bmjopen.bmj.com/site/about/guidelines.xhtml

Intraoperative components

Strategies to reduce allogenic blood transfusions were collection of autologous blood prior to surgery,[44] aseptic collection of wound drainage,[42] and use of aminocaproic acid or tranexamic acid to reduce bleeding.[43] Autologous blood collection was found to be cost-effective at 2750 USD per QALY gained, whereas wound drainage collection was not, costing 5.7 million USD per QALY gained. However, in the latter study, the only benefit of avoiding allogenic transfusion considered was a reduced risk of blood-borne virus infection.[42] In contrast, Sonnenberg's model for THA[44] found a minimal effect from the risk of blood-borne virus infection in either costs or outcomes: 99.6% of the increase in QALYs was due to a reduced risk of bacterial infection. When the risk of bacterial infection was removed from this model, the ICER increased to 2.5 million USD per QALY gained. Use of tranexamic acid was more effective and cost-saving compared to either aminocaproic acid or not using a haemostatic agent.[43]

Marques and colleagues conducted economic evaluations[45] alongside two randomized controlled trials of adding local wound infiltration with bupivacaine to usual anaesthetic care for THA and TKA.[57] The infiltration of local anaesthetic was found to be dominant compared to standard anaesthesia in both THA and TKA patients.

Three studies used Markov models to investigate similar measures to reduce surgical site infection in the United States,[46] United Kingdom,[47] and Australia.[48] The dominant strategy in all three included use of antibiotic-impregnated cement. The two studies that looked at other factors[47] [48] each found use of prophylactic systemic antibiotics to be dominant over non-use, and use of conventional ventilation in operating theatres to be dominant over laminar airflow ventilation. Graves and colleagues[47] considered use of body exhaust suits, and found them to be dominated by strategies that did not include use of these suits. A fourth study investigated using single use negative pressure wound dressings to reduce surgical site infection.[49] This was found to be dominant compared with usual care.

For peer review only - http://bmjopen.bmj.com/site/about/guidelines.xhtml

Postoperative components

Fusco and Turchetti[50] used a Markov model to evaluate a strategy of ten face-to-face rehabilitation sessions followed by ten telerehabilitation sessions after TKA, compared to twenty face-to-face sessions. They found the strategy including telerehabilitation to be cost saving, and improved range of movement (knee flexion). However, they found no utility data for patients following a telerehabilitation programme so for their base case assumed it to be non-inferior to face-to-face rehabilitation. In a sensitivity analysis, if telerehabilitation conferred an improvement in quality of life of at least 2.5%, the strategy's probability of being cost-effective was 1; if it led to a reduction in quality of life of at least 2.5%, the probability of being cost-effective was 0 (each at a willingness to pay threshold of 30 000 euros per QALY).

Kauppila and colleagues[51] performed an economic evaluation of a ten-day outpatient rehabilitation course between two and four months after TKA that included clinical assessments, physical activity, sessions with a psychologist, and lectures from an orthopaedic surgeon, nutritionist and social worker.[55] They found that patients who completed this course had higher costs and slightly worse quality of life outcomes over the 1 year of follow-up (though the difference was non-significant at the 5% level) compared to those receiving conventional orthopaedic care.

Smith and colleagues[52] used a Markov model to investigate a post-operative strategy combining telephonic health coaching and financial incentives to increase physical activity. The intervention increased costs by 300 US dollars and was associated with an increase in utility of 0.005 QALYs. The point-estimate ICER was 57 200 dollars per QALY, with a 70% probability of being cost effective at a willingness to pay threshold of 100 000 US dollars per QALY.

Bolz and colleagues[53] compared three follow-up strategies: two-yearly routine follow-up; follow-up twice (at three months, and between one and two years after surgery); or no follow-up. The model assumed no revisions would be delayed in either strategy that included follow-up, and the outcomes for these two strategies were identical in each analysis. The no follow-up strategy was dominant for any assumed rate of delayed revision between 1% and 50%.

For peer review only - http://bmjopen.bmj.com/site/about/guidelines.xhtml

Effects of standardizing currencies and price years

Most studies showed one strategy to be dominant (i.e. cheaper and more effective) over the others[37 38 40 41 43 45-51 53] and were therefore not affected by changes in currency or price year. When converted into 2016 US PPP, willingness to pay thresholds ranged between 22 112 (Denmark) and 100 000 dollars (US; Supplementary Table A9). Autologous blood transfusion[44] and bariatric surgery[39] would be cost-effective across all willingness to pay thresholds identified in the review. The capture and replacement of red cells[42] cost 7.8 million US PPP dollars per QALY gained and would not be cost-effective by any study's thresholds. Telephonic health coaching and financial incentives to increase physical activity[52] were cost-effective at a willingness to pay of 100 000 dollars per QALY but not with any lower threshold. Accelerated care pathway in TKA patients in Denmark[38] would not be cost-effective in a US setting using a willingness to pay of 100 000 dollars per QALY.

Discussion

Summary of evidence

Our objective was to assess the cost-effectiveness evidence of enhanced recovery following THA or TKA through a systematic collection of published cost–utility data. Previous systematic reviews considered effectiveness[16] and patient satisfaction,[62] but we believe this is the first systematic review assessing the cost-effectiveness of enhanced recovery for THA or TKA patients. We identified two cost–utility analyses of an entire pathway. This is consistent with reviews of cost-effectiveness of enhanced recovery programmes for other surgical sites,[63-65] which have found few studies reported the effect on quality of life and none presented cost-effectiveness results using QALYs. Both studies that considered cost-effectiveness of an entire recovery pathway were trials in both TKA and THA patients.[37 38] The enhanced recovery pathway was found to be associated with reduced costs for all patients and the incremental cost-effectiveness estimate favoured the

For peer review only - http://bmjopen.bmj.com/site/about/guidelines.xhtml

enhanced recovery protocol, with a high probability of being the most cost-effective option.

We identified 15 studies presenting cost–utility data for components of an enhanced recovery pathway. These studies covered only a few of the potential enhanced recovery pathway components and were conducted across different healthcare systems using used different cost perspectives. Three studies investigated overlapping strategies for reducing surgical infections supporting the use of prophylactic systemic antibiotics, antibiotic-impregnated cement, and conventional ventilation.[46-48] No other interventions were examined by more than one study. Scope to combine or generalize results is therefore limited.

The studies identified in this review were generally of good quality according to the CHEC list[30] with a short time horizon identified as a key limitation in nine studies (between 6 weeks and 7 years). This is of significant concern as short time horizons will not capture or model the impact of the interventions on costs and benefits accruing over the long post-acute care period of interest. Furthermore, we also found that the large majority of studies did not consider ethical aspects and distributional implications of their findings. When the models were assessed against the ISPOR questionnaire,[31] there were concerns about the lack of model validation work, potentially questioning the reliability of 10 of the 12 studies identified. The trials were generally of good quality. One trial was a non-randomized before and after trial,[37] and participants were not blinded to the intervention for postoperative interventions in any of the studies. However, we concluded that the overall risk of bias among the studies appeared low.

Limitations of this review

We may have missed relevant evidence by limiting our search to reports published in the English language and excluding studies that did not report QALYs. Cost–utility analyses enable comparison between different interventions and are the preferred intelligence for healthcare allocation decisions.[18 19] We therefore felt justified in limiting our review to studies reporting QALYs. We identified one study excluded due to not reporting QALYs that investigated a complete enhanced

For peer review only - http://bmjopen.bmj.com/site/about/guidelines.xhtml

recovery pathway.[66] In this study, the recovery pathway was found to be cost-saving and associated with statistically significant differences in knee flexion and extension at six months. However, the clinical significance of these differences was not discussed and no health-related quality of life data were collected.[66]

A second limitation is that standard practices have changed during the period covered by the included studies. For example, many enhanced recovery techniques are now standard practice,[8] and Nherera and colleagues[49] even included enhanced recovery pathway as usual care in their model. Furthermore, other practices in the included studies are now outdated. For example, patients in the control group of Brunenberg and colleagues' trial had had an in-patient stay of 9.4 days,[37] whereas by 2015, the mean length of stay in England was 4.9 days for TKA and 5.4 days for THA[8] and has further decreased since.[67] Also, practices such as blood transfusion rates have changed greatly: the two studies included in this review assumed transfusion rates of 89%[44] to 100%,[42] whereas rates in 2014 were 22% for THA and 18% for TKA patients,[68] and rates of infection from blood transfusion are now lower than those used in the models.[69]

Research gaps for future work

Use of prophylactic systemic antibiotics and antibiotic-impregnated cement, and thromboprophylaxis[23-25] are the only measures investigated in more than one cost–utility analysis. There is therefore very wide scope for further investigation of components of enhanced recovery. Pathways have a 'whole package' benefit in excess of the sum of their parts and can be used to ensure dissemination of evidence-based practice and delivery of more consistent care.[70-72] From this perspective, specific components within a pathway might be less important than the degree of compliance with a protocol. However, as the use of care pathways becomes more prevalent, optimizing their components will be the next step in improving outcomes.

For peer review only - http://bmjopen.bmj.com/site/about/guidelines.xhtml

Conclusions

There is limited cost–utility evidence, either for an entire enhanced recovery pathway or for individual components of a pathway, for patients having THA or TKA. There are also concerns regarding the ability of short time horizons in trials in this area to capture relevant outcomes, and regarding a general lack of reporting of model validation. Our findings support the use of enhanced recovery pathways as a whole, prophylactic systemic antibiotics, antibiotic-impregnated cement and conventional ventilation. No other interventions were assessed by more than one study. Those single studies supported use of empirical preoperative *S. aureus* decolonization of all patients with mupiricin, single dose intravenous tranexamic acid, wound infiltration with local anaesthetic, single-use negative pressure wound therapy dressings, bariatric surgery for morbidly obese patients requiring TKA, use of telerehabilitation, and telephonic health coaching with financial incentives to increase physical activity. However, we were unable to conclude how transferable these findings would be into other healthcare systems. There is ample scope for future cost-effectiveness studies into enhanced recovery for THA and TKA patients. In particular, we recommend analysis of entire enhanced recovery pathways and comparison of incremental changes of components within pathways rather than considering aspects of care in isolation.

ACKNOWLEDGEMENTS

We are grateful to Nia Roberts, information specialist, and Rafael Pinedo-Villanueva, senior research associate in health economics, both at the University of Oxford, for their assistance with the search strategy.

AUTHOR STATEMENT:

JM, AJ and JL designed the study and all authors defined the inclusion criteria. JM, LYC and JL developed the search strategies. JM and LYC piloted the search strategy and inclusion criteria. JM and JL developed the data extraction pro forma. JM, LYC and JL screened the studies. JM, MGP and

For peer review only - http://bmjopen.bmj.com/site/about/guidelines.xhtml

RJ extracted the data. MGP wrote the draft of the manuscript. JM and JL provided critical revisions to the article and all authors approved the final version of the article to be published. JL is the guarantor of the review.

DATA STATEMENT:

All data relevant to the study are included in the article or uploaded as supplementary information.

PATIENT CONSENT:

Not required.

PROVENANCE AND PEER REVIEW

Not commissioned; externally peer reviewed.

For peer review only

For peer review only - http://bmjopen.bmj.com/site/about/guidelines.xhtml

FIGURE LEGENDS:

Figure 1. Flow diagram of studies included in this review and reasons for exclusion, modified from Moher, *et al*.[28] (NHS EED, National Health Service Economic Evaluations Database; QALY, quality-adjusted life year)

Figure 2. Assessments of study quality based on tools from (*a*) Consensus on Health Economic Criteria,[30] (*b*) International Society for Pharmacoeconomics and Outcomes Research,[31] (*c*) Assessment of the Validation Status of Health-Economic decision models tool,[32] (*d*) Cochrane Collaboration.[33] Note that the study by Brunenberg and colleagues[37] was a non-randomised before and after trial and we have included it in Figure 2*d* for completeness.

For peer review only

References

1. Pabinger C, Geissler A. Utilization rates of hip arthroplasty in OECD countries. *Osteoarthritis and cartilage* 2014;22(6):734-41. doi: 10.1016/j.joca.2014.04.009
2. Pabinger C, Lothaller H, Geissler A. Utilization rates of knee-arthroplasty in OECD countries. *Osteoarthritis and Cartilage* 2015;23(10):1664-73. doi: 10.1016/j.joca.2015.05.008
3. Ackerman IN, Bohensky MA, de Steiger R, et al. Lifetime risk of primary total hip replacement surgery for osteoarthritis from 2003 to 2013: A multinational analysis using national registry data. *Arthritis Care & Research* 2017;69(11):1659-67. doi: 10.1002/acr.23197
4. Ackerman IN, Bohensky MA, de Steiger R, et al. Substantial rise in the lifetime risk of primary total knee replacement surgery for osteoarthritis from 2003 to 2013: an international, population-level analysis. *Osteoarthritis Cartilage* 2017;25(4):455-61. doi: 10.1016/j.joca.2016.11.005 [published Online First: 2016/11/20]
5. Culliford DJ, Maskell J, Kiran A, et al. The lifetime risk of total hip and knee arthroplasty: results from the UK general practice research database. *Osteoarthritis Cartilage* 2012;20(6):519-24. doi: 10.1016/j.joca.2012.02.636 [published Online First: 2012/03/08]
6. Culliford D, Maskell J, Judge A, et al. Future projections of total hip and knee arthroplasty in the UK: results from the UK Clinical Practice Research Datalink. *Osteoarthritis Cartilage* 2015;23(4):594-600. doi: 10.1016/j.joca.2014.12.022 [published Online First: 2015/01/13]
7. Kehlet H. Multimodal approach to control postoperative pathophysiology and rehabilitation. *British journal of anaesthesia* 1997;78(5):606-17. doi: 10.1093/bja/78.5.606
8. Nagra NS, Hamilton TW, Strickland L, et al. Enhanced recovery programmes for lower limb arthroplasty in the UK. *Annals of the Royal College of Surgeons of England* 2017:1-6. doi: 10.1308/rcsann.2017.0124 [published Online First: 2017/08/05]
9. Auyong DB, Allen CJ, Pahang JA, et al. Reduced length of hospitalization in primary total knee arthroplasty patients using an updated enhanced recovery after orthopedic surgery (ERAS) pathway. *The Journal of arthroplasty* 2015;30(10):1705-09. doi: 10.1016/j.arth.2015.05.007
10. Maempel J, Walmsley P. Enhanced recovery programmes can reduce length of stay after total knee replacement without sacrificing functional outcome at one year. *The Annals of The Royal College of Surgeons of England* 2015;97(8):563-67. doi: 10.1308/rcsann.2015.0016
11. Dwyer AJ, Thomas W, Humphry S, et al. Enhanced recovery programme for total knee replacement to reduce the length of hospital stay. *Journal of Orthopaedic Surgery* 2014;22(2):150-54. doi: 10.1177/230949901402200206
12. Stowers MD, Lemanu DP, Coleman B, et al. Review article: Perioperative care in enhanced recovery for total hip and knee arthroplasty. *Journal of Orthopaedic Surgery* 2014;22(3):383-92. doi: 10.1177/230949901402200324
13. Ibrahim MS, Alazzawi S, Nizam I, et al. An evidence-based review of enhanced recovery interventions in knee replacement surgery. *Annals of the Royal College of Surgeons of England* 2013;95(6):386-9. doi: 10.1308/003588413x13629960046435 [published Online First: 2013/09/13]
14. McDonald D, Siegmeth R, Deakin A, et al. An enhanced recovery programme for primary total knee arthroplasty in the United Kingdom—follow up at one year. *The Knee* 2012;19(5):525-29. doi: 10.1016/j.knee.2011.07.012
15. Malviya A, Martin K, Harper I, et al. Enhanced recovery program for hip and knee replacement reduces death rate: A study of 4,500 consecutive primary hip and knee replacements. *Acta Orthopaedica* 2011;82(5):577-81. doi: 10.3109/17453674.2011.618911
16. Zhu S, Qian W, Jiang C, et al. Enhanced recovery after surgery for hip and knee arthroplasty: a systematic review and meta-analysis. *Postgraduate Medical Journal* 2017;93(1106):736-42. doi: 10.1136/postgradmedj-2017-134991
17. Stowers MDJ, Manuopangai L, Hill AG, et al. Enhanced Recovery After Surgery in elective hip and knee arthroplasty reduces length of hospital stay. *ANZ Journal of Surgery* 2016;86(6):475-79.

For peer review only - http://bmjopen.bmj.com/site/about/guidelines.xhtml

doi: 10.1111/ans.13538

18. National Institute for Health and Care Excellence. Guide to the methods of technology appraisal 2013 2013 [Available from: https://www.nice.org.uk/process/pmg9/chapter/foreword accessed September 6 2017.
19. Weinstein MC, Siegel JE, Gold MR, et al. Recommendations of the panel on cost-effectiveness in health and medicine. *JAMA* 1996;276(15):1253-58. doi: 10.1001/jama.1996.03540150055031
20. Schwarzer R, Rochau U, Saverno K, et al. Systematic overview of cost-effectiveness thresholds in ten countries across four continents. *Journal of comparative effectiveness research* 2015;4(5):485-504. doi: 10.2217/cer.15.38 [published Online First: 2015/10/23]
21. Nwachukwu BU, Bozic KJ, Schairer WW, et al. Current status of cost utility analyses in total joint arthroplasty: a systematic review. *Clinical orthopaedics and related research* 2015;473(5):1815-27. doi: 10.1007/s11999-014-3964-4 [published Online First: 2014/10/01]
22. Daigle ME, Weinstein AM, Katz JN, et al. The cost-effectiveness of total joint arthroplasty: a systematic review of published literature. *Best practice & research Clinical rheumatology* 2012;26(5):649-58. doi: 10.1016/j.berh.2012.07.013 [published Online First: 2012/12/12]
23. Brockbank J, Wolowacz S. Economic evaluations of new oral anticoagulants for the prevention of venous thromboembolism after total hip or knee replacement: A systematic review. *Pharmacoeconomics* 2017;35:517-35. doi: 10.1007/s40273-017-0486-4
24. Kapoor A, Chuang W, Radhakrishnan N, et al. Cost effectiveness of venous thromboembolism pharmacological prophylaxis in total hip and knee replacement: A systematic review. *Pharmacoeconomics* 2010;28(7):521-38. doi: 10.2165/11535210-000000000-00000
25. Wolowacz S, Hess N, Brennan V, et al. Cost-effectiveness of venous thromboembolism prophylaxis in total hip and knee replacement surgery: the evolving application of health economic modelling over 20 years. *Current medical research and opinion* 2008;24(10):2993-3006. doi: 10.1185/03007990802443255
26. Murphy J, Pritchard MG, Cheng LY, et al. Cost-effectiveness of enhanced recovery in hip and knee replacement: a systematic review protocol. *BMJ Open* 2018;8(3):e019740. doi: https://dx.doi.org/10.1136/bmjopen-2017-019740
27. Murphy J, Cheng, LY., Leal, J. Cost-effectiveness and quality of life of enhanced recovery in hip and knee replacement: a systematic review protocol. PROSPERO 2017:CRD42017059473 Available from http://www.crd.york.ac.uk/PROSPERO/display_record.asp?ID=CRD42017059473. PROSPERO International prospective register of systematic reviews, 2017.
28. Moher D, Liberati A, Tetzlaff J, et al. Preferred reporting items for systematic reviews and meta-analyses: the PRISMA statement. *PLoS medicine* 2009;6(7):e1000097. doi: 10.1371/journal.pmed.1000097
29. National Joint Registry. 13th annual report 2016: National Joint Registry for England, Wales, Northern Ireland and the Isle of Man surgical data to 31 December 2015. Hemel Hempstead, UK: National Joint Registry, 2016.
30. Evers S, Goossens M, de Vet H, et al. Criteria list for assessment of methodological quality of economic evaluations: Consensus on Health Economic Criteria. *International journal of technology assessment in health care* 2005;21(2):240-5. [published Online First: 2005/06/01]
31. Caro JJ, Eddy DM, Kan H, et al. Questionnaire to assess relevance and credibility of modeling studies for informing health care decision making: an ISPOR-AMCP-NPC Good Practice Task Force report. *Value in health* 2014;17(2):174-82. doi: 10.1016/j.jval.2014.01.003.
32. Vemer P, Corro Ramos I, van Voorn GA, et al. AdViSHE: A validation-assessment tool of health-economic models for decision makers and model users. *Pharmacoeconomics* 2016;34(4):349-61. doi: 10.1007/s40273-015-0327-2 [published Online First: 2015/12/15]
33. Higgins JP, Altman DG, Gøtzsche PC, et al. The Cochrane Collaboration's tool for assessing risk of bias in randomised trials. *BMJ (Clinical research ed)* 2011;343:d5928. doi:

For peer review only - http://bmjopen.bmj.com/site/about/guidelines.xhtml

10.1136/bmj.d5928 [published Online First: 2011/10/20]
34. Cooper N, Coyle D, Abrams K, et al. Use of evidence in decision models: an appraisal of health technology assessments in the UK since 1997. *Journal of Health Services Research & Policy* 2005;10(4):245-50. doi: 10.1258/135581905774414187
35. Coyle D, Lee KM. Evidence-based economic evaluation: how the use of different data sources can impact results. In: Donaldson C, Mugford M, Vale L, eds. Evidence-based health economics: from effectiveness to efficiency in systematic review. London, UK: BMJ Publishing Group 2002:55-66.
36. Organisation for Economic Co-operation and Development. Prices and purchasing power parities 2017 [Available from: http://stats.oecd.org/Index.aspx?DataSetCode=CPL accessed September 19 2017.
37. Brunenberg DE, van Steyn MJ, Sluimer JC, et al. Joint recovery programme versus usual care: an economic evaluation of a clinical pathway for joint replacement surgery. *Medical care* 2005;43(10):1018-26. [published Online First: 2005/09/17]
38. Larsen K, Hansen TB, Thomsen PB, et al. Cost-effectiveness of accelerated perioperative care and rehabilitation after total hip and knee arthroplasty. *Journal of Bone and Joint Surgery American volume* 2009;91(4):761-72. doi: 10.2106/JBJS.G.01472
39. McLawhorn AS, Southren D, Wang YC, et al. Cost-effectiveness of bariatric surgery prior to total knee arthroplasty in the morbidly obese: A computer model-based evaluation. *Journal of Bone & Joint Surgery - American Volume* 2016;98(2):e6. doi: 10.2106/JBJS.N.00416
40. Fernandes L, Roos EM, Overgaard S, et al. Supervised neuromuscular exercise prior to hip and knee replacement: 12-month clinical effect and cost-utility analysis alongside a randomised controlled trial. *BMC Musculoskelet Disord* 2017;18(1):5. doi: https://dx.doi.org/10.1186/s12891-016-1369-0
41. Courville XF, Tomek IM, Kirkland KB, et al. Cost-effectiveness of preoperative nasal mupirocin treatment in preventing surgical site infection in patients undergoing total hip and knee arthroplasty: a cost-effectiveness analysis. *Infection Control and Hospital Epidemiology* 2012;33(2):152-59. doi: 10.1086/663704
42. Jackson BR, Umlas J, AuBuchon JP. The cost-effectiveness of postoperative recovery of RBCs in preventing transfusion-associated virus transmission after joint arthroplasty. *Transfusion* 2000;40(9):1063-6.
43. Ramkumar DB, Ramkumar N, Tapp SJ, et al. Pharmacologic Hemostatic Agents in Total Joint Arthroplasty-A Cost-Effectiveness Analysis. *J Arthroplasty* 2018;33(7):2092-99.e9. doi: https://dx.doi.org/10.1016/j.arth.2018.02.068
44. Sonnenberg FA. A health economic analysis of autologous transfusion. *Infusionstherapie und Transfusionsmedizin* 2002;29(3):175-83.
45. Marques EMR, Blom AW, Lenguerrand E, et al. Local anaesthetic wound infiltration in addition to standard anaesthetic regimen in total hip and knee replacement: Long-term cost-effectiveness analyses alongside the APEX randomised controlled trials. *BMC Medicine* 2015;13:151. doi: 10.1186/s12916-015-0389-1
46. Cummins JS, Tomek IM, Kantor SR, et al. Cost-effectiveness of antibiotic-impregnated bone cement used in primary total hip arthroplasty. *Journal of Bone and Joint Surgery American volume* 2009;91(3):634-41. doi: 10.2106/JBJS.G.01029
47. Graves N, Wloch C, Wilson J, et al. A cost-effectiveness modelling study of strategies to reduce risk of infection following primary hip replacement based on a systematic review. *Health Technology Assessment (Winchester, England)* 2016;20(54):1-144. doi: 10.3310/hta20540
48. Merollini KM, Crawford RW, Whitehouse SL, et al. Surgical site infection prevention following total hip arthroplasty in Australia: a cost-effectiveness analysis. *American Journal of Infection Control* 2013;41(9):803-9. doi: 10.1016/j.ajic.2012.11.015
49. Nherera LM, Trueman P, Karlakki SL. Cost-effectiveness analysis of single-use negative pressure wound therapy dressings (sNPWT) to reduce surgical site complications (SSC) in routine

For peer review only - http://bmjopen.bmj.com/site/about/guidelines.xhtml

primary hip and knee replacements. *Wound Repair Regen* 2017;25(3):474-82. doi: https://dx.doi.org/10.1111/wrr.12530
50. Fusco F, Turchetti G. Telerehabilitation after total knee replacement in Italy: cost-effectiveness and cost-utility analysis of a mixed telerehabilitation-standard rehabilitation programme compared with usual care. *BMJ Open* 2016;6(5):e009964. doi: 10.1136/bmjopen-2015-009964
51. Kauppila AM, Sintonen H, Aronen P, et al. Economic evaluation of multidisciplinary rehabilitation after primary total knee arthroplasty based on a randomized controlled trial. *Arthritis care & research* 2011;63(3):335-41. doi: 10.1002/acr.20398
52. Smith KC, Paltiel AD, Yang HY, et al. Cost-effectiveness of health coaching and financial incentives to promote physical activity after total knee replacement. *Osteoarthritis Cartilage* 2018;26(11):1495-505. doi: https://dx.doi.org/10.1016/j.joca.2018.07.014
53. Bolz KMD, Crawford RW, Donnelly B, et al. The cost-effectiveness of routine follow-up after primary total hip arthroplasty. *J Arthroplasty* 2010;25(2):191-96. doi: 10.1016/j.arth.2008.12.009
54. Larsen K, Sørensen OG, Hansen TB, et al. Accelerated perioperative care and rehabilitation intervention for hip and knee replacement is effective: a randomized clinical trial involving 87 patients with 3 months of follow-up. *Acta Orthop* 2008;79(2):149-59. doi: 10.1080/17453670710014923 [published Online First: 2008/05/15]
55. Kauppila A-M, Kyllönen E, Ohtonen P, et al. Multidisciplinary rehabilitation after primary total knee arthroplasty: a randomized controlled study of its effects on functional capacity and quality of life. *Clinical rehabilitation* 2010;24(5):398-411. doi: 10.1177/0269215509346089
56. Villadsen A, Overgaard S, Holsgaard-Larsen A, et al. Postoperative effects of neuromuscular exercise prior to hip or knee arthroplasty: a randomised controlled trial. *Annals of the rheumatic diseases* 2014;73(6):1130-7. doi: 10.1136/annrheumdis-2012-203135 [published Online First: 2013/05/11]
57. Wylde V, Lenguerrand E, Gooberman-Hill R, et al. Effect of local anaesthetic infiltration on chronic postsurgical pain after total hip and knee replacement: the APEX randomised controlled trials. *Pain* 2015;156(6):1161-70. doi: 10.1097/j.pain.0000000000000114
58. Briggs A, Sculpher M, Britton A, et al. The costs and benefits of primary total hip replacement. How likely are new prostheses to be cost-effective? *International journal of technology assessment in health care* 1998;14(4):743-61. [published Online First: 1999/01/14]
59. Birkmeyer JD, Goodnough LT, AuBuchon JP, et al. The cost-effectiveness of preoperative autologous blood donation for total hip and knee replacement. *Transfusion* 1993;33(7):544-51.
60. Sonnenberg FA, Gregory P, Yomtovian R, et al. The cost-effectiveness of autologous transfusion revisited: implications of an increased risk of bacterial infection with allogeneic transfusion. *Transfusion* 1999;39(8):808-17.
61. Losina E, Walensky RP, Kessler CL, et al. Cost-effectiveness of total knee arthroplasty in the United States: patient risk and hospital volume. *Archives of internal medicine* 2009;169(12):1113-21; discussion 21-2. doi: 10.1001/archinternmed.2009.136 [published Online First: 2009/06/24]
62. Jones E, Wainwright T, Foster J, et al. A systematic review of patient reported outcomes and patient experience in enhanced recovery after orthopaedic surgery. *The Annals of The Royal College of Surgeons of England* 2014;96(2):89-94. doi: 10.1308/003588414x13824511649571
63. Stowers MD, Lemanu DP, Hill AG. Health economics in Enhanced Recovery After Surgery programs. *Canadian journal of anaesthesia = Journal canadien d'anesthesie* 2015;62(2):219-30. doi: 10.1007/s12630-014-0272-0 [published Online First: 2014/11/14]
64. Lemanu DP, Singh PP, Stowers MD, et al. A systematic review to assess cost effectiveness of enhanced recovery after surgery programmes in colorectal surgery. *Colorectal disease : the*

For peer review only - http://bmjopen.bmj.com/site/about/guidelines.xhtml

official journal of the Association of Coloproctology of Great Britain and Ireland 2014;16(5):338-46. doi: 10.1111/codi.12505 [published Online First: 2013/11/29]
65. Lee L, Li C, Landry T, et al. A systematic review of economic evaluations of enhanced recovery pathways for colorectal surgery. *Annals of surgery* 2014;259(4):670-6. doi: 10.1097/SLA.0b013e318295fef8 [published Online First: 2013/05/16]
66. Köksal I, Tahta M, Şimşek ME, et al. Efficacy of rapid recovery protocol for total knee arthroplasty: a retrospective study. *Acta Orthopaedica et Traumatologica Turcica* 2015;49(4):382-6. doi: 10.3944/AOTT.2015.14.0353
67. Garriga C, Murphy J, Leal J, et al. Impact of a national enhanced recovery after surgery programme on patient outcomes of primary total knee replacement: an interrupted time series analysis from "The National Joint Registry of England, Wales, Northern Ireland and the Isle of Man". *Osteoarthritis Cartilage* 2019;27(9):1280-93. doi: 10.1016/j.joca.2019.05.001 [published Online First: 2019/05/13]
68. Hart A, Khalil JA, Carli A, et al. Blood transfusion in primary total hip and knee arthroplasty. Incidence, risk factors, and thirty-day complication rates. *J Bone Joint Surg Am* 2014;96(23):1945-51. doi: 10.2106/JBJS.N.00077
69. Norfolk D, editor. *Handbook of transfusion medicine*. Norwich, UK: TSO, 2013.
70. Simpson JC, Moonesinghe SR, Grocott MPW, et al. Enhanced recovery from surgery in the UK: an audit of the enhanced recovery partnership programme 2009–2012. *BJA: British Journal of Anaesthesia* 2015;115(4):560-68. doi: 10.1093/bja/aev105
71. Panella M, Marchisio S, Di Stanislao F. Reducing clinical variations with clinical pathways: do pathways work? *International journal for quality in health care : journal of the International Society for Quality in Health Care* 2003;15(6):509-21. doi: 10.1093/intqhc/mzg057 [published Online First: 2003/12/09]
72. Rotter T, Kinsman L, James E, et al. Clinical pathways: effects on professional practice, patient outcomes, length of stay and hospital costs. *The Cochrane database of systematic reviews* 2010(3):CD006632. doi: 10.1002/14651858.CD006632.pub2 [published Online First: 2010/03/20]

For peer review only

For peer review only - http://bmjopen.bmj.com/site/about/guidelines.xhtml

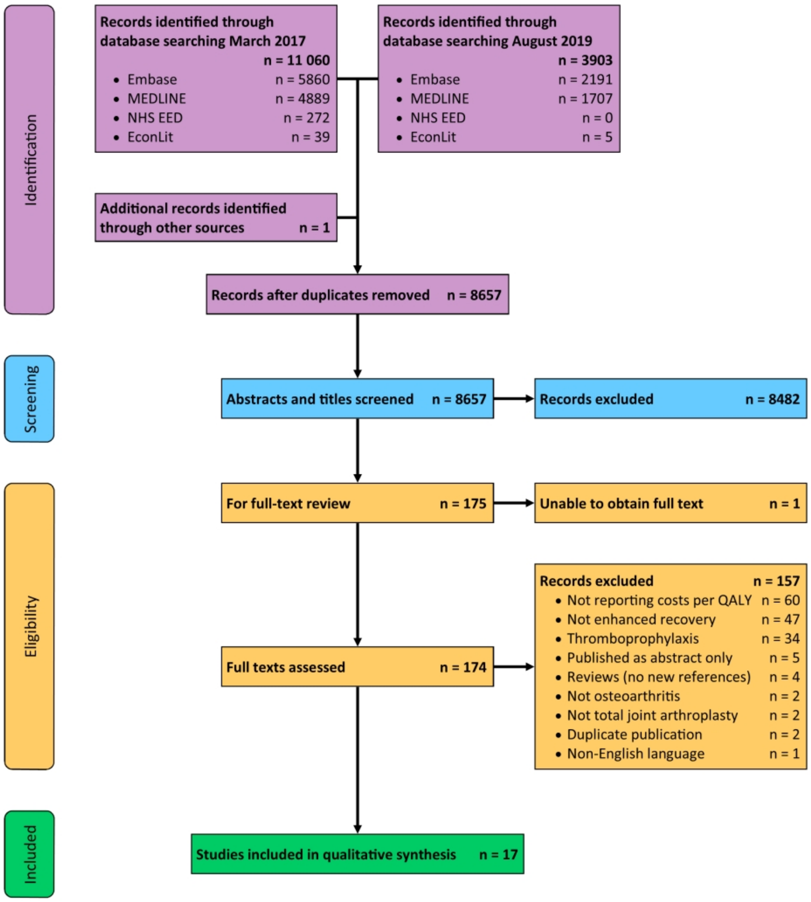

Figure 1. Flow diagram of studies included in this review and reasons for exclusion, modified from Moher, et al.28 (NHS EED, National Health Service Economic Evaluations Database; QALY, quality-adjusted life year)

209x297mm (300 x 300 DPI)

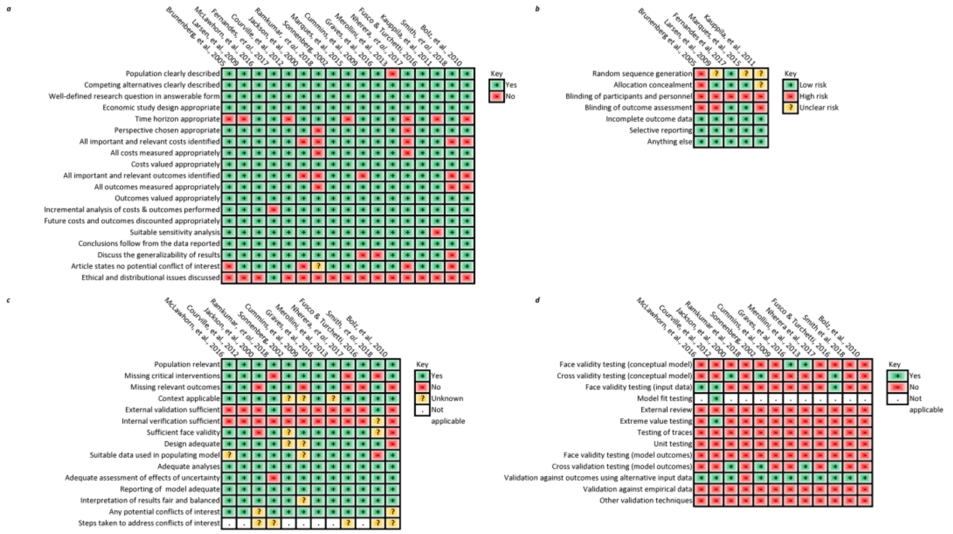

Figure 2. Assessments of study quality based on tools from (a) Consensus on Health Economic Criteria,30 (b) International Society for Pharmacoeconomics and Outcomes Research,31 (c) Assessment of the Validation Status of Health-Economic decision models tool,32 (d) Cochrane Collaboration.33 Note that the study by Brunenberg and colleagues37 was a non-randomised before and after trial and we have included it in Figure 2d for completeness.

297x209mm (300 x 300 DPI)

Supplemental Material: Enhanced recovery following hip and knee arthroplasty a systematic review of cost-effectiveness evidence. BMJ Open

Table A1. Full search terms for databases included in this review

Query	Search term
Ovid MEDLINE	
1	arthroplasty, replacement, hip/ or arthroplasty, replacement, knee/
2	((knee? or hip) adj (replace$ or arthroplast*)).ti,ab.
3	1 or 2
4	simulation model$.ti,ab.
5	markov.ti,ab.
6	monte carlo.ti,ab.
7	decision tree$.ti,ab.
8	decision analy$.ti,ab.
9	qaly$.ti,ab.
10	(valu$ adj2 quality).ti,ab.
11	utility value$.ti,ab.
12	((disability or quality) adj adjusted).ti,ab.
13	((life adj2 year$) or health year equivalent$).ti,ab.
14	(health adj utilit$).ti,ab.
15	hui$1.ti,ab.
16	(quality adj3 well$).ti,ab.
17	qwb.ti,ab.
18	(qald$ or qale$ or qtime$).ti,ab.
19	(well being or wellbeing).tw.
20	(health adj2 stat$).tw.
21	((adjusted adj2 life) or qaly$).ti,ab.
22	(daly or qol or hql or hqol or hrqol or hr ql or hrql).tw.
23	cost-utility.ti,ab.
24	cost-effectiveness.ti,ab.
25	cost-benefit.ti,ab.
26	cost-minimisation.ti,ab.
27	cost-minimization.ti,ab.
28	modelling.ti,ab.
29	modeling.ti,ab.
30	decision model.ti,ab.
31	QALY.ti,ab.
32	quality adjusted life year$.ti,ab.
33	cost.ti,ab.
34	life year$.ti,ab.
35	incremental cost-effectiveness ratio.ti,ab.
36	(quality adj2 life).ti,ab.
37	Technology Assessment, Biomedical/
38	"Costs and Cost Analysis"/
39	technology assessment$.ti,ab.
40	economic evaluation$.ti,ab.
41	economic model$.ti,ab.
42	discrete event simulat$.ti,ab.
43	cost utility.ti,ab.
44	cost effectiv$.ti,ab.
45	cost benefit.ti,ab.
46	cost minimisation.ti,ab.
47	cost minimization.ti,ab.
48	ICER$.ti,ab.
49	EQ-5D$.ti,ab.
50	(SF-12 or SF12 or Short Form 12).ti,ab.
51	(SF-36 or SF36 or Short Form 36).ti,ab.
52	(SF-6D or SF6D or Short Form 6D).ti,ab.

For peer review only - http://bmjopen.bmj.com/site/about/guidelines.xhtml

Supplemental Material: Enhanced recovery following hip and knee arthroplasty a systematic review of cost-effectiveness evidence. BMJ Open

53	rosser index.ti,ab.
54	person trade off.ti,ab.
55	standard gamble.ti,ab,kw.
56	time trade off.ti,ab,kw.
57	Hye.ti,ab,kw.
58	Hyes.ti,ab,kw.
59	Euroquol.ti,ab,kw.
60	4 or 5 or 6 or 7 or 8 or 9 or 10 or 11 or 12 or 13 or 14 or 15 or 16 or 17 or 18 or 19 or 20 or 21 or 22 or 23 or 24 or 25 or 26 or 27 or 28 or 29 or 30 or 31 or 32 or 33 or 34 or 35 or 36 or 37 or 38 or 39 or 40 or 41 or 42 or 43 or 44 or 45 or 46 or 47 or 48 or 49 or 50 or 51 or 52 or 53 or 54 or 55 or 56 or 57 or 58 or 59
61	3 and 60
EMBASE	
1	hip replacement/ or hip arthroplasty/
2	total knee replacement/ or knee replacement/ or knee arthroplasty/
3	((knee? or hip) adj (replace$ or arthroplast$)).ti,ab.
4	1 or 2 or 3
5	simulation model$.ti,ab.
6	markov.ti,ab.
7	monte carlo.ti,ab.
8	decision tree$.ti,ab.
9	decision analy$.ti,ab.
10	qaly$.ti,ab.
11	(valu$ adj2 quality).ti,ab.
12	utility value$.ti,ab.
13	((disability or quality) adj adjusted).ti,ab.
14	((life adj2 year$) or health year equivalent$).ti,ab.
15	hui$1.ti,ab.
16	(quality adj3 well$).ti,ab.
17	qwb.ti,ab.
18	(qald$ or qale$ or qtime$).ti,ab.
19	(well being or wellbeing).tw.
20	(health adj2 stat$).tw.
21	((adjusted adj2 life) or qaly$).ti,ab.
22	(daly or qol or hql or hqol or hrqol or hr ql or hrql).tw.
23	cost-utility.ti,ab.
24	cost-benefit.ti,ab.
25	cost-minimisation.ti,ab.
26	cost-minimization.ti,ab.
27	modelling.ti,ab.
28	modeling.ti,ab.
29	QALY.ti,ab.
30	quality adjusted life year$.ti,ab.
31	cost.ti,ab.
32	life year$.ti,ab.
33	incremental cost-effectiveness ratio.ti,ab.
34	(quality adj2 life).ti,ab.
35	decision model$.ti,ab.
36	cost-effectiv$.ti,ab.
37	"cost benefit analysis"/
38	biomedical technology assessment/
39	technology assessment$.ti,ab.
40	economic evaluation$.ti,ab.
41	economic model$.ti,ab.
42	discrete event simulat$.ti,ab.
43	cost utility.ti,ab.

For peer review only

For peer review only - http://bmjopen.bmj.com/site/about/guidelines.xhtml

Supplemental Material: Enhanced recovery following hip and knee arthroplasty a systematic review of cost-effectiveness evidence. BMJ Open

44	cost effectiv$.ti,ab.
45	cost benefit.ti,ab.
46	cost minimisation.ti,ab.
47	cost minimization.ti,ab.
48	ICER$.ti,ab.
49	(health adj utilit$).ti,ab.
50	EQ-5D$.ti,ab.
51	(SF-12 or SF12 or Short Form 12).ti,ab.
52	(SF-36 or SF36 or Short Form 36).ti,ab.
53	(SF-6D or SF6D or Short Form 6D).ti,ab.
54	rosser index.ti,ab.
55	person trade off.ti,ab.
56	standard gamble.ti,ab,kw.
57	time trade off.ti,ab,kw.
58	Hye.ti,ab,kw.
59	Hyes.ti,ab,kw.
60	Euroquol.ti,ab,kw.
61	5 or 6 or 7 or 8 or 9 or 10 or 11 or 12 or 13 or 14 or 15 or 16 or 17 or 18 or 19 or 20 or 21 or 22 or 23 or 24 or 25 or 26 or 27 or 28 or 29 or 30 or 31 or 32 or 33 or 34 or 35 or 36 or 37 or 38 or 39 or 40 or 41 or 42 or 43 or 44 or 45 or 46 or 47 or 48 or 49 or 50 or 51 or 52 or 53 or 54 or 55 or 56 or 57 or 58 or 59 or 60
62	4 and 61
National Health Service Economic Evaluations Database, via the Cochrane Library	
Hip	Title, Abstract, Keywords: “Hip arthroplasty” OR<br>Title, Abstract, Keywords: “Hip arthroplasties” OR<br>Title, Abstract, Keywords: “Hip replacement”
Knee	Title, Abstract, Keywords: “Knee arthroplasty” OR<br>Title, Abstract, Keywords: “Knee arthroplasties” OR<br>Title, Abstract, Keywords: “Knee replacement”
EconLit	
	TI,AB(hip) OR TI,AB(knee) AND<br>TI,AB(Replace*) OR TI,AB(arthroplasty*) OR TI,AB(Replacement) OR TI,AB(arthroplasties)

For peer review only - http://bmjopen.bmj.com/site/about/guidelines.xhtml

Supplemental Material: Enhanced recovery following hip and knee arthroplasty a systematic review of cost-effectiveness evidence. BMJ Open

Table A2. Hierarchy used to classify data sources used in studies, modified from Cooper and colleagues[34] and Coyle and colleagues[35]

*Source of data for clinical effect sizes, adverse events & complications**	
1	Meta-analysis of RCTs with direct comparison between comparator therapies, measuring final outcomes
2	Single RCT with direct comparison between comparator therapies, measuring final outcomes
3	Meta-analysis of RCTs with direct comparison between comparator therapies, measuring surrogate outcomes; *or*<br>meta-analysis of placebo-controlled RCTs with similar trial populations, measuring final outcomes for each individual therapy
4	Single RCT with direct comparison between comparator therapies, measuring surrogate outcomes; *or*<br>single placebo-controlled RCTs with similar trial populations, measuring final outcomes for each individual therapy
5	Meta-analysis of placebo-controlled RCTs with similar trial populations, measuring surrogate outcomes
6	Single placebo-controlled RCTs with similar trial populations, measuring surrogate outcomes for each individual therapy
7	Case–control or cohort studies
8	Non-analytic studies, for example, case reports, case series
9	Expert opinion
*Source of baseline clinical data**	
1	Case series or analysis of reliable administrative databases specifically conducted for the study covering patients solely from the jurisdiction of interest
2	Recent case series or analysis of reliable administrative databases covering patients solely from the jurisdiction of interest
3	Recent case series or analysis of reliable administrative databases covering patients solely from another jurisdiction
4	Old case series or analysis of reliable administrative databases; *or*<br>estimates from RCTs
5	Estimates from previously published economic analyses: unsourced
6	Expert opinion
*Source of data for duration of primary effect**	
1	Analysis of reliable administrative databases specifically conducted for the study covering patients solely from the jurisdiction of interest
2	Recent analysis of reliable administrative databases covering patients solely from the jurisdiction of interest
3	Recent analysis of reliable administrative databases covering patients solely from another jurisdiction
4	Old analysis of reliable administrative databases
5	Estimates from previously published economic analyses: unsourced
6	Expert opinion
*Source of data for resource use**	
1	Prospective data collection; *or*<br>analysis of reliable administrative data from same jurisdiction for specific study
2	Recently published results of prospective data collection from the same; *or*<br>recent analysis of reliable administrative data from the same jurisdiction
3	Unsourced data from previous economic evaluations from the same jurisdiction
4	Recently published results of prospective data collection from a different jurisdiction; *or*<br>recent analysis of reliable administrative data from a different jurisdiction
5	Unsourced data from previous economic evaluation from a different jurisdiction
6	Expert opinion
Source of data for costs	
1	Cost calculations based on reliable databases or data sources conducted for specific study—same jurisdiction
2	Recently published cost calculations based on reliable databases or data sources—same jurisdiction

For peer review only - http://bmjopen.bmj.com/site/about/guidelines.xhtml

Supplemental Material: Enhanced recovery following hip and knee arthroplasty a systematic review of cost-effectiveness evidence. BMJ Open

3	Unsourced data from previous economic evaluation—same jurisdiction
4	Recently published cost calculations based on reliable databases or data sources—different jurisdiction
5	Unsourced data from previous economic evaluation—different jurisdiction
6	Expert opinion
Source of data for utilities	
1	Direct utility assessment for the specific study from a sample either:<br>of the general population, or<br>with knowledge of the disease(s) of interest, or<br>of patients with the disease(s) of interest; *or*<br>indirect utility assessment for the specific study from patient sample with disease(s) of interest, using a tool validated for the patient population
2	Direct utility assessment from a previous study from a sample either:<br>of the general population, or<br>with knowledge of the disease(s) of interest, or<br>of patients with the disease(s) of interest; *or*<br>indirect utility assessment from a previous study from patient sample with disease(s) of interest, using a tool validated for the patient population
3	Indirect utility assessment from a patient sample with disease(s) of interest, using a tool not validated for the patient population; *or*<br>patient preference values obtained from a visual analogue scale
4	Delphi panels; *or*<br>expert opinion

* Hierarchy used for assessment of models only.
RCT, randomized controlled trial

For peer review only

For peer review only - http://bmjopen.bmj.com/site/about/guidelines.xhtml

Supplemental Material: Enhanced recovery following hip and knee arthroplasty a systematic review of cost-effectiveness evidence. BMJ Open

Table A3. Overview of studies included in the review.

Authors	Population	Perspective	Horizon	Discounting	Currency, year
**Enhanced recovery pathway**					
Brunenberg 2005[37]	Patients on the waiting list for either a THA (48 patients recruited to Joint Recovery Programme (JRP), 50 to usual care) or TKA (30 patients to JRP, 32 to usual care), mean age 64.4 years, one-third male	Societal	1 year	None	USD, 2002
Larsen 2009[38]	Patients in trial[50] having THA, THA or UKA, mean age having accelerated intervention 64, 26 women, 30 men	Societal	1 year	None	DKK, 2006
**Preoperative:** *Assessment and optimization of comorbidities*					
McLawhorn 2016[39]	50-year-old morbidly obese patient with end-stage unilateral knee osteoarthritis	Societal	40 years	3%	USD, 2012
**Preoperative:** *Exercise*					
Fernandes 2017[40]	Adults having unilateral THA or TKA for symptomatic osteoarthritis	Healthcare sector	61 weeks	None	EUR, 2012
**Preoperative:** *Staphylococcus aureus prophylaxis*					
Courville 2012[41]	65-year-olds with end stage hip or knee osteoarthritis for whom medical management has failed and TJA recommended.	Societal	1 year	None	USD, 2005
**Intraoperative:** *Avoid unnecessary blood transfusion*					
Jackson 2000[42]	Patients undergoing TJA, average age 65 years, range 20 to 80 years	Not stated	Lifetime	3% and 5%	USD, not reported
Ramkumar 2018[43]	65-year-old having TJA for severe osteoarthritits	Medical costs	10 years	3%	
Sonnenberg 2002[44]	65-year-old of composite sex and race undergoing THA	Not stated	Lifetime	3%	USD, 2000
**Intraoperative:** *Local infiltration of analgesia*					
Marques 2015[45]	Patients in trial[51] having THA or TKA, mean ages 66–69.5 years, 52%–65% female	Health and social care payer	1 year	None	GBP, not reported
**Intraoperative:** *Infection prevention*					
Cummins 2009[46]	68-year-olds of average health for their age undergoing THA due to degenerative arthritis	Hospital	Lifetime	3%	USD, 2002
Graves 2016[47]	Simulated cohort of patients who had THA in 2012	UK NHS	Lifetime	3%	GBP, 2012
Merollini 2013[48]	Not described	Healthcare system	30 years	3%	AUD, 2011
Nherera 2017[49]	Adults having routine TJA	UK NHS	6 weeks	None	GBP, 2015
**Postoperative:** *Physical therapy*					
Fusco & Turchetti, 2016[50]	Average age of 60 years, 44% males, 19% experienced complications during or after surgery	Societal & healthcare service	Lifetime	3%	Euro, 2013
Smith 2018[52]	Patients having unilateral TKA for knee osteoarthritis	Healthcare sector	Lifetime	3%	USD, 2016
Kauppila 2011[51]	60–80 year olds having unilateral TKR for knee osteoarthritis	Healthcare system	1 year	None	Euro, 2006

For peer review only - http://bmjopen.bmj.com/site/about/guidelines.xhtml

Supplemental Material: Enhanced recovery following hip and knee arthroplasty a systematic review of cost-effectiveness evidence. BMJ Open

**Postoperative:** *Scheduling of follow-up*					
Bolz 2010[53]	THA patients, mean age 69.9 years	Health services	7 years	3%	AUD, not reported
NHS: National Health Service; THA: total hip arthroplasty; TJA: total joint arthroplasty; TKA: total knee arthroplasty; UKA: unicompartmental knee arthroplasty; AUD: Australian dollar; DKK: Danish krone; GBP: British pound; USD: US dollar					

For peer review only

For peer review only - http://bmjopen.bmj.com/site/about/guidelines.xhtml

Supplemental Material: Enhanced recovery following hip and knee arthroplasty a systematic review of cost-effectiveness evidence. BMJ Open

Table A4. Costs included in each study

	Direct medical	Direct treatment	In-patient	Out-patient	Day care	Community healthcare	Medication	Side effect costs	Staff	Labs/diagnostic	Capital equipment	Social care	Travel costs	Productivity losses	Income forgone due to illness
Brunenberg 2005[37]	+	+	+	+		+	+		+			+		+	+
Larsen 2009[38]	+	+	+	+		+	+		+					+	+
McLawhorn 2016[39]	+	+	+					+							
Fernandes 2017[40]	+	+							+				+		+
Courville 2012[41]	+	+	+				+			+					
Jackson 2000[42]	+	+	+	+			+		+	+					
Ramkumar 2018[43]	+	+					+								
Sonnenberg 2002[44]	+	+	+	+			+								
Marques 2015[45]	+	+	+	+	+	+	+		+			+			
Cummins 2009[46]	+	+	+												
Graves 2016[47]	+	+	+				+				+				
Merollini 2013[48]	+	+	+				+		+		+				
Nherera 2017[49]	+	+	+	+		+	+								
Fusco & Turchetti, 2016[50]	+	+	+	+					+		+		+	+	+
Kauppila 2011[51]	+	+	+	+	+	+						+			
Smith 2018[52]	+	+					+	+							
Bolz 2010[53]	+		+	+						+					

For peer review only

For peer review only - http://bmjopen.bmj.com/site/about/guidelines.xhtml

Supplemental Material: Enhanced recovery following hip and knee arthroplasty a systematic review of cost-effectiveness evidence. BMJ Open

Table A5. Populations and tools used for eliciting utilities.

Authors	EQ-5D	15 D	QWB	SG	VAS / rating scale	TTO	AQoL	Other
Studies eliciting utilities								
Brunenberg 2005[37]	Patients in trial (UK tariff)	—	—	—	—	—	—	—
Larsen 2009[38]	Patients in trial (Danish tariff)	—	—	—	—	—	—	—
Fernandes 2017[40]	Patients in trial (Danish tariff)	—	—	—	—	—	—	—
Marques 2015[45]	Patients in trial (UK tariff)	—	—	—	—	—	—	—
Fusco & Turchetti, 2016[50]	TKR patients in trial (UK tariff)	—	—	—	—	—	—	—
Smith 2018[52]	Patients in trial (tariff not reported)	—	—	—	—	—	—	—
Kauppila 2011[50]	—	Patients in trial (Finnish tariff)	—	—	—	—	—	—
Studies using published utilities[*]								
McLawhorn 2016[39]	TKR patients in a trial; patients having bariatric surgery	—	—	—	Patients having bariatric surgery	—	—	—
Courville 2012[41]	—	—	Population reporting arthritis	—	—	—	—	—
Jackson 2000[42]	—	—	People with HIV infection	People with HIV infection	People with HIV infection	People with HIV infection	—	—
Ramkumar 2018[43]	—	—	—	—	—	—	—	Several sources
Sonnenberg 2002[45]	—	—	People with HIV infection	—	—	—	—	—
Cummins 2009[46]	—	—	—	—	—	THA patients	—	—

For peer review only - http://bmjopen.bmj.com/site/about/guidelines.xhtml

For peer review only

Supplemental Material: Enhanced recovery following hip and knee arthroplasty a systematic review of cost-effectiveness evidence. BMJ Open

Authors	EQ-5D	15 D	QWB	SG	VAS / rating scale	TTO	AQoL	Other
Graves 2016[47]	THA patients	THA patients	—	—	—	—	THA patients with prosthesis infection	—
Merollini 2013[48]	—	THA patients	—	—	—	—	THA patients with prosthesis infection	—
Nherera 2017[49]	—	—	—	—	—	—	—	SF-36 in patients undergoing orthopaedic surgery
Bolz 2010[53]	—	THA patients	—	—	—	—	—	—

* Details of utilities obtained from expert opinion are not included in this table
15-D, 15-dimension instrument; AQoL, Assessment of Quality of Life; EQ-5D, EuroQoL five-dimension instrument; HIV, human immunodeficiency virus; SF-36, Short Form (36) Health Survey; SG, standard gamble; THA, total hip arthroplasty; TKA, total knee arthroplasty; TTO, time trade-off; QWB, Quality of Well-being scale; VAS, visual analogue scale

For peer review only

Supplemental Material: Enhanced recovery following hip and knee arthroplasty a systematic review of cost-effectiveness evidence. BMJ Open

Table A6. Overview of quality and sources of data used by studies included in this analysis

	Sources of data											
	Clinical effect sizes		Baseline clinical data		Duration of primary effect		Resource use		Costs		Utilities	
Authors	Min	Max	Min	Max	Min	Max	Min	Max	Min	Max	Min	Max
Range of possible scores[*]	*1*	*9*	*1*	*6*	*1*	*6*	*1*	*6*	*1*	*6*	*1*	*4*
Brunenberg 2005[37]	—†	—†	—†	—†	—†	—†	—†	—†	1	2	1	1
Larsen 2009[38]	—†	—†	—†	—†	—†	—†	—†	—†	1	4	1	1
McLawhorn 2016[39]	1	8	2	2	NR	NR	NR	NR	2	6	2	4
Fernandes 2017[40]	—†	—†	—†	—†	—†	—†	—†	—†	1	1	1	1
Courville 2012[41]	1	1	1	1	NR	NR	2	2	2	2	2	2
Jackson 2000[42]	8	8	2	2	NR	NR	NR	NR	4	4	2	2
Ramkumar 2018[43]	1	8	4	4	6	6	4	4	4	4	1	4‡
Sonnenberg 2002[44]	8	8	2	2	NR	NR	3	3	1	3	2	4
Marques 2015[45]	—†	—†	—†	—†	—†	—†	—†	—†	1	2	1	1
Cummins 2009[46]	8	8	3	3	NR	NR	2	2	2	4	2	2
Graves 2016[47]	1	7	1	4	NR	NR	2	6	1	1	2	4
Merollini 2013[48]	1	7	1	3	NR	NR	NR	NR	1	6	2	4
Nherera 2017[49]	2	2	4	4	NA‖	NA‖	2	2	2	2	2	2
Fusco & Turchetti, 2016[50]	3	3	2	5	3	3	2	2	2	6	2	2
Kauppila 2011[51]	—†	—†	—†	—†	—†	—†	—†	—†	1	2	1	1
Smith 2018[52]	2	2	4	4	6	6	2	2	2	2	1	1
Bolz 2010[53]	NR	NR	2	2	NR	NR	NR	NR	2	2	2	2

Note: Where a study used several data sources within each category the results are reported as a range of the minimum to maximum data quality score within each category.

* Details of the full hierarchy are available in supplementary table A2 online.

† Not applicable as study is not model-based

‡ Utilities from patients with other conditions

‖ Model time horizon limited to duration of study providing clinical effect sizes

CHEC, Consensus on Health Economic Criteria; ISPOR, International Society for Pharmacoeconomics and Outcomes Research; NA, not applicable; NR, not reported

For peer review only

For peer review only - http://bmjopen.bmj.com/site/about/guidelines.xhtml

Supplemental Material: Enhanced recovery following hip and knee arthroplasty a systematic review of cost-effectiveness evidence. BMJ Open

Table A7. Summary of findings from studies included in this analysis

Authors, country	Population	Strategy	Cost	Outcome in QALYs	ICER in cost per QALY*	Probability of cost-effectiveness (threshold in cost per QALY)
**Enhanced care pathway**						
Brunenberg, *et al.*[37] Netherlands	THA	Conventional care	USD 11 312	0.65		
		Joint Recovery Programme (pre- assessment and intensive rehabilitation)	USD 10 051	0.70		
		Increment	−1261	0.07†	Dominates	0.94 (USD 45 000)
	TKA	Conventional care	USD 12 877	0.61		
		Joint Recovery Programme (pre-operative assessment and intensive rehabilitation)	USD 8541	0.65		
		Increment	−3336	0.04†	Dominates	0.99 (USD 45 000)
Larsen, *et al.*,[38] Denmark	THA + TKA	Conventional care	DKK 90 227	0.78		
		Accelerated perioperative care and rehabilitation	DKK 71 344	0.83		
		Increment	−18 880	0.05	Dominates	0.97‡ (DKK 160 000)
	THA	Conventional care	DKK 87 657	0.75		
		Accelerated perioperative care and rehabilitation	DKK 71 768	0.84		
		Increment	−15 889	0.09	Dominates	0.98‡ (DKK 160 000)
	TKA	Accelerated perioperative care and rehabilitation	DKK 70 644	0.81		
		Conventional care	DKK 95 367	0.85		
		Increment	24 723	0.04	DKK 618 075	NR

For peer review only - http://bmjopen.bmj.com/site/about/guidelines.xhtml

Supplemental Material: Enhanced recovery following hip and knee arthroplasty a systematic review of cost-effectiveness evidence. BMJ Open

Table A7. Continued.

Authors, country	Population	Strategy	Cost	Outcome in QALYs	ICER in cost per QALY*	Probability of cost-effectiveness (threshold in cost per QALY)
**Preoperative**						
Assessment and optimization of comorbidities						
McLawhorn, *et al.*,[39] US	Morbidly obese with unilateral end-stage knee osteoarthritis					
		Immediate TKA	USD 60 453	10.83		
		Bariatric surgery, followed by TKA 2 years later	USD 84 099	12.53		
		Increment	USD 23 646	1.70	USD 13 910	0.988 (USD 100 000)
Exercise						
Fernandes, *et al.*[40] Denmark	THA + TKA	Educational package	EUR 16 313	0.61		
		Supervised neuromuscular exercise + educational package	EUR 16 181	0.66		
		Increment	EUR −132	0.04[†]	Dominates	0.84 (EUR 40 000)
Staphylococcus aureus *prophylaxis*						
Courville, *et al.*,[41] US	THA	Standard infection prevention measures without *S. aureus* screening or mupirocin decolonization	USD 24 506	0.7980		
		Preoperative nasal screening for *S. aureus* followed by mupirocin treatment for patients with positive cultures	USD 24 471	0.7983	D	
		Empirical treatment of all preoperative patients with mupirocin	USD 24 258	0.7985		
		Increment (compared to standard measures)	−248	0.0005	Dominates	NR
	TKA	Standard infection prevention measures without *S. aureus* screening or mupirocin decolonization	USD 24 667	0.6783		
		Preoperative nasal screening for *S. aureus* followed by mupirocin treatment for patients with positive cultures	USD 24 611	0.6785	D	
		Empirical treatment of all preoperative patients with mupirocin	USD 24 378	0.6787		
		Increment (compared to standard measures)	−289	0.0004	Dominates	NR

For peer review only - http://bmjopen.bmj.com/site/about/guidelines.xhtml

Supplemental Material: Enhanced recovery following hip and knee arthroplasty a systematic review of cost-effectiveness evidence. BMJ Open

Authors, country	Population	Strategy	Cost	Outcome in QALYs	ICER in cost per QALY*	Probability of cost-effectiveness (threshold in cost per QALY)
**Intraoperative**						
Avoid unnecessary blood transfusion						
Jackson, *et al.*,[42] US	THA + TKA	Usual transfusion practice	NR	NR		
		Postoperative erythrocyte recovery and transfusion	NR	NR		
		Increment	USD 53	0.00001	USD 5 700 000	NR (USD 50 000)
Ramkumar, *et al.*,[43] US	THA + TKA	No pharmacologic haemostatic agent	USD 951	8.893		
		Single dose intravenous aminocaproic acid	USD 1175	9.152	D	
		Single dose intravenous tranexamic acid	USD 460	9.339		
		Increment (compared to no pharmacologic haemostatic agent)	USD −491	0.447	Dominates	NR (USD 100 000)
Sonnenberg,[44] US	THA	Usual practice without autologous donation	USD 1395	NR		
		Autologous blood donation and transfusion	USD 1539	NR		
		Increment	144	0.0523	USD 2750	NR (USD 50 000)
Local infiltration of analgesia						
Marques, *et al.*,[45] UK	THA	Standard anaesthesia	NR	NR		
		Intraoperative local anaesthetic wound infiltration administered before wound closure in addition to standard anaesthesia	NR	NR		
		Increment	GBP −86	0.052	Dominates	0.98 (GBP 20 000)
	TKA	Standard anaesthesia	NR	NR		
		Intraoperative local anaesthetic wound infiltration administered before wound closure in addition to standard anaesthesia	NR	NR		
		Increment	GBP −77	0.009	Dominates	0.60 (GBP 20 000)
Infection prevention						
Cummins, *et al.*,[46] US	THA	Conventional cement	USD 24 100	9.439		
		Antibiotic-impregnated bone cement	USD 23 900	9.454		
		Increment	−200	0.015	Dominates	NR

For peer review only - http://bmjopen.bmj.com/site/about/guidelines.xhtml

For peer review only

Supplemental Material: Enhanced recovery following hip and knee arthroplasty a systematic review of cost-effectiveness evidence. BMJ Open

Authors, country	Population	Strategy	Cost	Outcome in QALYs	ICER in cost per QALY*	Probability of cost-effectiveness (threshold in cost per QALY)
Graves, *et al.*,[47] UK[§]	THA	No systemic antibiotics, plain cement and conventional ventilation	GBP 0[§]	0[§]		
		Systemic antibiotics, antibiotic-impregnated cement, laminar ventilation and body exhaust suit	GBP 781 075	62	D	0.01[‖] (GBP 18 000)
		No systemic antibiotics, antibiotic-impregnated cement and conventional ventilation	GBP −4 634 647	89	D	0.07[‖] (GBP 18 000)
		Systemic antibiotics, plain cement and conventional ventilation	GBP −7 226 732	101	D	0.15[‖] (GBP 18 000)
		Systemic antibiotics, antibiotic-impregnated cement, conventional ventilation and body exhaust suit	GBP −3 960 897	106	D	0.11[‖] (GBP 18 000)
		Systemic antibiotics, plain cement and laminar airflow	GBP −5 271 040	118	D	0.10[‖] (GBP 18 000)
		Systemic antibiotics, antibiotic-impregnated cement and laminar airflow	GBP −6 152 877	124	D	0.06[‖] (GBP 18 000)
		No systemic antibiotics, plain cement and laminar airflow	GBP −3 271 749	124	D	0.18[‖] (GBP 18 000)
		Systemic antibiotics, antibiotic-impregnated cement and conventional ventilation	GBP −8 325 277	147		
		Increment (compared to systemic antibiotics, plain cement and conventional ventilation)	−1 098 545	46	Dominates	0.32[e] (GBP 18 000)
Merollini, *et al.*,[48] Australia	THA	No antibiotic prophylaxis	AUD 1 517 954	−163.1		
		Antibiotic prophylaxis and laminar airflow	AUD 4 592 200	−126.9	D	
		Antibiotic prophylaxis	AUD 0[¶]	0[¶]	D	
		Antibiotic prophylaxis and antibiotic-impregnated cement	AUD −126 375	32.3		
		Increment	−126 375	32.3	Dominates	0.986 (AUD 40 000)[**]
Nherera, *et al.*,[49] UK	THA + TKA	Usual care	GBP 6713	0.115		
		Single-use negative pressure wound therapy dressings	GBP 5602	0.116		
		Increment	GBP −1049	0.0014	Dominates	0.91 (GBP 20 000)

For peer review only - http://bmjopen.bmj.com/site/about/guidelines.xhtml

For peer review only

Supplemental Material: Enhanced recovery following hip and knee arthroplasty a systematic review of cost-effectiveness evidence. BMJ Open

Table A7. Continued.

Authors, country	Population	Strategy	Cost	Outcome in QALYs	ICER in cost per QALY*	Probability of cost-effectiveness (threshold in cost per QALY)
**Postoperative**						
Physical therapy						
Fusco & Turchetti,[50] Italy	TKA, societal perspective	20 face-to-face rehabilitation sessions	EUR 1315	13.02		
		10 face-to-face rehabilitation sessions plus 10 telesessions	EUR 977	13.02		
		Increment	−338	0	Dominates	NR (EUR 30 000)
	TKA, Italian NHS perspective	20 face-to-face rehabilitation sessions	EUR 1124	13.02		
		10 face-to-face rehabilitation sessions plus 10 telesessions	EUR 862	13.02		
		Increment	−262	0	Dominates	NR (EUR 30 000)
Kauppila, *et al.*,[51] Finland	TKA	Multidisciplinary biopsychosocial outpatient rehabilitation programme	EUR 12 950	NR		
		Conventional orthopaedic care	EUR 11 120	NR		
		Increment	−1830	0.0192	Dominates	NR
Smith, *et al.*,[52] US		Telephone calls conveying general health messages	USD 140 700	9.783		
		Telephonic health coaching and financial incentives	USD 141 000	9.788		
		Increment	USD 300	0.005	USD 57 200	0.70 (USD 100 000)
Scheduling of follow-up						
Bolz, *et al.*,[53] Australia††	THA, assuming 5% revisions delayed under no follow-up‡‡					
		2-yearly routine follow-up	AUD 26 426 908	147 940		
		Follow-up at 3 months and 1 or 2 years	AUD 21 331 518	147 940	D	
		No follow-up	AUD 14 867 616	147 949		
		Increment	−6 463 902	9	Dominates	NR

* Strategies listed in order of increasing effectiveness; ICER presented compared to next most effective strategy that is not extended-dominated; no ICER presented for dominated or extended-dominated strategies; ICERs have been calculated where not explicitly presented in the reference, where ICERs have been presented they may differ slightly from what would be calculated from the presented figures due to rounding.
† Adjusted for baseline quality of life using regression analysis
‡ Probability of being the dominant strategy

For peer review only - http://bmjopen.bmj.com/site/about/guidelines.xhtml

Supplemental Material: Enhanced recovery following hip and knee arthroplasty a systematic review of cost-effectiveness evidence. BMJ Open

§ Total costs and outcomes for a cohort of 77 321 patients, relative to the cost of the strategy of no systemic antibiotics, plain cement and conventional ventilation
‖ Probability of being most cost-effective strategy in the model
¶ Total costs and outcomes per 30 000 primary THRs, relative to the cost of the strategy of antibiotic prophylaxis
** Cost-effectiveness ≥98.6% at typical willingness-to-pay values of AUD 40 000 to AUD 64 000
†† Total costs and outcomes for a cohort of 30 440 patients
‡‡ Four different probabilities were modelled (1%, 5%, 10%, 50%), but the direction of the results did not change between them

AUD, Australian dollar; D, dominated; DKK, Danish krone; EUR, euro; GBP, British pound; ICER, incremental cost-effectiveness ratio; NHS, National Health Service; NR, not reported; QALY, quality-adjusted life year; THA, total hip arthroplasty; TKA, total knee arthroplasty; UK, United Kingdom; US, United States; USD, United States dollar

For peer review only

For peer review only - http://bmjopen.bmj.com/site/about/guidelines.xhtml

Supplemental Material: Enhanced recovery following hip and knee arthroplasty a systematic review of cost-effectiveness evidence. BMJ Open

Table A8. Components of enhanced recovery pathway in the trials of an entire pathway

	Larsen, *et al.*[38]		Brunenberg, *et al.*[37]	
**Intervention type**	**Standard protocol**	**Accelerated protocol**	**Usual care**	**Joint recovery programme**
Pre-operative assessment	NA	NA	No standardized screening	History, examination & blood testing 6 weeks prior to surgery
Pre-operative education	Information given to patients on admission	Information provided in groups at outpatient clinic prior to hospitalization	No information session	Information session 1–2 weeks prior to surgery
Day of admission	Day before surgery	Day of surgery	NA	NA
Bed allocation in hospital	Amongst other patients	Patients having joint replacement placed together in separate part of ward	NA	NA
Staff involved	Various	One nurse in charge of multidisciplinary team of nurses, OT & PT	NA	Supervised by PT & nurses throughout admission
Nutrition	Screening	Screening plus daily intake 1.5 L fluid & 2 protein drinks	NA	NA
Start of mobilization	1 day after surgery	Day of surgery	NA	NA
Description of mobilization	Individual & gradual mobilization according to patient tolerance	Intensive mobilization in teams with pre-set goals	Conventional physiotherapy	Rehabilitation in groups in a room resembling the home situation
Duration of mobilization	4 hours daily	8 hours daily	1 hour daily	Duration not specified
Discharge planning	NA	NA	During admission	6 weeks prior to surgery
Other details	NA	NA		Patients could involve a family member or friend to give emotional support

NA, not applicable; OT, occupational therapists; PT, physiotherapists

For peer review only - http://bmjopen.bmj.com/site/about/guidelines.xhtml

Supplemental Material: Enhanced recovery following hip and knee arthroplasty a systematic review of cost-effectiveness evidence. BMJ Open

Table A9. Willingness to pay thresholds and incremental cost-effectiveness ratios for all studies converted into international dollars

		Values as reported*		Adjusted values in international dollars[†]	
**Authors, country**	**Currency, cost year**	**WTP threshold**	**ICER (unit cost/QALY)**	**WTP threshold**	**ICER (unit cost/QALY)**
Brunenberg, *et al.*[37] Netherlands	USD, 2002	45 000	—[‡]	45 000	—[‡]
Larsen, *et al.*,[38] Denmark	DKK, 2006	160 000	618 075	22 122	99 624
McLawhorn, *et al.*,[39] US	USD, 2012	100 000	13 910	100 000	14 995
Fernandes, *et al.*[40] Denmark	EUR, 2012	40 000	—[‡]	41 720	—[‡]
Courville, *et al.*,[41] US	USD, 2005	NR	—[‡]	—	—[‡]
Jackson, *et al.*,[42] US	USD, 2000[§]	50 000	5 700 000	50 000	7 787 912
Ramkumar, *et al.*,[43] US	USD, 2016	100 000	—[‡]	100 000	—[‡]
Sonnenberg,[44] US	USD, 2000	50 000	2 750	50 000	3 757
Marques, *et al.*,[45] UK	GBP, 2015[§]	20 000	—[‡]	28 838	—[‡]
Cummins, *et al.*,[46] US	USD, 2002	NR	—[‡]	—	—[‡]
Graves, *et al.*,[47] UK	GBP, 2012	18 000	—[‡]	25 954	—[‡]
Merollini, *et al.*[48], Australia	AUD, 2011	40 000	—[‡]	27 287	—[‡]
Nherera, *et al.*,[49] UK	GBP, 2015	20 000	—[‡]	28 838	—[‡]
Fusco & Turchetti,[50] Italy	EUR, 2013	30 000	—[‡]	41 482	—[‡]
Kauppila, *et al.*,[51] Finland	EUR, 2006	NR	—[‡]	—	—[‡]
Smith, *et al.*,[52] US	USD, 2016	100 000	57 200	100 000	57 200
Bolz, *et al.*,[53] Australia	AUS, 2010[§]	NR	—[‡]	—	—[‡]

* Only non-dominating results reported
† Willingness to pay thresholds not adjusted for cost year as they have been constant in most countries since they were introduced, in spite of cost inflation; ICERs additionally adjusted to 2016 values
‡ Dominance reported for all strategies
§ Cost year not reported; assumed as publication year

AUD, Australian dollar; DKK, Danish krone; EUR, euro; GBP, British pound; ICER, incremental cost-effectiveness ratio; NR, not reported; QALY, quality-adjusted life year; UK, United Kingdom; US, United States; USD, United States dollar; WTP, willingness to pay

For peer review only

For peer review only - http://bmjopen.bmj.com/site/about/guidelines.xhtml

PRISMA 2009 Checklist

Section/topic	#	Checklist item	Reported on page #
**TITLE**			
Title	1	Identify the report as a systematic review, meta-analysis, or both.	1
**ABSTRACT**			
Structured summary	2	Provide a structured summary including, as applicable: background; objectives; data sources; study eligibility criteria, participants, and interventions; study appraisal and synthesis methods; results; limitations; conclusions and implications of key findings; systematic review registration number.	2
**INTRODUCTION**			
Rationale	3	Describe the rationale for the review in the context of what is already known.	5
Objectives	4	Provide an explicit statement of questions being addressed with reference to participants, interventions, comparisons, outcomes, and study design (PICOS).	5
**METHODS**			
Protocol and registration	5	Indicate if a review protocol exists, if and where it can be accessed (e.g., Web address), and, if available, provide registration information including registration number.	6
Eligibility criteria	6	Specify study characteristics (e.g., PICOS, length of follow-up) and report characteristics (e.g., years considered, language, publication status) used as criteria for eligibility, giving rationale.	6
Information sources	7	Describe all information sources (e.g., databases with dates of coverage, contact with study authors to identify additional studies) in the search and date last searched.	6
Search	8	Present full electronic search strategy for at least one database, including any limits used, such that it could be repeated.	6 and Supplementary Table A1
Study selection	9	State the process for selecting studies (i.e., screening, eligibility, included in systematic review, and, if applicable, included in the meta-analysis).	6
Data collection process	10	Describe method of data extraction from reports (e.g., piloted forms, independently, in duplicate) and any processes for obtaining and confirming data from investigators.	6
Data items	11	List and define all variables for which data were sought (e.g., PICOS, funding sources) and any assumptions and simplifications made.	6-7
Risk of bias in individual studies	12	Describe methods used for assessing risk of bias of individual studies (including specification of whether this was done at the study or outcome level), and how this information is to be used in any data synthesis.	7
Summary measures	13	State the principal summary measures (e.g., risk ratio, difference in means).	7
Synthesis of results	14	Describe the methods of handling data and combining results of studies, if done, including measures of consistency (e.g., I^2) for each meta-analysis.	7

For peer review only

PRISMA 2009 Checklist

Section/topic	#	Checklist item	Reported on page #
Risk of bias across studies	15	Specify any assessment of risk of bias that may affect the cumulative evidence (e.g., publication bias, selective reporting within studies).	7
Additional analyses	16	Describe methods of additional analyses (e.g., sensitivity or subgroup analyses, meta-regression), if done, indicating which were pre-specified.	Not applicable
**RESULTS**			
Study selection	17	Give numbers of studies screened, assessed for eligibility, and included in the review, with reasons for exclusions at each stage, ideally with a flow diagram.	9-10 and Figure 1
Study characteristics	18	For each study, present characteristics for which data were extracted (e.g., study size, PICOS, follow-up period) and provide the citations.	9-10, Table 1 and Supplementary Tables A3,A4 and A5
Risk of bias within studies	19	Present data on risk of bias of each study and, if available, any outcome level assessment (see item 12).	13, Figure 2 and Table A6
Results of individual studies	20	For all outcomes considered (benefits or harms), present, for each study: (a) simple summary data for each intervention group (b) effect estimates and confidence intervals, ideally with a forest plot.	14-20, Table 2 and Supplementary Table A7
Synthesis of results	21	Present results of each meta-analysis done, including confidence intervals and measures of consistency.	Not applicable
Risk of bias across studies	22	Present results of any assessment of risk of bias across studies (see Item 15).	13, Figure 2 and Table A6
Additional analysis	23	Give results of additional analyses, if done (e.g., sensitivity or subgroup analyses, meta-regression [see Item 16]).	Not applicable
**DISCUSSION**			
Summary of evidence	24	Summarize the main findings including the strength of evidence for each main outcome; consider their relevance to key groups (e.g., healthcare providers, users, and policy makers).	20-21
Limitations	25	Discuss limitations at study and outcome level (e.g., risk of bias), and at review-level (e.g., incomplete retrieval of identified research, reporting bias).	21-22
Conclusions	26	Provide a general interpretation of the results in the context of other evidence, and implications for future research.	23
**FUNDING**			
Funding	27	Describe sources of funding for the systematic review and other support (e.g., supply of data); role of funders for the systematic review.	4

For peer review only - http://bmjopen.bmj.com/site/about/guidelines.xhtml

PRISMA 2009 Checklist

From: Moher D, Liberati A, Tetzlaff J, Altman DG, The PRISMA Group (2009). Preferred Reporting Items for Systematic Reviews and Meta-Analyses: The PRISMA Statement. PLoS Med 6(7): e1000097. doi:10.1371/journal.pmed1000097

For peer review only

The PRISMA for Abstracts Checklist

TITLE	CHECKLIST ITEM	REPORTED ON PAGE #
1. Title:	Identify the report as a systematic review, meta-analysis, or both.	1
**BACKGROUND**		
2. Objectives:	The research question including components such as participants, interventions, comparators, and outcomes.	2
**METHODS**		
3. Eligibility criteria:	Study and report characteristics used as criteria for inclusion.	2
4. Information sources:	Key databases searched and search dates.	2
5. Risk of bias:	Methods of assessing risk of bias.	2
**RESULTS**		
6. Included studies:	Number and type of included studies and participants and relevant characteristics of studies.	2
7. Synthesis of results:	Results for main outcomes (benefits and harms), preferably indicating the number of studies and participants for each. If meta-analysis was done, include summary measures and confidence intervals.	2
8. Description of the effect:	Direction of the effect (i.e. which group is favoured) and size of the effect in terms meaningful to clinicians and patients.	2
**DISCUSSION**		
9. Strengths and Limitations of evidence:	Brief summary of strengths and limitations of evidence (e.g. inconsistency, imprecision, indirectness, or risk of bias, other supporting or conflicting evidence)	2
10. Interpretation:	General interpretation of the results and important implications	2
**OTHER**		
11. Funding:	Primary source of funding for the review.	5
12. Registration:	Registration number and registry name.	3
